# A Multi-Agency Nutrient Dataset Used to Estimate Loads, Improve Monitoring Design, and Calibrate Regional Nutrient SPARROW Models^1^

**DOI:** 10.1111/j.1752-1688.2011.00575.x

**Published:** 2011-10

**Authors:** David A Saad, Gregory E Schwarz, Dale M Robertson, Nathaniel L Booth

**Keywords:** rivers/streams, nutrients, monitoring, load estimation, statistics, SPARROW

## Abstract

**Abstract:**

Stream-loading information was compiled from federal, state, and local agencies, and selected universities as part of an effort to develop regional SPAtially Referenced Regressions On Watershed attributes (SPARROW) models to help describe the distribution, sources, and transport of nutrients in streams throughout much of the United States. After screening, 2,739 sites, sampled by 73 agencies, were identified as having suitable data for calculating long-term mean annual nutrient loads required for SPARROW model calibration. These sites had a wide range in nutrient concentrations, loads, and yields, and environmental characteristics in their basins. An analysis of the accuracy in load estimates relative to site attributes indicated that accuracy in loads improve with increases in the number of observations, the proportion of uncensored data, and the variability in flow on observation days, whereas accuracy declines with increases in the root mean square error of the water-quality model, the flow-bias ratio, the number of days between samples, the variability in daily streamflow for the prediction period, and if the load estimate has been detrended. Based on compiled data, all areas of the country had recent declines in the number of sites with sufficient water-quality data to compute accurate annual loads and support regional modeling analyses. These declines were caused by decreases in the number of sites being sampled and data not being entered in readily accessible databases.

## Introduction

Elevated concentrations of nutrients have been a persistent problem in streams and rivers throughout the United States (U.S.) (Robertson *et al.*, 2008). Such elevated concentrations can result in locally high algal, phytoplankton, and macrophyte biomass that have negative impacts on aquatic habitat and biota. Additionally, the transport of these nutrients can lead to degradation of downstream water bodies, such as lakes, reservoirs, and estuaries (USEPA, 2000). Noteworthy examples of problems associated with elevated nutrient concentrations vary from eutrophication of lakes and reservoirs to hypoxia (low concentrations or absence of dissolved oxygen) in the Gulf of Mexico. Eutrophication of many inland lakes (including the Great Lakes) has been linked to excessive phosphorus input, whereas hypoxia in the Gulf has been primarily linked to excessive nitrogen delivered from the Mississippi River Basin (Alexander *et al.*, 2008). To reduce problems associated with eutrophication, target loads (amount of a constituent delivered per unit time) for nutrient constituents have been established for many water bodies as part of management efforts known as total maximum daily loads. Such target loads have been established for the Gulf of Mexico by the Mississippi River/Gulf of Mexico Watershed Nutrient Task Force (2008) and the Great Lakes by the International Joint Commission as part of the Great Lakes Water Quality Agreement. Information describing nutrient loads over a large range of scales and geographic areas, as well as an understanding of the sources and transport of nutrients, are necessary to achieve these goals. Monitored nutrient loads in streams provide some of the necessary information; however, water-quality models provide a means to extrapolate this information to unmonitored areas.

**FIGURE 1:**
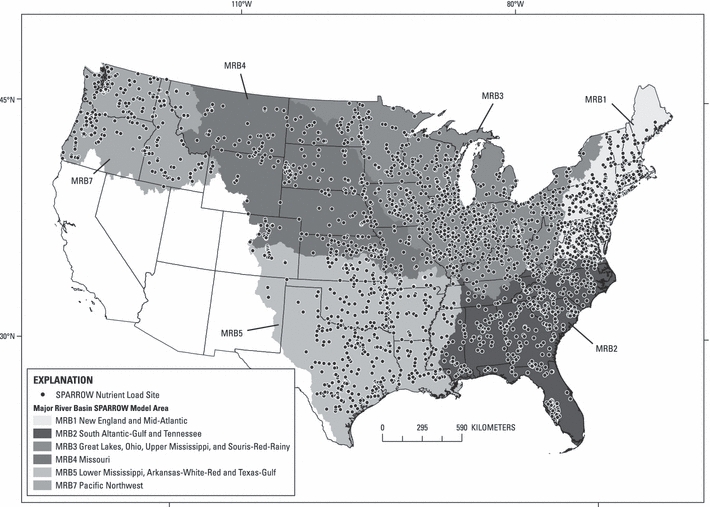
Map Showing Location of Load Sites Included in Regional Nutrient SPAtially Referenced Regressions On Watershed Attributes (SPARROW) Models.

Twelve SPAtially Referenced Regressions On Watershed attributes (SPARROW) models (Smith *et al.*, 1997) were recently developed for six Major River Basins (MRBs) (Figure 1) by the U.S. Geological Survey (USGS) to help scientists and managers understand the distribution, sources, and transport of nitrogen and phosphorus in streams and rivers for a large part of the U.S. (Hoos and McMahon, 2009; Brown *et al.*, this issue; Garcia *et al.*, this issue; Moore *et al.*, this issue; Rebich *et al.*, this issue; Robertson and Saad, this issue; Wise and Johnson, this issue). A large set of load monitoring sites, representing a wide range of watershed characteristics, was required to develop these regional models and reduce uncertainty in the estimated model coefficients (associated with important nutrient sources and watershed characteristics) and thereby improve prediction accuracy (Schwarz *et al.*, 2006; Preston *et al.*, 2009). The estimation of mean annual stream nutrient load, which represents the dependent variable in the SPARROW nutrient models, requires extended periods of coincident constituent concentration and flow data. In this paper, we describe the detailed process of assembling, processing, and assuring the quality of the water-quality and streamflow data used to compute the mean loads used in all 12 of these models. Characteristics of the sampled and modeled watersheds for the water-quality and flow data used in the models, and an analysis of the factors that affect the accuracy of load estimates, are also described. The results of the analysis of this dataset may also be useful to agencies conducting hydrologic monitoring to better understand the adequacy of current water-quality monitoring networks and ways in which current monitoring networks can be improved and possibly utilized beyond their original objectives.

## Description of Water-Quality and Streamflow Data, and Data Screening

The dependent variable in SPARROW models is long-term mean annual constituent load normalized to a base year (Preston *et al.*, 2009). The base year for the regional nutrient SPARROW models described here is 2002, which was selected so that calculated loads would coincide with the most recently available geospatial datasets of nutrient sources and environmental characteristics. Mean annual loads were estimated using data derived from water-quality samples and continuous measures of streamflow for each monitoring site. A description of the water-quality and streamflow data is given below. A detailed description of the load computation methods is included in the “Load Estimation” section of this paper.

The period for load estimation was chosen to include samples collected during the selected base year, as well as during adjacent years, to ensure that the relation between streamflow and water quality could be described and the resulting annual loads provide a representative estimate of mean hydrologic conditions. Generally, a longer period of record implies there may be more available data to calibrate the load-estimation models and results in a better estimate of the long-term load. However, too long a period can invalidate the estimate of the mean if conditions determining water-quality concentrations undergo significant change. For the regional SPARROW models, a sample period covering at least 1975-2005 was selected, the length of which is consistent with recommendations by Schwarz *et al.* (2006); however, a few regional models used a longer sample period. In accordance with the selected sample period, water-quality and streamflow data were compiled for sites from each region (a summary of site information, load data, and calculated loads is available in the Supporting Information, Table S1).

Flow data were compiled primarily from the USGS National Water Information System (NWIS; USGS, National Water Information System: Web Interface. http://waterdata.usgs.gov/nwis, *accessed* October 6, 2010), although some flow data were obtained from a few other sources, including the U.S. Army Corps of Engineers and the Colorado Division of Water Resources for streamgages in the Mississippi and Missouri River basins, and the Bureau of Reclamation, the Oregon Water Resources Department, and King County, Washington for streamgages in the northwestern U.S.

Water-quality data, nitrogen (N) and phosphorus (P), were compiled from federal, state, and local agencies and from selected universities. Most water-quality data were obtained from two databases: NWIS and the U.S. Environmental Protection Agency's (USEPAs) Storage and Retrieval (STORET) database (http://www.epa.gov/storet/index.html, *accessed* October 6, 2010). Water-quality data were retrieved from NWIS in July 2005. The USEPA provided snapshots of the Legacy (pre-1999) and Modernized (1999 and later) STORET databases in July 2005 (an updated snapshot for MRB3 was provided in April 2008). NWIS and STORET data were compiled into a single dataset by the USGS Center for Integrated Data Analytics (CIDA; http://cida.usgs.gov, *accessed* October 6, 2010) using an information model that defines common data elements and vocabularies for site types, constituents, and reporting units. Some of the protocols used to combine these data were defined in the recent federal water-quality data exchange collaboration between the USGS and USEPA (Scott *et al.*, 2008). Spatial and temporal gaps in the NWIS and STORET databases were supplemented, where possible, with data obtained from individual state and local agencies and selected universities. Data from agencies that collected ambient stream-water-quality data on a regular basis and were known or assumed to have standardized protocols for sample collection and laboratory analysis were included in this compilation.

The values used in load calculations were the concentrations of unfiltered total nitrogen as N (TN) and total phosphorus as P (TP), both expressed in units of milligrams per liter (mg/l). If a TN value was not available, it was estimated on the basis of data for particulate and dissolved (filtered) forms of measured N, if those data were available. Where total or particulate and dissolved measurements of N were unavailable, TN was calculated as the sum of the concentrations of ammonium, organic N, and nitrite plus nitrate (or just nitrate) where possible. The constituents considered when “creating” values for the concentrations of TN and TP, and the protocol for combining those concentrations, are described in the Supporting Information, Data S1.

Steps were taken to remove erroneous data in the datasets. Anomalously high or low concentrations were investigated and corrected when errors were fixable (such as concentration unit errors) or discarded if not readily fixable (such as input errors). Data with no specified location or missing sample dates were also excluded from the analyses.

**FIGURE 2:**
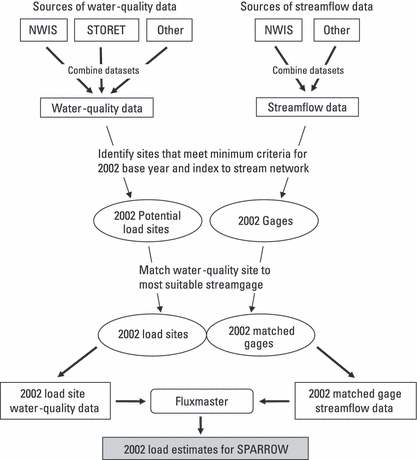
Flow Diagram Representing the Screening Process for Water-Quality and Flow Data Used to Calculate Load Estimates for Use in the Regional Nutrient SPAtially Referenced Regressions On Watershed Attributes (SPARROW) Models.

Once the water-quality datasets were compiled and checked, a screening process was performed to identify sites with sufficient data for computing the nutrient loads. Screening criteria included minimum requirements for availability of water-quality data, ability to locate the site on the SPARROW model stream network, and ability to associate the water-quality site with a suitable nearby streamgage. A flow diagram of the basic steps of the screening process is shown in Figure 2 and described below.

The accuracy of SPARROW model predictions is intrinsically linked to the accuracy of the water-quality monitoring station load estimate (the estimate of the mean annual TN or TP load). Smaller flux confidence limits often result from a larger number of water-quality observations (Schwarz *et al.*, 2006).

The water-quality criteria for selecting a site as a “potential load site” varied among MRBs, but generally, a site needed to have ≥20 TN or TP records and ≥2 years of water-quality data. Estimated loads were detrended to the 2002 base year (described in more detail in the “Load Estimation” section of this paper), so the length and proximity of the water-quality data to the base year were also considered in identifying potential load sites. A short period of record (less than five years) was expected to provide a less accurate estimate of trend in the water quality/streamflow relation than would a longer record; therefore, data were needed near the base year. A long period of record (≥5 years) was expected to provide a more accurate estimate of the trend in the water quality/streamflow relation, which could be extrapolated farther from the base year. Therefore, if the period of record was less than five years, a potential load site was required to have water-quality data within two years of the SPARROW model base year (2002); if the period of record was ≥5 or more years, it had to have water-quality data within seven years of the base year. For some MRBs with many potential load sites (such as MRB3), the minimum number of TN or TP records was increased to 25 and/or included the requirement that each season (spring, summer, fall, and winter) be represented by at least one measured concentration. This reduced the number of potential load sites for those areas (while still providing a high density of sites), but was expected to provide better load estimates. For MRB2, the minimum water-quality screening criteria included sites with quarterly sampling for ≥5 years during 1995-2005. Overall, the average duration between water-quality observations for all sites was 36 days (approximately monthly). In all cases, these minimum criteria for including water-quality records greatly reduced the number sites being considered as potential load sites (Table 1).

**TABLE 1:** Counts of Stream-Water-Quality Sites Throughout the Screening Process and Model Site Data Density for the Regional Nutrient SPARROW Models

	MRB1	MRB2	MRB3	MRB4	MRB5	MRB7
Sites with nutrient stream-water-quality data	20,913	21,591	33,118	11,366	22,012	15,524
Sites meeting minimum water-quality screening criteria	2,066	3,422	1,688	791	1,873	628
Sites included in SPARROW model^1^ (TN/TP)	363/457	321/370	708/810	193/311	344/442	178/228
MRB area (km^2^) (U.S. portion only)	443,815	821,591	1,371,536	1,323,281	1,384,481	718,410
SPARROW model site data density, sites/1,000 km^2^ (TN/TP)	0.84/1.09	0.39/0.47	0.51/0.59	0.15/0.24	0.25/0.32	0.25/0.32

Notes: MRB, Major River Basin; TN, total nitrogen; TP, total phosphorus; SPARROW, SPAtially Referenced Regressions On Watershed attributes.

1 Sites included in SPARROW model are the subset of sites meeting minimum water-quality screening criteria that were also indexed to the stream-reach network and matched to a streamgage.

Each potential load site was manually indexed to a digital stream network and matched to a streamflow gaging station if possible. Indexing of sites to the stream networks minimally required information on stream name and location. The stream network used for most MRB models was an enhanced version of the USEPA River Reach File 1 (RF1) (Nolan *et al.*, 2002; Brakebill *et al.*, this issue). However, the network used for MRB1 was an enhanced version of the USGS National Hydrography Dataset (NHD) stream network (USGS, 1999). All potential load sites were initially indexed to the NHD stream network to facilitate development of future SPARROW models that may use that network, and then matched to a corresponding location on the RF1 network. If the location of a potential load site could not be reasonably verified based on attributes such as latitude/longitude, stream name, and drainage area, the site was excluded from further consideration. Additionally, sites that could be indexed to NHD but not to the RF1 network (typically sites with very small drainage basins) were excluded from the RF1-based models.

Load calculations are ideally done using collocated water-quality and streamflow sites. Approximately 70% of the load sites in this dataset were collocated. Collocation was defined as being on the same river and drainage areas for water-quality and streamflow sites within 5%. Use of a nearby streamgage is a common approach when collocation is not possible; however, published information describing criteria for selecting a suitable streamgage is scarce. The criteria that were used for the noncollocated load sites of the regional nutrient SPARROW models are described here.

Matching a potential load site to a suitable streamgage involved identifying all nearby gages that met a minimum criterion of ≥2 years of daily flow data that included the base year (2002), and then selecting the gage with flow characteristics that best represented those at the water-quality monitoring location (described below). Suitable gages generally had to meet the following criteria:

1. An overlap between water-quality and flow data of ≥2 years.
2. The ratio of drainage areas between the water-quality site and the flow site of 0.5-2.
3. If the drainage area of the water-quality site was ≥260 km^2^ (100 mi^2^), then the flow gage must be on same stream.
4. If the drainage area of the water-quality site drainage area was <260 km^2^, then the gage could be on a nearby stream (only about 2% of selected gages were on a different stream, and most of those were in MRB3).
5. The gage must be within a reasonable distance of the water-quality site so as to represent similar environmental conditions. (The modelers in MRB3 used 40 km as their criteria for “reasonable.”)

Priority for selection was given to gages with a longer period of data overlap, drainage ratios closer to 1, and proximity to the water-quality site. Some of the streamgage criteria described above may be considered by some to be too relaxed for load calculation purposes, but the criteria were intended to provide a large number of suitable gages to choose from. For example, the drainage area ratio criteria (item #2 above) is slightly more relaxed than the guidelines (0.5-1.5) noted by Perry *et al.* (2004) for estimating streamflow statistics at ungaged sites. However, >97% of the sites used in the SPARROW models are within the 0.5-1.5 ratio range. In fact, >91% of the sites meet a much stricter 0.75-1.33 drainage area ratio. The effect of using noncollocated water-quality and streamflow sites on load estimates is evaluated later in this paper.

The initial compilation of nutrient data consisted of observations from approximately 125,000 stream sites, collected by 186 sampling agencies. Only 4-16% of those sites, however, met the minimum MRB water-quality screening criteria, and only 1-3% of sites had data considered adequate for calculating loads and being included in a MRB SPARROW model. The final dataset, reflecting all of the stated qualifications, is composed of water-quality observations from 2,739 sites; these data being compiled by 73 different agencies (Figure 1; Supporting Information, Tables S1 and S2). The number of load sites included in the individual MRB SPARROW models ranged from 178 (MRB7, for TN) to 810 (MRB3, for TP) (Table 1). The number of load sites was usually larger for TP than for TN. Site density for the models ranged from 0.15 sites per 1,000 km^2^ (MRB4, TN) to 1.09 sites per 1,000 km^2^ (MRB1, TP). The highest site densities were in MRBs 1, 2, and 3, and the lowest were in MRBs 4, 5, and 7.

## Basin Characteristics

**FIGURE 3:**
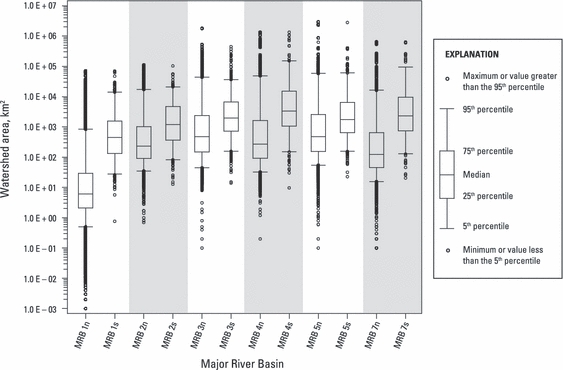
Graph Showing Distribution of Watershed Area, by Major River Basin, for Digital Stream-Reach Networks (n) and Final Load Sites (s) Included in Regional SPAtially Referenced Regressions On Watershed Attributes (SPARROW) Models. [Based on the RF1 stream network, except for MRB1, which is based on National Hydrography Dataset (NHD).]

For models with statistically estimated parameters, such as SPARROW, it is important to have a large number of monitoring sites that represent the most extreme combinations of environmental characteristics in the study area (Schwarz *et al.*, 2006). A select set of watershed characteristics were summarized for the final load sites and all of the watersheds in the digital stream-reach networks of the MRB SPARROW models (Table 2). Watershed area for final load sites ranged from <1 to >2,800,000 km^2^ (Table 2; Figure 3). The distribution of drainage sizes of watersheds of the final load sites did not represent all of those in digital stream-reach networks used in the MRB SPARROW models, the smaller watersheds were particularly under represented. Of greater importance for obtaining accurate model predictions, however, is that the range of digital stream-reach network sizes is nearly covered by the range of monitored watersheds. Nationally, the median watershed area of the final load sites was 1,516 km^2^; regionally, the median size ranged from 450 km^2^ (MRB1) to 3,204 km^2^ (MRB4). The median watershed area of the digital stream-reach networks used in the MRB SPARROW models was 12.9 km^2^ nationally, but ranged from 6 km^2^ (MRB1) to 478 km^2^ (MRB3) regionally. The larger percentage of small watersheds included in the MRB1 reach network than in the other MRBs was a result of the high resolution of the NHD data used for that network.

**TABLE 2:** Summary Statistics for Selected Environmental Characteristics of Monitored Watersheds *vs.* All Watersheds of the Digital Stream-Reach Networks Used in the Regional SPARROW Models

Watershed Characteristic^1^	Mean	SD	Minimum	25th Percentile	Median	75th Percentile	Maximum
Drainage area^2^ (km^2^)							
Monitored watersheds (*N* = 2,739)	13,104	78,220	<1	467	1,516	5,815	2,801,776
All watersheds (*N* = 240,588)	3,670	56,118	0.001	2.8	12.9	126	2,959,687
Mean annual streamflow (m^3^/s)							
Monitored watersheds	89	514	0.001	3.3	11.7	43.4	19,916
All watersheds (*N* = 2,144,926)	12	251	0	0.01	0.04	0.25	19,960
Mean annual precipitation (cm)							
Monitored watersheds	100.8	34.1	25.5	80.8	101.9	118.0	404.0
All watersheds	97.9	40.1	8.6	68.9	100.8	124.0	660.2
Mean air temperature (°C)							
Monitored watersheds	11.1	4.4	−1.3	7.8	10.6	13.8	22.6
All watersheds	11.2	4.8	−4.0	7.2	11.0	15.2	24.9
Land use/land cover (1992 NLCD)^3^							
Water (%)							
Monitored watersheds	1.3	1.9	0.0	0.3	0.7	1.4	27.0
All watersheds	1.4	5.7	0.0	0.0	0.1	0.7	100.0
Urban (%)							
Monitored watersheds	5.7	12.9	0.0	0.6	1.6	4.1	98.8
All watersheds	2.2	8.4	0.0	0.0	0.0	0.8	100.0
Barren (%)							
Monitored watersheds	1.2	2.5	0.0	0.1	0.3	1.2	34.8
All watersheds	1.5	5.4	0.0	0.0	0.0	0.5	100.0
Forest (%)							
Monitored watersheds	40.6	30.4	0.0	10.7	37.5	69.2	100.0
All watersheds	39.5	35.4	0.0	2.5	33.2	73.5	100.0
Rangeland (%)							
Monitored watersheds	11.2	21.4	0.0	0.0	0.1	8.2	99.4
All watersheds	19.0	32.0	0.0	0.0	0.1	25.3	100.0
Pasture/Hay (%)							
Monitored watersheds	15.1	13.9	0.0	4.4	11.2	21.1	77.5
All watersheds	13.6	17.3	0.0	0.3	6.2	20.8	100.0
Cropland/Orchard (%)							
Monitored watersheds	21.1	25.6	0.0	1.9	7.9	35.8	94.3
All watersheds	18.0	25.7	0.0	0.1	4.5	27.3	100.0
Wetland (%)							
Monitored watersheds	3.5	6.2	0.0	0.2	1.0	3.7	57.1
All watersheds	4.8	13.9	0.0	0.0	0.2	2.4	100.0

Notes: SPARROW, SPAtially Referenced Regressions On Watershed attributes; NLCD, National Land Cover Database; MRB, Major River Basin; NHD, National Hydrography Dataset.

1 Watershed characteristics, except for drainage area, based on accumulated attributes of the NHDPlus geodataset.

2 Drainage area based on the RF1 stream network, except for MRB1, which is based on NHD.

3 U.S. Geological Survey, National Land Cover Dataset 1992.

Locating the MRB monitoring sites on the NHD reach network permitted a comparison between network (all watersheds) and final load (monitored watersheds) sites for a number of watershed characteristics defined by the National Hydrography Dataset Plus (NHDPlus; Horizon Systems Corporation, 2009). The summary statistics for the comparison in Table 2 are based on data from all MRB study areas. A similar summary, by MRB, is included in the Supporting Information (Table S3). Attributes chosen for comparison included cumulative upstream drainage area, mean annual streamflow (based on a unit-runoff method), 1961-1990 mean annual precipitation, 1961-1990 mean air temperature, and 1992 land-use shares for various categories aggregated from the National Land Cover Database (NLCD) (USGS, National Land Cover Dataset 1992. http://landcover.usgs.gov/natllandcover.php, *accessed* October 6, 2010). NLCD classes were aggregated as follows: water (class 11), urban (classes 21, 22, 23, and 85), barren (classes 12, 31, 32, and 33), forest (classes 41, 42, and 43), range (classes 51 and 71), pasture (classes 81 and 84), crop (classes 61, 82, and 83), and wetland (classes 91 and 92). Generally, those attributes that do not vary greatly across large basins, such as precipitation and air temperature, had similarly distributed network and monitored watershed distributions. Conversely, attributes associated with scale, such as drainage area and streamflow, had different network and monitored watershed distributions. The land-use category percentages are not as easily characterized, with distributions being similar for forest and cropland/orchard and dissimilar for urban land, which was greatly overrepresented in the monitored watersheds, and rangeland, which was underrepresented. The range of values between the network and monitored watersheds was generally similar for all of the characteristics except the percent of barren land and wetland. There were few monitored watersheds with a high percentage of barren land or wetland.

## Load Estimation

Computation of detrended long-term mean annual loads for each final load site used in the MRB SPARROW models is based on the regression methods developed by Cohn (2005) and implemented in the program Fluxmaster (Schwarz *et al.*, 2006). Detrended mean annual loads provide an estimate of conditions normalized to a base year. The use of detrended mean annual loads in SPARROW models helps compensate for differences in the length and amount of monitoring data among sites, and minimizes the inherent noise introduced by year-to-year variations in rainfall facilitating the identification of environmental factors that affect loading over long periods (Preston *et al.*, 2009). The detrended load estimates are based on two models: a water-quality model and a flow model used to remove trends in streamflow. The water-quality model (Equation 1) relates the logarithm of concentration at time *t*, *c*_*t*_, to the logarithm of daily flow, *q*_*t*_, a decimal time term to represent trend, *T*_*t*_, sine and cosine functions of decimal time to account for seasonal variation, and a model residual, *e*_*t*_, 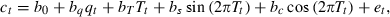
(1)where *b*_0_, *b*_*q*_, *b*_*T*_, *b*_*s*_, and *b*_*c*_ are fixed coefficients estimated for each site by the ordinary least squares method or, if some of the *c*_*t*_ measurements are censored, by the adjusted maximum likelihood method (Cohn, 2005), and *e*_*t*_ is assumed to be independent and normally distributed with mean 0 and variance 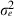.

Detrended flow is estimated using a flow model with the form 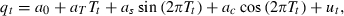
(2)where *a*_0_, *a*_*T*_, *a*_*s*_, and *a*_*c*_ are model parameters estimated using the maximum likelihood SAS Autoreg procedure (SAS Institute Inc., 2004; note: any use of trade, product, or firm names is for description purposes only and does not imply endorsement by the U.S. Government), and *u*_*t*_ is a model residual that is assumed to be correlated across time according to a 30-day lag autoregressive model. For some stations, a second-order harmonic of the sine and cosine functions was included and a 10-day lag autoregressive model was used. Final detrended flow, 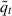, is then estimated using the relation 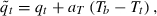
(3)where *T*_*b*_ is decimal time corresponding to June 30 of the designated base year (2002.5).

The logarithm of detrended daily concentrations are computed using Equation (1), with 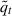 (detrended flow) and *T*_*b*_ (constant value for time) substituted for *q*_*t*_ and *T*_*t*_, and by adding 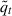 and an appropriate constant to obtain the logarithm of daily load. As Equation (1) is in logarithmic units, adding 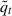 to Equation (1) is similar to multiplying the concentration by flow. These estimates are converted from logarithm space to real space using methods described by Cohn (2005) and Schwarz *et al.* (2006). The detrended long-term mean annual load is computed by identifying those years included in the analysis period for which there are no days with missing streamflow, summing the detrended daily load estimates for those days, and dividing by the number of included years to obtain mean load in units of kilograms per year.

The minimum number of uncensored measurements required to estimate a mean annual load was set at 15 for all MRBs except MRB7 and MRB3, which used 20 and 25, respectively. Only sites with calculated loads with a standard error (SE) <50% were considered for use in SPARROW models, although some loads with higher SEs were included if the estimated SE was suspect. A SE criterion of about 30% was used for the 1987 national nutrient SPARROW models (Schwarz *et al.*, 2006). Sites with small drainage basins used in the regional MRB models were expected to have large SEs, so the criterion was increased slightly. The stricter SE criterion of 30% was met by 96% of the TN sites and 84% of the TP sites.

## Concentrations, Loads, and Yields for Final Load Sites

**FIGURE 4:**
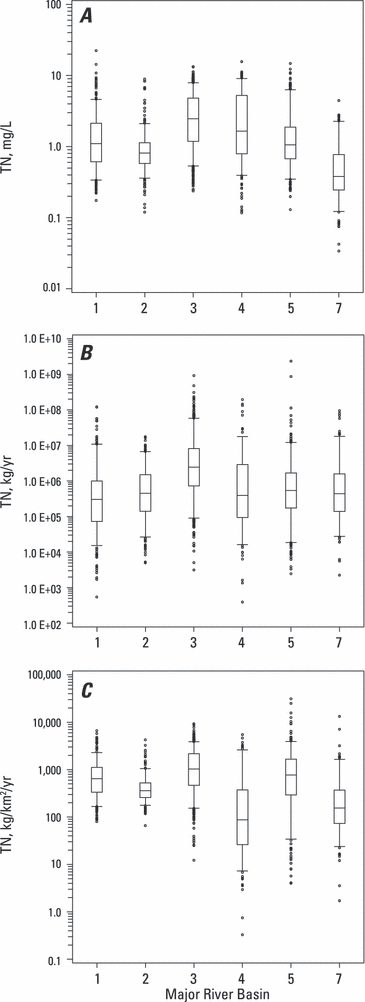
Graph Showing Distribution of Total Nitrogen (TN) Median Concentrations (A), Detrended Mean Annual Loads (B) and Yields (C) for Final Load Sites Used in SPAtially Referenced Regressions On Watershed Attributes (SPARROW) Models, by Major River Basin. (See Figure 3 for explanation of boxplot.)

**FIGURE 5:**
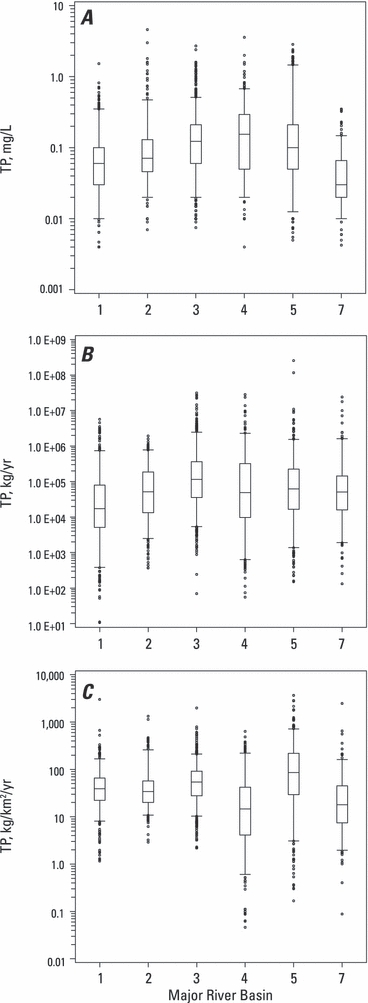
Graph Showing Distribution of Total Phosphorus (TP) Median Concentrations (A), Detrended Mean Annual Loads (B) and Yields (C) for Final Load Sites Used in SPAtially Referenced Regressions On Watershed Attributes (SPARROW) Models, by Major River Basin. (See Figure 3 for explanation of boxplot.)

Distributions of median TN and TP concentrations, detrended mean annual loads, and yields for all final load sites, by MRB, are presented as boxplots in Figures 4 and 5. Median concentrations for each site were calculated using a subset of the data used in load computations. The subset included only the samples collected closest to the middle of the month for sites at which more than one sample per month was collected. This was done to reduce possible bias associated with differences in the frequency of sampling among sites (such as weekly or sampling during storm events compared to the more common monthly or less than monthly sampling). Yields were calculated from the detrended loads divided by the station drainage areas, which were obtained from the stream-reach network used in the respective regional SPARROW models (RF1 or NHD).

Overall, median TN concentrations varied by more than two orders of magnitude, whereas median TP concentrations varied by nearly three orders of magnitude (Figures 4 and 5). Highest median TN concentrations were in MRB3 (Midwest), and highest median TP concentrations were in MRB4 (Missouri River), and lowest median TN and TP concentrations were in MRB7 (Northwest). Highest mean annual TN and TP loads were typically in MRB3 and lowest loads were in MRB1 (Northeast). After adjusting for the size of the watersheds, highest mean annual TN yields were still in MRB3; however, highest mean annual TP yields were in MRB5 (both draining to the Gulf of Mexico), and lowest mean annual TN and TP yields were in MRB4.

## Factors Affecting Accuracy in Load Estimates

An important consideration in using estimates of detrended mean annual load to develop a SPARROW model is the accuracy of the loads estimated from the approach described above. The accuracy of the load estimate has a direct bearing on both the coefficients of a SPARROW model and the accuracy of the SPARROW model predictions. Without knowing the relation between the load measurement error as it relates to basin characteristics, it is not possible to obtain a direct estimate of either the bias in the coefficients or the bias in the prediction errors. It can be shown (Schwarz *et al.*, 2006) that both biases are bounded by a factor that is proportional to the errors in the loads used for calibration. Therefore, understanding factors leading to errors in the mean load estimates is important for choosing stations to be used in calibration of the SPARROW models, thereby limiting the bias in the SPARROW analysis.

One of the objectives of this paper is to document the criteria that were used to obtain reliable estimates of load. A question of some interest is how important these criteria are for obtaining reliable load estimates. This information may be useful to future data compilation efforts and may help guide the design of water-quality monitoring strategies having the estimation of mean load as one of their objectives. As actual detrended mean annual load is never observed, it is not possible to directly assess the accuracy of the estimate. Cohn (2005) shows that if the assumptions of the regression method used to make the estimate are valid [i.e., Equations (1) and (2) are correctly specified for the full range of conditions experienced over the prediction period, with the residuals of Equation (1) being independent, normally distributed and having a common variance] then the estimated detrended mean annual load is unbiased in the case of no censoring and only second-order biased (i.e., as the number of measurements used to estimate the load model goes to infinity, bias goes to zero faster than the number of measurements goes to infinity) if there are censored water-quality measurements.

Using sequestered data techniques, Cohn *et al.* (1992) and Robertson and Roerish (1999) have shown that the regression method used in Fluxmaster applied to infrequent measurements of water quality provides reasonably unbiased estimates of daily load. Robertson and Roerish found the median bias to be <10% in annual TP loads if the streams are sampled on a routine basis such as used in most monitoring programs. Stenback *et al.* (2011) also found little if any bias in the estimation in TP loads, but did not examine biases in TN loads. As part of this study, we compared the predicted and measured loads (after the loads were transformed from log space to kilograms) for all days with measured concentrations for a uniform period from 1976 to 2004 for all MRBs. As continuous concentrations are not available, true annual loads could not be computed. For each site, the bias was computed as the average predicted daily load divided by the average actual daily load. We found the biases to be relatively small: median bias for TN loads was <1% and the median bias for TP loads was <8%. A more detailed description of this analysis is provided in the Supporting Information, Data S2.

In the absence of bias, the precision in the load estimates equates to the accuracy in the load estimates. In the following, we refer to the accuracy of the load estimate, with the understanding that this assessment is conditioned on the assumption that the model described by Equations (1) and (2) is valid and that, in the case of noncoincident water-quality and streamflow stations, the adjustment of streamflow is without error.

Here, we present a regression model which is used to describe how various factors in monitoring design are related to errors in detrended mean annual loads. A much more detailed description of the accuracy in the loads estimated with Fluxmaster and the statistical basis for this regression approach are provided in the Supporting Information, Data S2. The errors in loads were evaluated using the coefficient of variation (COV) from Fluxmaster. The COV is the ratio of the SE of the detrended mean annual load estimate divided by the estimate itself. The SE is equal to the square root of the summation of the variances of the loads across all days in the prediction period plus the summation of two times the covariance of the estimated loads between each individual day in this period, normalized by the number of years in the prediction (Gilroy *et al.*, 1990; this approach was extended to accommodate censored data by Cohn, 2005 and detrending of the load by Schwarz *et al.*, 2006). Two sites had extremely large TP COVs and were suspected to be the result of errors in the daily flows and were dropped from the analysis. The resulting analysis is based on mean load estimates from 2,107 stations for TN and 2,615 stations for TP. The median COV was 0.080 for TN and 0.133 for TP, the interquartile ranges were 0.051-0.130 for TN and 0.083-0.23 for TP, and the ranges were 0-1.78 for TN and 0.012-8.74 for TP. In agreement with the variability in nutrient concentrations, TP loads were estimated with less accuracy than TN loads.

Nine factors were examined to determine their importance to the accuracy in load estimation: root mean square of the concentration-discharge relation (Equation 1), number of observations, percent of uncensored observations, length of the period with observations, maximum days between observations (largest gaps), variability in flow on observation days (coverage of flow regimes), variability in flow on prediction days (stream flashiness), flow-bias ratio (representativeness of flows sampled), and a binary variable to indicate whether or not the load estimated was detrended. Results of the regression analyses for TN and TP are presented in Table 3. The COV of the mean detrended load is highly skewed and therefore, COV was logarithmically transformed. All continuous explanatory variables were also logarithmically transformed. This transformation implies that the coefficient for each variable represents the percent change in COV corresponding to a 1% change in each untransformed explanatory variable, holding constant the effects of the other variables.

**TABLE 3:** Summary of Regression Results for the Coefficient of Variation (COV) of the Log Load Estimate *vs.* Selected Station Attributes

	Log Total Nitrogen Load COV				Log Total Phosphorus Load COV			
		
Explantory Variable (station attribute)	Parameter Estimate	SE	*t*-Statistic	*p*-Value	Parameter Estimate	SE	*t*-Statistic	*p*-Value
Intercept	−0.210	0.125	−1.682	0.0926	1.302	0.120	10.811	**<0.0001**
Log RMSE of WQ model	0.643	0.010	63.049	**<0.0001**	1.266	0.023	54.588	**<0.0001**
Log number of WQ observations	−0.420	0.016	−25.685	**<0.0001**	−0.461	0.015	−30.067	**<0.0001**
Log percent of uncensored WQ observations	−0.623	0.064	−9.783	**<0.0001**	−0.327	0.039	−8.368	**<0.0001**
Log SD of flow for WQ observation days	−0.189	0.016	−12.033	**<0.0001**	−0.172	0.015	−11.834	**<0.0001**
Log period length of WQ observations	0.024	0.023	1.053	0.2922	−0.076	0.021	−3.660	**0.0003**
Absolute value of log of flow bias ratio	0.170	0.029	5.809	**<0.0001**	0.126	0.028	4.475	**<0.0001**
Log maximum days between WQ observations	0.014	0.008	1.644	0.1004	0.022	0.008	2.944	**0.0033**
Log SD daily flow for prediction period	0.160	0.017	9.404	**<0.0001**	0.151	0.016	9.603	**<0.0001**
If load estimate is detrended	0.229	0.019	12.209	**<0.0001**	0.305	0.017	17.630	**<0.0001**
Number of stations	2,107				2,615			
RMSE	0.369				0.380			
*R*^2^	0.785				0.748			

Notes: WQ, water-quality; RMSE, root mean squared error.

Significant explanatory variables (*p*-value <0.05) are highlighted in bold text.

The most important factor describing the error in the load estimates was the accuracy of the water-quality model as measured by the root mean squared error (RMSE) of Equation 1 (see the *t*-statistic in Table 3). The water-quality model RMSE was expected to be directly related to the overall accuracy of the load estimate. The results of this analysis strongly support this expectation, as the coefficients of both variables, for both TN and TP, had the expected signs and were highly significant. The reported coefficients for RMSE indicate that a 1% increase in the RMSE results in increases of 0.6 and 1.3% in the COV of the load estimates for TN and TP.

The number of observations and percent of uncensored observations were expected and found to be inversely related to the load COV. The coefficients for number of observations indicate that a 1% increase in *N* causes a 0.4% decrease in COV for TN and 0.5% decrease for TP. The coefficients for the percent of uncensored observations indicate that a 1% increase in the percent of uncensored observations causes a 0.6% decrease in COV for TN and a 0.3% decrease for TP.

Having samples collected over a wide range of flows and over a long time period were both expected to decrease the error in load estimates. The SD of streamflow for water-quality observation days is a close proxy for collecting samples over a wide range of flows. In both the TP and TN regressions, samples over a wide range in flows were associated with smaller COVs (negative coefficients). The length of the sample period had little relation (*p* > 0.29) with the accuracy in TN loads, but did appear to slightly improve the accuracy in TP loads.

Having samples that represent conditions over the entire prediction period was expected to decrease the error in load estimates. Two variables represent absolute differences in the prediction and sample period means. The flow-bias ratio describes the difference in streamflow between the prediction and sample periods. The magnitude of the coefficient for this absolute value of the log of the flow-bias ratio variable is interpreted as the percent change in COV from a 1% increase in mean streamflow for either the prediction period or the sample period, whichever value is larger. This variable was expected to and had a positive sign for both TN and TP reported, which strongly confirm this expectation. Based on these results, a 1% increase in the larger of the prediction or sample period streamflow means causes a 0.2% increase in COV for TN and a 0.1% increase for TP (Table 3).

The second variable is the maximum number of days between water-quality observations. The larger the maximum gap in the water-quality record, the greater the likelihood that one or more of the seasons is not representatively sampled and the means of the sine and cosine variables (Equation 2) in the water-quality samples are not zero. Therefore, larger gaps in the sample record were expected and found to have larger errors in the loads (although the estimate for TN was not statistically significant; the coefficient for TP was significant, but its magnitude was relatively small).

Increased variability in streamflow during the prediction period (increase in stream flashiness) was expected and found to increase the error in predicted loads. The magnitudes of the coefficients for this variable imply that a 1% increase in the SD of streamflow over the prediction period causes a 0.2% increase in both the TN and TP COVs.

The last variable examined was a dichotomous indicator of whether or not the data are sufficient to detrend the load estimate. In some cases the water-quality record was too short to give a valid estimate of trend or the water-quality record does not sufficiently cover the chosen normalization date for the detrended estimates. In these cases, a trend coefficient is not included in the water-quality model (Equation 1). The marginal effect of removing a variable from Equation 1, holding constant the RMSE of the model, was expected and found to increase the accuracy in the loads, that is, detrending increased the COV. Given a logarithm transformation applied to the dependent variable, 100 times the coefficient of a dichotomous variable is an approximate estimate of the percent change in the dependent variable. When holding RMSE fixed in the analysis, the inclusion of a trend variable causes COV to increase by approximately 23% for TN and 31% for TP.

The results of this regression model describing factors related to the error in detrended mean annual loads (Table 3) provides some insight into the relative importance of some of the criteria used to select stations for inclusion in a SPARROW model. Of obvious importance is the number of observations, especially uncensored values, available for calibration of the water-quality model (Equation 1). The number of observations has a quantitatively significant effect on the accuracy of the mean annual load estimate and selecting stations by this criterion is reasonable. Conversely, a criterion based on the length of the period with water quality samples has less relevance and should not be used alone. Of greater relevance is to collect samples over all the streamflow conditions experienced by the stream, that is, collect samples representing the mean and variance in streamflow that occurs throughout the entire prediction period. The results imply that water-quality samples that include large variability in streamflow, obtained by targeting specific flow events, but also selected to be representative of mean streamflow, produce more accurate estimates of mean load.

The most important factor in estimating loads was the accuracy in the calibration of the water-quality model itself (RMSE from Equation 1). It was believed that extrapolation of flow from nearby gages could affect the accuracy of Equation 1 and its resulting RMSE; therefore, we examined how the RMSE varied as the ratio of drainage areas between the water-quality site and the flow site deviated from 1. For collocated water-quality sites and streamflow gages (gages having drainage areas ±5% of the water-quality site), the average RMSE for the TN models was 0.40, and 0.64 for the TP models. For noncollocated sites (gages having drainage areas 0.5-2.0× the drainage area of the water-quality sites), the average RMSE was slightly lower; 0.39 for the TN models and 0.59 for the TP models, but tended to increase slightly for both models as the drainage ratio deviated from 1. The maximum value of a regression line through the data for noncollocated sites only approached the average RMSE values for collocated sites at drainage ratios of 0.5× and 2.0×. Therefore, the use of nearby gages on the same river (with drainage ratios from 0.5× to 2.0×) appears to have had little effect on the accuracy in the load estimates. For the small number of noncollocated sites on different rivers, the average RMSE values for TN and TP were higher than the collocated sites (0.47 for TN models and 0.70 for TP models), and the RMSE for these sites tended to increase as the drainage ratio deviated from 1, with maximum values of a regression through the data at 0.52 and 0.80 for TN and TP models, respectively. Therefore, the use of nearby gages on different rivers did appear to decrease the accuracy of the load estimates, but only by about 25% if the other constraints described earlier were followed.

## Temporal Trends in the Number of USGS Streamgages and Potential Nutrient Load Sites

**FIGURE 6:**
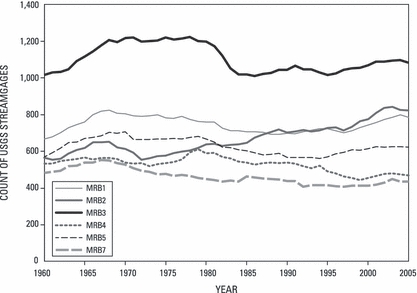
Graph Showing Yearly Count of U.S. Geological Survey (USGS) Streamgages (gages with minimum two years of daily flow record) by Major River Basin.

The regional MRB SPARROW models were constructed for a base year of 2002, using mean annual loads calculated for at least the period from 1975 to 2005, but the period of record represented by the assembled flow and water-quality data for each MRB often covered a longer period. This long period of record allowed us to evaluate trends in the number of flow and water-quality sites that could be considered for nutrient load calculations. Only USGS sites contained in NWIS were used to assess trends in the number of streamflow sites. Nationally, the number of USGS streamgages remained relatively stable since 1983 (USGS, 2008), but the trend in number of gages varied by MRB. A yearly count of the number of USGS streamgages with a minimum of two years of daily flow record (for sites with <5% missing record) is shown in Figure 6. In general, the number of gages meeting these criteria peaked around 1980, but has remained relatively steady since about 1985 for all MRBs except MRB2 and MRB4 (southeastern and south-central U.S., respectively). The number of gages in MRB2 continued to increase until about 2002; whereas, the number of gages in MRB4 has steadily decreased since 1980.

**FIGURE 7:**
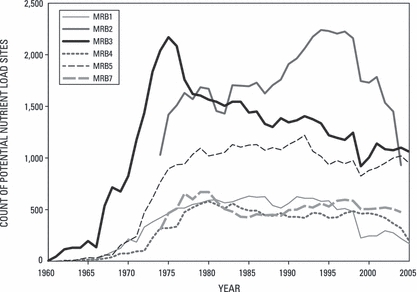
Graph Showing Yearly Count of Potential Nutrient Load Sites (sites with minimum of two years of record and 20 sampled days) by Major River Basin.

Trends in the number of potential nutrient load sites (≥2 years of record and ≥20 sampled days), as identified from the compiled datasets, also varied by MRB (Figure 7). All MRBs had an increasing number of potential load sites starting in the early to mid-1970s, but the number of sites generally decreased after the early- to mid-1990s. The timing of the peak number of sites ranged from the mid- to late-1970s (for MRBs 3, 4, and 7) to the early- to mid-1990s (for MRBs 2 and 5).

The types of changes in the number of potential load sites were quite variable among sampling agencies. For example, the number of potential load sites sampled by the USGS has declined since the mid-1970s in MRBs 2, 4, 5, and 7, but has remained relatively unchanged in MRBs 1 and 3 until the late 1990s. Changes in the number of potential load sites for some of the largest (by count) state agencies in each MRB are difficult to assess for the period after 1998 because many agencies stopped storing their data in STORET after the transition from Legacy to Modern STORET in 1999. Of the 186 agencies from which data were obtained for this study, 153 agencies had data suitable for at least one potential load site during the entire period of record. Access to readily available data from the 153 agencies (primarily from STORET) ended in 1998 or before for 61 agencies. Data for 15 agencies were available only for the years after 1998. Data from 66 agencies were available for at least five years before and after 1998. Eleven agencies had less than five years of data before and after 1998. The agencies whose data were no longer readily accessible starting around 1998 included some of the largest sampling agencies (by count) in the MRBs. For those agencies with ≥5 years of available data before and after 1998, most appear to have a decreasing (24 of 66) or similar (22 of 66) number of potential load sites after 1998. Only 20 of 66 agencies had an increasing number of potential load sites after 1998. In general, the overall downward trend in potential load sites after the mid-1990s in each MRB appears to be due to a combination of decreased monitoring and decreased data accessibility.

## Discussion

The monitoring sites used in the SPARROW MRB nutrient models represent the wide range of watershed conditions considered important for developing accurate models. The smallest drainage areas described by the reach network in the SPARROW models were underrepresented in the monitored basins, a finding that is understandable given the large number of small basins in the reach network of the models. The overrepresentation of highly urbanized land probably reflects the historical and continued interest in the effects of urban impacts on water quality. Relatively pristine areas (including barren land, wetland, and rangeland) may be underrepresented due to lack of pertinent water-quality issues in these areas; however, understanding background or natural conditions is important to consider when establishing water-quality criteria for streams and rivers (Robertson *et al.*, 2006) and understanding the effects of further anthropogenic changes.

The exclusion from consideration of many sites through the screening process, from approximately 125,000 to 2,739, indicates that, historically, much of the nutrient data collected were typically not intended for load estimation. The recent downward trends in the number of potential load sites indicates that without an increased commitment to long-term monitoring and data archiving, fewer sites will be available to support regional load modeling in the future. The process of constructing the multi-agency dataset used in the development of the regional SPARROW models yields important insights into improvements that could be implemented in the collection and compilation of water-quality data to support regional modeling efforts.

The main reasons that sites were excluded from consideration for use in SPARROW models were too few samples and/or too short of a sampling period. The minimum number of values required at a monitoring site to be considered in these regional nutrient models were 20 samples and two years of record. Secondarily, 50-90% of the sites meeting the minimum water-quality data requirements in a MRB were excluded because they could not be matched to a suitable streamgage (even though relatively relaxed selection criteria were used) or could not be accurately located on a stream reach (due to inadequate or missing location information).

As was also apparent from this effort, it was common for multiple agencies to collect samples at the same location. Sometimes this multi-agency effort increased the number of samples considered for analysis as well as the period of record, making a site suitable for load calculations, but sometimes samples were collected by different agencies within a relatively short period of time (sometimes on or close to the same day). Coordination of sample collection efforts and documentation could save time and money for all of the agencies involved. Standardizing readily available minimum location information (such as by indexing all water-quality sites to a common stream network such as NHD) could help maximize numbers of sites available for use in regional modeling efforts. This could minimize the effort required to accurately locate a site on a stream network and simplify the process of combining data from sites sampled by multiple agencies.

An obvious benefit of using multi-agency data is an increase in the number of sites available for regional models. The USGS has historically collected nutrient data at more stream locations in the areas examined in this study than any other sampling agency; however, the total number of sites available for estimating long-term loads would have been lower by two-thirds if only USGS data were considered in constructing the regional SPARROW models. A number of recent and currently planned efforts will significantly impact the ease with which multi-agency water-quality datasets can be compiled. The federal water-quality web service collaboration between the USGS and USEPA, through development of a common information model and tools, has allowed sampling agencies to more easily include their data in a national data-exchange network. States and Tribes can now contribute water-quality data to the STORET data warehouse via the Environmental Information Exchange Network through nodal access (http://www.exchangenetwork.net/index.htm, *accessed* October 6, 2010), and web services have become available that allow retrieval from both the STORET data warehouse and NWIS (National Water Information System Water-Quality Web Services. http://qwwebservices.usgs.gov/, *accessed* October 6, 2010). Additional tools are being developed to simplify the seamless integration of the STORET data warehouse and NWIS into a single dataset, moving further toward the goal of allowing users to access both systems via a single website from which portal software will query the independent systems and merge the output.

The number of actively used water-quality sites and streamgages continues to change. In the early 1970s, all MRBs had an increasing number of water-quality and streamgage sites, which resulted from actions taken as part of the 1972 Clean Water Act. Recently though, most MRBs have had a relatively stable number of streamgages, but a decrease in the number of potential nutrient load sites. The decrease in potential load sites is likely due to a combination of fewer sites being sampled and data not being input into readily accessible databases, such as STORET. If not addressed, both of these reasons could lead to less data for future regional water-quality modeling. For example, potential load sites from agencies that stopped putting data in STORET in 1998 may still meet the minimum criterion (data within seven years of the base year) for inclusion in the regional MRB SPARROW models with a base year of 2002. These sites, however, may not meet a similar criterion for inclusion in future models with base years after 2002.

Results from this study can help in designing future monitoring programs. Results from the regression analyses examining the principal factors affecting load-estimate accuracy (Table 3) can help to guide when and how often to collect water-quality samples. The results provide quantified estimates of the likely improvement in load accuracy resulting from increases in the number of water-quality observations, increases in the number of uncensored water-quality measurements, or increases in the range of flow being sampled. Results from the analyses of how RMSEs in load models change as the ratio of drainage areas between the water-quality site and the flow site changes help to guide how far gaging stations can be removed from the water-quality station and still provide accurate load estimations.

## Summary and Conclusions

A rigorous evaluation procedure was applied to reduce nearly 125,000 sites with available water quality to a select set of 2,739 sites for which water-quality and flow data were of sufficient quality and conformity to be suitable for long-term load estimation and inclusion in the calibration of regional MRB SPARROW models. This qualified set of water-quality and flow data represents a significant by-product of USGS MRB analyses; one that could provide considerable utility in other national, regional, or local water-quality assessments. The final load sites generally represented the wide range in watershed size and land-use characteristics in their respective areas. Highest median concentrations of TN were in the Upper Midwest (MRB3), and highest median TP concentrations were in the Missouri River Basin (MRB4); lowest median concentrations of TN and TP were in the Northwest (MRB7). Highest mean annual TN and TP loads were typically in the Upper Midwest, and lowest were in the Northeast. Highest mean annual TN yields were also observed in the Upper Midwest, whereas highest mean annual TP yields were in the south-central part of the U.S. (MRB5); lowest mean annual yields were in the Missouri River Basin.

Accuracy in TN and TP load estimates significantly improved with increases in the number of water-quality observations used to calibrate concentration-discharge regression models, such as developed within Fluxmaster, increases in the number of uncensored water-quality observations and increases in the variability in flow during which water-quality samples were collected. Accuracy in load estimates declined with increases in the maximum number of days between water-quality observations (largest gaps), and variability in daily streamflow (flashiness of flow in the stream).

In general, the number of streamgages has remained relatively stable since the early 1980s, except in the northeastern U.S., where the number has increased, and in the Missouri River Basin, where the number has decreased. Based on compiled data, all areas of the country represented in regional MRB SPARROW models have had a decrease in the number of sites with sufficient data to compute long-term mean annual nutrient loads. The recent decreases are likely due to a combination of a decreasing number of sites being sampled and the failure to enter the collected data into readily accessible databases (such as STORET) for sites that continue to be sampled. The federal water-quality web service collaboration between the USGS and USEPA has developed tools that allow sampling agencies to easily include their data in a national data-exchange network and to simplify large-scale retrievals of data from the USGS and USEPA databases.

If the recent trends are accurate, and continue, data from fewer sites will be available to calculate loads using regression approaches and to support further development of regional load models. Agencies that have an interest in estimating nutrient (or contaminant) loads and water-quality modeling, or would like their data to be considered for use in water-quality modeling, can use the list of key points shown below (derived from the compilation and analysis of data used in the regional nutrient SPARROW models) to help meet the need for continued monitoring, possibly with minimal effort or cost. Key points to consider when long-term annual load estimates and/or water-quality modeling are needed include the following:

1. Consider implementing sampling strategies that meet the needs of the agency while providing water-quality and flow data suitable for accurate load estimation. The latter goal could be met by collecting more samples, particularly during high-flow events, and associating the sampling site with a nearby streamgage, preferably collocated, or nearby on the same river.
2. Consider coordination of sampling efforts among agencies to maximize the use of limited resources, reduce redundancy, and increase interagency collaboration. Currently available data retrieval tools can be used by sampling agencies to identify locations where data are being or have been collected by other agencies.
3. Index stations to a national stream network, such as NHD, to provide useful and consistent location information. Detailed and accurate location information for monitoring stations facilitates the use of data by others.
4. Locate monitoring sites in underrepresented areas to cover a wide range of environmental variability and to improve the understanding of natural or background conditions.
5. Incorporate local and regional monitoring data in national databases (such as STORET) to potentially increase the utility of these data beyond their original objectives.

Water-quality models, such as the regional nutrient SPARROW models, have been, and will continue to be used by decision makers in the management of water resources. These models, and the decisions that water-quality managers make with them, can be improved in the future with the inclusion of more data suitable for load estimation.

## SUPPORTING INFORMATION

**Table S1.** Description of sites, load data, and calculated loads used in regional nutrient SPARROW models.

**Table S2.** Sampling agencies associated with nutrient water-quality data used in regional SPARROWmodels.

**Table S3.** Summary statistics, by Major River Basin, for selected environmental characteristics of monitored watersheds vs. all watersheds in the regional SPARROW model areas.

**Data S1.** Text file describing the constituents considered when creating TN and TP, and protocols for combining those constituents.

**Data S2.** Detailed description of the load estimates from Fluxmaster and factors affecting the accuracy in their estimations.

Please note: Neither AWRA nor Wiley-Blackwell is responsible for the content or functionality of any supporting materials supplied by the authors. Any queries (other than missing material) should be directed to the corresponding author for the article.
