# Supplementary material for: A Multi-Agency Nutrient Dataset Used to Estimate Loads, Improve Monitoring Design, and Calibrate Regional Nutrient SPARROW Models
Source: J Am Water Resour Assoc. 2011 Oct;47(5):933–49. doi: 10.1111/j.1752-1688.2011.00575.x (PMC3307626; doi:10.1111/j.1752-1688.2011.00575.x)
Supplement: Supplementary file 1 [file jawr0047-0933-SD1.pdf]

Supporting Information to:

A Multi-Agency Nutrient Dataset Used to Estimate Loads, Improve Monitoring Design, and Calibrate Regional Nutrient SPARROW Models by David A. Saad, Gregory E. Schwarz, Dale M. Robertson, and Nathaniel L. Booth (*this issue*).

Table S1. Description of sites, load data, and calculated loads used in regional nutrient SPARROW models.

Data S1. Text file describing the constituents considered when creating TN and TP, and protocols for combining those constituents.

Table S2. Sampling agencies associated with nutrient water-quality data used in regional SPARROW models.

Table S3. Summary statistics, by Major River Basin, for selected environmental characteristics of monitored watersheds versus all watersheds in the regional SPARROW model areas.

Data S2. Detailed description of the load estimates from Fluxmaster and factors affecting the accuracy in their estimations.

Table S1. Description of sites, load data, and calculated loads used in regional nutrient SPARROW models.

Major river basin identifier	tp	lf site	tn	lf site	Station identifier	Station_name	WQ data program	8-digit cataloging unit	Station latitude	Station longitude	NHDPlus reach identifier
1	1		0		01-AND	North Rd Bridge	11113300	01040002	44.3973	-70.9884	6712531
1	1		0		01-CNT	RTE 10 Bridge	11113300	01080201	42.7122	-72.4532	6778911
1	1		0		01-MER	RTE 113 Bridge, Mass	11113300	01070006	42.676	-71.4213	6746168
1	1		0		01-SAC	RTE 113 Bridge	11113300	01060002	44.0169	-70.9899	9311291
1	1		0		01-SGR	Bridge at RTE 12A and 12/103	11113300	01080106	43.3983	-72.3939	9329406
1	1		1		01036390	Penobscot River at Eddington, Maine	USGS	01020005	44.8266	-68.6966	1736862
1	1		1		01049265	Kennebec River at North Sidney, Maine	USGS	01030003	44.4723	-69.684	3325334
1	1		1		01054200	WILD RIVER AT GILEAD, ME	USGS	01040002	44.3906	-70.979	6716129
1	1		1		01059400	ANDROSCOGGIN RIVER AT BRUNSWICK, ME	USGS	01040002	43.9176	-69.9731	6713631
1	1		1		01064140	PRESUMPSCOT RIVER NEAR WEST FALMOUTH,	USGS	01060001	43.7246	-70.3029	6724855
1	1		1		01066000	Saco River at Cornish, Maine	USGS	01060002	43.808	-70.7815	9314337
1	1		1		01096550	MERRIMACK RIVER ABOVE LOWELL, MA	USGS	01070006	42.639	-71.3709	6746232
1	1		1		01100000	MERRIMACK RIVER BL CONCORD RIVER AT L	USGS	01070006	42.6459	-71.2984	6746184
1	1		1		01101500	IPSWICH RIVER AT SOUTH MIDDLETON, MA	USGS	01090001	42.5695	-71.027	5862629
1	1		0		01102500	Aberjona Rv at Winchester, MA	USGS	01090001	42.4473	-71.1389	5867393
1	1		1		01103500	CHARLES RIVER AT DOVER, MA	USGS	01090001	42.2562	-71.2601	5867435
1	1		1		01104615	Charles Rv abv Watertown Dam at Water	USGS	01090001	42.3648	-71.1898	5867403
1	0		1		01105000	NEPONSET RIVER AT NORWOOD, MA	USGS	01090001	42.1776	-71.2009	5867537
1	1		1		01109000	WADING RIVER NEAR NORTON, MA	USGS	01090004	41.9476	-71.1767	6129519
1	1		1		01111230	BLACKSTONE RIVER AT MILLVILLE, MA	USGS	01090003	42.0229	-71.5723	6116334
1	1		1		01111500	BRANCH RIVER AT FORESTDALE, RI	USGS	01090003	41.9965	-71.5626	6119204
1	1		1		01112900	BLACKSTONE RIVER AT MANVILLE, RI	USGS	01090003	41.9712	-71.4701	6118882
1	1		1		01116617	PAWTUXET RIVER AT PAWTUXET, RI	USGS	01090004	41.7676	-71.4053	6128891
1	1		1		01118500	PAWCATUCK RIVER AT WESTERLY, RI	USGS	01090005	41.3837	-71.8331	6140952
1	1		1		01119375	WILLIMANTIC R AT MERROW, CT	USGS	01100002	41.8354	-72.3101	6162555
1	1		1		01122610	SHETUCKET R AT SOUTH WINDHAM, CT	USGS	01100002	41.6823	-72.1659	6163199
1	1		1		01124000	QUINEBAUG RIVER AT QUINEBAUG, CT.	USGS	01100001	42.0223	-71.9556	6144833
1	1		1		01124151	QUINEBAUG R AT WEST THOMPSON, CONN.	USGS	01100001	41.9434	-71.9004	6148415
1	1		1		01125500	QUINEBAUG RIVER AT PUTNAM, CT	USGS	01100001	41.9093	-71.9129	6148535
1	1		1		01125520	QUINEBAUG RIVER AT COTTON BRIDGE ROAD	USGS	01100001	41.8584	-71.924	6148647
1	1		1		01127000	QUINEBAUG RIVER AT JEWETT CITY, CT.	USGS	01100001	41.5979	-71.9842	6149075
1	1		1		01134500	MOOSE RIVER AT VICTORY, VT	USGS	01080102	44.5117	-71.8373	4570675
1	1		1		01135500	PASSUMPSIC RIVER AT PASSUMPSIC, VT	USGS	01080102	44.3656	-72.0393	4572379
1	1		1		01138500	CONNECTICUT RIVER AT WELLS RIVER, VT	USGS	01080103	44.1534	-72.0418	9326224
1	1		1		01142500	AYERS BROOK AT RANDOLPH, VT	USGS	01080105	43.9345	-72.6579	6083547
1	1		1		01152500	SUGAR RIVER AT WEST CLAREMONT, NH	USGS	01080106	43.3876	-72.362	9329460
1	1		1		01154500	CONNECTICUT RIVER AT NORTH WALPOLE, N	USGS	01080107	43.1262	-72.4368	9331546
1	1		1		01163200	OTTER RIVER AT OTTER RIVER, MA	USGS	01080202	42.5884	-72.0409	9343543
1	1		1		01169000	NORTH RIVER AT SHATTUCKVILLE, MA	USGS	01080203	42.6384	-72.7251	10294784
1	0		1		01169900	SOUTH RIVER NEAR CONWAY, MA	USGS	01080203	42.542	-72.6937	10295340
1	1		1		01170100	GREEN RIVER NEAR COLRAIN, MA	USGS	01080203	42.7034	-72.6706	10294622
1	1		1		01171500	MILL RIVER AT NORTHAMPTON, MA	USGS	01080201	42.319	-72.6651	6781041
1	1		1		01184000	CONNECTICUT RIVER AT THOMPSONVILLE, C	USGS	01080205	41.9873	-72.6054	7700898
1	1		0		01184100	STONY BROOK NEAR WEST SUFFIELD, CT.	USGS	01080205	41.9607	-72.7104	7699976
1	1		1		01184490	BROAD BROOK AT BROAD BROOK, CT.	USGS	01080205	41.9139	-72.55	7700014
1	1		0		01186800	STILL R AT RIVERTON, CT	USGS	01080207	41.9595	-73.0196	6111165
1	1		1		01187800	NEPAUG R NR NEPAUG, CT.	USGS	01080207	41.8207	-72.9701	6109457
1	1		1		01188000	BURLINGTON BROOK NEAR BURLINGTON, CT.	USGS	01080207	41.7862	-72.9648	6109247
1	1		0		01188085	FARMINGTON R AT STATE HIGHWAY 4,AT UN	USGS	01080207	41.7645	-72.8959	6109641
1	1		1		01188090	FARMINGTON RIVER AT UNIONVILLE, CT.	USGS	01080207	41.7559	-72.8868	6109643
1	1		0		01189000	PEQUABUCK R AT FORESTVILLE, CT.	USGS	01080207	41.6732	-72.9007	6109479
1	1		1		01189030	PEQUABUCK R AT FARMINGTON, CT	USGS	01080207	41.7168	-72.8398	6109473
1	1		0		01189120	FARMINGTON R AT AVON, CT	USGS	01080207	41.8068	-72.8226	6109743
1	1		1		01189995	FARMINGTON RIVER AT TARIFFVILLE, CT.	USGS	01080207	41.9084	-72.7607	6109139
1	1		1		01192500	HOCKANUM RIVER NEAR EAST HARTFORD, CT	USGS	01080205	41.7832	-72.5873	7700148
1	1		1		01193500	SALMON RIVER NEAR EAST HAMPTON, CT.	USGS	01080205	41.5523	-72.4493	7700458
1	1		1		01196500	QUINNIPIAC RIVER AT WALLINGFORD, CT.	USGS	01100004	41.4495	-72.8409	6177036
1	1		1		01198125	HOUSATONIC RIVER NR ASHLEY FALLS, MA.	USGS	01100005	42.0748	-73.3334	7710044
1	1		0		01198135	HOUSATONIC RIVER NEAR SODOM, CT	USGS	01100005	42.0573	-73.3487	7710066
1	1		0		01199900	TENMILE RIVER AT SOUTH DOVER NR WINGD	USGS	01100005	41.6629	-73.5593	7712768
1	1		1		01200600	HOUSATONIC R NR NEW MILFORD, CT	USGS	01100005	41.5932	-73.4496	900510074
1	1		1		01205500	HOUSATONIC RIVER AT STEVENSON, CT.	USGS	01100005	41.384	-73.1676	7718288
1	1		1		01208049	NAUGATUCK RIVER NR WATERVILLE,CT.	USGS	01100005	41.6154	-73.0579	7713716
1	1		1		01208370	Naugatuck R below Fulling Mills Bk at	USGS	01100005	41.5018	-73.0482	7713308
1	1		1		01208500	NAUGATUCK RIVER AT BEACON FALLS, CT.	USGS	01100005	41.4423	-73.0626	7718060
1	1		0		01208873	ROOSTER RIVER AT FAIRFIELD, CT	USGS	01100006	41.1798	-73.219	7733285
1	1		0		01208950	SASCO BROOK NEAR SOUTHPORT, CT.	USGS	01100006	41.1529	-73.3059	7733309
1	1		0		01208960	SASCO BROOK AT SOUTHPORT, CT	USGS	01100006	41.1384	-73.2973	7733755
1	1		1		01208990	SAUGATUCK RIVER NEAR REDDING, CT.	USGS	01100006	41.2945	-73.3951	7731665
1	1		1		01209710	NORWALK RIVER AT WINNIPAUK,CT.	USGS	01100006	41.1354	-73.4262	7732571
1	1		1		01302500	GLEN COVE CREEK AT GLEN COVE NY	USGS	02030201	40.8634	-73.6343	9509668
1	1		1		01303000	MILL NECK CREEK AT MILL NECK NY	USGS	02030201	40.8876	-73.5637	9509792
1	1		0		01303500	COLD SPRING BROOK AT COLD SPRING HARB	USGS	02030201	40.8573	-73.4635	9509194
1	1		1		01304000	NISSEQUOGUE RIVER NEAR SMITHTOWN NY	USGS	02030201	40.8495	-73.2243	9509670
1	1		1		01304500	PECONIC RIVER AT RIVERHEAD NY	USGS	02030202	40.9137	-72.6868	9496622
1	1		1		01305000	CARMANS RIVER AT YAPHANK NY	USGS	02030202	40.8304	-72.9062	9496458
1	1		0		01305500	SWAN RIVER AT EAST PATCHOGUE NY	USGS	02030202	40.767	-72.9937	9496468
1	1		1		01306499	CONNETQUOT RIVER NEAR NORTH GREAT RIV	USGS	02030202	40.7476	-73.1504	9499636
1	1		0		01308000	SAMPAWAMS CREEK AT BABYLON NY	USGS	02030202	40.7043	-73.314	9499680
1	1		1		01308500	CARLLS RIVER AT BABYLON NY	USGS	02030202	40.7087	-73.3285	9499682
1	1		1		01309500	MASSAPEQUA CREEK AT MASSAPEQUA NY	USGS	02030202	40.689	-73.4548	9499694
1	1		1		01309950	BELLMORE CREEK NEAR BELLMORE NY	USGS	02030202	40.6787	-73.5157	9499700
1	1		1		01310500	EAST MEADOW BROOK AT FREEPORT NY	USGS	02030202	40.6657	-73.5698	9499702
1	0		1		01311000	PINES BROOK AT MALVERNE NY	USGS	02030202	40.6665	-73.6593	9499704
1	1		0		01327755	HUDSON RIVER AT ROGERS ISLAND AT FORT	USGS	02020003	43.2645	-73.5907	22288379
1	1		0		01331095	HUDSON RIVER AT STILLWATER NY	USGS	02020003	42.9356	-73.6518	22290453
1	1		1		01334805	HOOSIC R. IN EAGLE BRIDGE @ RT.67 BR.	USGS	02020003	42.9514	-73.3911	22289895
1	1		1		01349950	BATAVIA KILL AT RED FALLS NEAR PRATTS	USGS	02020005	42.3084	-74.3899	3247582
1	1		1		01351450	SCHOHARIE CREEK AT ESPERANCE NY	USGS	02020005	42.7609	-74.2554	3247250
1	1		0		01351500	SCHOHARIE CREEK AT BURTONSVILLE NY	USGS	02020005	42.8001	-74.2629	3247040
1	1		1		01357500	MOHAWK RIVER AT COHOES NY	USGS	02020004	42.7854	-73.7076	22751957
1	1		1		01362198	ESOPUS CR AT SHANDAKEN NY	USGS	02020006	42.1165	-74.3885	6189624
1	1		1		01377000	HACKENSACK RIVER AT RIVERVALE NJ	USGS	02030103	40.9992	-73.9892	6248392
1	1		1		01380500	ROCKAWAY RIVER ABOVE RESERVOIR AT BOO	USGS	02030103	40.9028	-74.4097	6250646
1	1		1		01381200	ROCKAWAY RIVER AT PINE BROOK NJ	USGS	02030103	40.8581	-74.3478	6250706
1	1		1		01382000	PASSAIC R AT TWO BRIDGES NJ	USGS	02030103	40.8973	-74.2726	6251110
1	1		1		01382500	PEQUANNOCK R AT MACOPIN INTAKE DAM NJ	USGS	02030103	41.0181	-74.4017	6246362
1	1		0		01387000	WANAQUE RIVER AT WANAQUE NJ	USGS	02030103	41.0442	-74.2931	6246344
1	1		1		01388500	POMPTON RIVER AT POMPTON PLAINS NJ	USGS	02030103	40.9692	-74.2819	6249470
1	1		1		01388600	POMPTON R AT PACKANACK LAKE NJ	USGS	02030103	40.9433	-74.2794	6250622
1	1		1		01389005	PASSAIC R BELOW POMPTON R AT TWO BRID	USGS	02030103	40.8965	-74.269	6251122
1	1		0		01389110	PASSAIC R AT RT 46 AT SINGAC NJ	USGS	02030103	40.8922	-74.2658	6251144
1	1		1		01389500	PASSAIC RIVER AT LITTLE FALLS NJ	USGS	02030103	40.8847	-74.2261	6250676
1	1		1		01389880	PASSAIC R AT RT 46 AT ELMWOOD PARK NJ	USGS	02030103	40.8937	-74.129	6251098
1	1		0		01390500	SADDLE R AT RIDGEWOOD NJ	USGS	02030103	40.9848	-74.0913	6250616
1	1		0		01391200	SADDLE RIVER AT FAIR LAWN, NJ	USGS	02030103	40.9417	-74.0931	6249688
1	1		1		01391500	SADDLE RIVER AT LODI NJ	USGS	02030103	40.8904	-74.0804	6250666
1	1		1		01393450	ELIZABETH R AT URSINO LAKE AT ELIZABE	USGS	02030104	40.675	-74.2219	6261532
1	1		1		01394500	RAHWAY R NR SPRINGFIELD NJ	USGS	02030104	40.6865	-74.3118	6261526
1	1		1		01395000	RAHWAY RIVER AT RAHWAY NJ	USGS	02030104	40.6189	-74.2833	6261548
1	1		1		01396280	SB RARITAN R AT MIDDLE VALLEY NJ	USGS	02030105	40.7611	-74.8214	9513780
1	1		1		01396535	SB RARITAN R ARCH ST AT HIGH BRIDGE N	USGS	02030105	40.6636	-74.8975	9512992
1	1		1		01396550	SPRUCE RUN AT NEWPORT NJ	USGS	02030105	40.7248	-74.9091	9513784
1	1		1		01396588	SPRUCE RUN NR GLEN GARDNER NJ	USGS	02030105	40.6781	-74.9181	9512884
1	1		1		01396660	MULHOCKAWAY CREEK AT VAN SYCKEL NJ	USGS	02030105	40.6475	-74.9689	9512952
1	1		0		01396800	SPRUCE RUN AT CLINTON NJ	USGS	02030105	40.6392	-74.9158	9513994
1	1		1		01397000	SOUTH BR RARITAN R AT STANTON STATION	USGS	02030105	40.5725	-74.8694	9513836
1	1		1		01398000	NESHANIC RIVER AT REAVILLE NJ	USGS	02030105	40.4717	-74.8281	9515526
1	1		1		01399500	LAMINGTON (BLACK) RIVER NEAR POTTERSV	USGS	02030105	40.7276	-74.7302	9513794
1	1		1		01400000	NORTH BRANCH RARITAN RIVER NEAR RARIT	USGS	02030105	40.5707	-74.679	9513840
1	1		0		01400120	RARITAN R AT RARITAN NJ	USGS	02030105	40.5644	-74.6358	9513352
1	1		1		01400500	RARITAN RIVER AT MANVILLE NJ	USGS	02030105	40.5551	-74.5835	9513852
1	1		1		01401000	STONY BROOK AT PRINCETON NJ	USGS	02030105	40.3332	-74.6818	9515592
1	1		1		01402000	MILLSTONE R AT BLACKWELLS MILLS NJ	USGS	02030105	40.475	-74.5761	9514706
1	1		0		01402540	MILLSTONE RIVER AT WESTON NJ	USGS	02030105	40.5297	-74.5883	9517822
1	1		1		01403300	RARITAN R AT QUEENS BRIDGE AT BOUND B	USGS	02030105	40.5595	-74.5277	9514000
1	1		0		01403900	BOUND BK AT MIDDLESEX NJ	USGS	02030105	40.5851	-74.5077	9513328
1	1		0		01404100	RARITAN RIVER NR SOUTH BOUND BROOK NJ	USGS	02030105	40.5406	-74.5138	9514020
1	1		0		01405440	MANALAPAN BK AT BRIDGE ST AT SPOTSWOO	USGS	02030105	40.3906	-74.3903	26606911
1	1		0		01407705	SHARK RIVER NEAR NEPTUNE CITY NJ	USGS	02030104	40.1986	-74.07	6262928
1	1		1		01407760	JUMPING BROOK NEAR NEPTUNE CITY NJ	USGS	02030104	40.2037	-74.0657	6263388
1	1		1		01408000	MANASQUAN RIVER AT SQUANKUM NJ	USGS	02040301	40.1632	-74.1554	9442083
1	1		1		01408500	TOMS RIVER NEAR TOMS RIVER NJ	USGS	02040301	39.9864	-74.2233	9453743
1	1		1		01409500	BATSTO RIVER AT BATSTO NJ	USGS	02040301	39.6417	-74.6503	9454589
1	1		1		01409815	WB WADING RIVER AT MAXWELL NJ	USGS	02040301	39.675	-74.5408	9447787
1	1		1		01410000	OSWEGO RIVER AT HARRISVILLE NJ	USGS	02040301	39.6633	-74.5242	9454525
1	1		1		01410150	EAST BRANCH BASS RIVER NEAR NEW GRETN	USGS	02040301	39.6231	-74.4414	9454631
1	1		0		01410820	GREAT EGG HARBOR R NR BLUE ANCHOR NJ	USGS	02040302	39.6692	-74.9133	9433603
1	1		1		01411000	GREAT EGG HARBOR RIVER AT FOLSOM NJ	USGS	02040302	39.5951	-74.8513	9434145
1	1		0		01411300	TUCKAHOE RIVER AT HEAD OF RIVER NJ	USGS	02040302	39.3069	-74.8206	9436435
1	1		1		01411500	MAURICE RIVER AT NORMA NJ	USGS	02040206	39.4951	-75.0768	9485398
1	1		0		01411800	MAURICE R NR MILLVILLE NJ	USGS	02040206	39.4478	-75.0725	9484602
1	1		0		01412800	COHANSEY RIVER AT SEELEY NJ	USGS	02040206	39.4725	-75.2556	9484462
1	1		1		01429301	Dyberry Creek at Tanners Falls Near D	USGS	02040103	41.6532	-75.2817	2739472
1	1		0		0143400680	E BR NEVERSINK R NORTHEAST OF DENNING	USGS	02040104	41.967	-74.4479	4147950
1	1		0		01434021	W BR NEVERSINK R AT FALLS UPSTR OF HI	USGS	02040104	42.0112	-74.4135	4146586
1	1		0		01434025	BISCUIT BROOK AB PIGEON BROOK AT FROS	USGS	02040104	41.9954	-74.501	4147946
1	1		0		01435000	NEVERSINK RIVER NEAR CLARYVILLE NY	USGS	02040104	41.8901	-74.5899	4147956
1	1		1		01437500	NEVERSINK RIVER AT GODEFFROY NY	USGS	02040104	41.4412	-74.6015	4151216
1	1		1		01438500	Delaware River at Montague, NJ	USGS	02040104	41.3093	-74.7952	4151628
1	1		1		01440000	FLAT BROOK NEAR FLATBROOKVILLE NJ	USGS	02040104	41.1068	-74.9521	4151302
1	1		1		01443000	Delaware River at Portland, PA	USGS	02040105	40.924	-75.0957	2588303
1	1		1		01443500	PAULINS KILL AT BLAIRSTOWN NJ	USGS	02040105	40.979	-74.9538	2586771
1	0		1		01444800	Delaware River at Belvidere Bridge (c	USGS	02040105	40.8289	-75.085	2588375
1	1		1		01445500	PEQUEST RIVER AT PEQUEST NJ	USGS	02040105	40.8306	-74.9783	2586809
1	1		1		01446400	PEQUEST R AT BELVIDERE NJ	USGS	02040105	40.8292	-75.0786	2587861
1	1		1		01447000	Delaware River at Northampton St at E	USGS	02040105	40.6918	-75.2038	2588457
1	1		1		01451800	Jordan Creek near Schnecksville, PA	USGS	02040106	40.6618	-75.6269	4187713
1	1		1		01457400	MUSCONETCONG R AT RIEGELSVILLE NJ	USGS	02040105	40.5922	-75.1886	2588477
1	1		1		01457500	DELAWARE R AT RIEGELSVILLE NJ	USGS	02040105	40.5934	-75.1877	2588261
1	1		1		01461000	Delaware River at Lumberville, PA	USGS	02040105	40.4076	-75.0374	2591449
1	1		1		01462500	Delaware River at Washington Crossing	USGS	02040105	40.2953	-74.8689	2590249
1	1		1		01463500	TRENTON WFP	USGS	02040105	40.2214	-74.7789	2590277
1	1		1		01463620	ASSUNPINK CREEK NEAR CLARKSVILLE NJ	USGS	02040105	40.2697	-74.6719	2590117
1	1		1		01464000	ASSUNPINK CREEK AT TRENTON NJ	USGS	02040105	40.2243	-74.749	2590137
1	1		1		01464500	CROSSWICKS CREEK AT EXTONVILLE NJ	USGS	02040201	40.1372	-74.6	4481607
1	1		1		01464504	CROSSWICKS C AT GROVEVILLE RD AT GROV	USGS	02040201	40.1672	-74.6775	4481599
1	1		1		01466500	MCDONALDS BRANCH IN BYRNE STATE FORES	USGS	02040202	39.885	-74.5053	4492036
1	1		1		01467000	NORTH BRANCH RANCOCAS CREEK AT PEMBER	USGS	02040202	39.97	-74.6844	4491898
1	1		1		01467081	SOUTH BRANCH PENNSAUKEN CREEK AT CHER	USGS	02040202	39.9418	-75.001	4495672
1	1		0		01467140	COOPER R AT LAWNSIDE NJ	USGS	02040202	39.8706	-75.0161	4494832
1	1		1		01467150	COOPER RIVER AT HADDONFIELD NJ	USGS	02040202	39.9032	-75.0216	4495892
1	1		1		01470500	Schuylkill River at Berne, PA	USGS	02040203	40.5226	-75.9983	4779167
1	1		1		01470779	Tulpehocken Creek near Bernville, PA	USGS	02040203	40.4134	-76.1716	4783101
1	1		1		01472000	SCHUYLKILL RIVER	USGS	02040203	40.2418	-75.6513	4782441
1	1		1		01472157	FRENCH CRK-.25MI UPSTRM FR WILSON COR	USGS	02040203	40.1514	-75.6017	4782153
1	1		1		01473170	Valley Creek at Wilson Road near Vall	USGS	02040203	40.0815	-75.4566	4781877
1	1		1		01474500	Schuylkill River at Philadelphia, PA	USGS	02040203	39.9679	-75.1885	4784831
1	1		0		01475300	Darby Creek at Waterloo Mills near De	USGS	02040202	40.0226	-75.4219	4489072
1	1		0		01480950	East Branch Brandywine Creek at Wawas	USGS	02040205	39.9265	-75.648	4650714
1	1		1		01481500	BRANDYWINE, RD 279 BRIDGE, DU PONT EX	USGS	02040205	39.7703	-75.5792	4655440
1	1		0		01482500	SALEM RIVER AT WOODSTOWN NJ	USGS	02040206	39.6434	-75.3307	9480708
1	1		1		01485000	POCOMOKE RIVER NEAR WILLARDS, MD	USGS	02060009	38.3889	-75.3244	8401303
1	1		1		01485500	NASSAWANGO CREEK NEAR SNOW HILL, MD	USGS	02060009	38.2289	-75.4714	8401785
1	1		1		01487000	NANTICOKE RIVER NEAR BRIDGEVILLE, DE	USGS	02060008	38.7283	-75.5619	8392898
1	1		0		01488500	MARSHYHOPE CREEK NEAR ADAMSVILLE, DE	USGS	02060008	38.8497	-75.6731	8391164
1	1		1		01491000	CHOPTANK RIVER NEAR GREENSBORO, MD	USGS	02060005	38.9972	-75.7858	9407484
1	1		1		01493500	MORGAN CREEK NEAR KENNEDYVILLE, MD	USGS	02060002	39.28	-76.0146	4766828
1	1		1		01540500	Susquehanna River at Danville, PA	USGS	02050107	40.9581	-76.6191	2604977
1	1		1		01541000	West Branch Susquehanna River at Bowe	USGS	02050201	40.897	-78.677	4673267
1	1		1		01545600	YOUNG WOMANS CR NR RENOVO PA	USGS	02050203	41.3895	-77.6908	8134878
1	1		1		01547400	Bald Eagle Creek near Milesburg, PA	USGS	02050204	40.9753	-77.7428	8141516
1	1		1		01549700	Pine Creek bl L Pine Creek near Water	USGS	02050205	41.2737	-77.3241	8145248
1	0		1		01552500	Muncy Creek near Sonestown, PA	USGS	02050206	41.357	-76.5347	8151091
1	1		1		01567000	Juniata River at Newport, PA	USGS	02050304	40.4784	-77.1291	4699925
1	1		1		01567795	Letort Spring Run at Bonny Brook near	USGS	02050305	40.1775	-77.1861	4711188
1	1		1		01570000	Conodoguinet Creek near Hogestown, PA	USGS	02050305	40.2522	-77.0211	4712048
1	1		1		01570500	Susquehanna River at Harrisburg, PA	USGS	02050305	40.2548	-76.8861	4710000
1	1		1		01572025	Swatara Creek near Pine Grove, PA	USGS	02050305	40.5326	-76.4022	4707802
1	0		1		0157608335	Little Conestoga Creek, Site 3A, near	USGS	02050306	40.1465	-75.9266	4720203
1	1		1		01576085	Little Conestoga Creek near Churchtow	USGS	02050306	40.1448	-75.9886	4720201
1	1		1		01576787	Pequea Creek at Martic Forge, PA	USGS	02050306	39.9059	-76.3283	4724801
1	1		1		01578310	SUSQUEHANNA RIVER AT CONOWINGO, MD	USGS	02050306	39.6573	-76.175	4726595
1	1		1		01591000	PATUXENT RIVER NEAR UNITY, MD	USGS	02060006	39.2383	-77.0557	11907296
1	1		1		01594000	LITTLE PATUXENT RIVER AT SAVAGE, MD	USGS	02060006	39.1344	-76.8162	11905730
1	1		1		01594440	PATUXENT RIVER NEAR BOWIE, MD	USGS	02060006	38.9559	-76.6937	11907758
1	1		1		01594526	WESTERN BRANCH AT UPPER MARLBORO, MD	USGS	02060006	38.8142	-76.7487	11908364
1	1		1		01594670	HUNTING CREEK NEAR HUNTINGTOWN, MD	USGS	02060006	38.584	-76.6052	11908476
1	1		1		01594710	KILLPECK CREEK AT HUNTERSVILLE, MD	USGS	02060006	38.4771	-76.7352	11909430
1	1		0		01603000	NORTH BRANCH POTOMAC RIVER NEAR CUMBE	USGS	02070002	39.6218	-78.7734	14363552
1	0		1		01608000	SOUTH FORK SB POTOMAC RIVER NEAR MOOR	USGS	02070001	39.0116	-78.9573	8420098
1	1		1		01610155	Sideling Hill Creek	USGS	02070003	39.6495	-78.3438	8431622
1	1		1		01614500	CONOCOCHEAGUE CREEK	USGS	02070004	39.7081	-77.8333	5891232
1	1		1		01634000	NORTH FORK SHENANDOAH RIVER NEAR STRA	USGS	02070006	38.9775	-78.3362	8441253
1	1		1		01636500	SHENANDOAH RIVER AT MILLVILLE, WV	USGS	02070007	39.2824	-77.7896	8445070
1	1		1		01639000	MONOCACY RIVER AT BRIDGEPORT, MD	USGS	02070009	39.6788	-77.2345	8448416
1	1		1		01643020	MONOCACY RIV AT REICHS FORD BRIDGE NR	USGS	02070009	39.3879	-77.3775	8450266
1	1		1		01644000	GOOSE CREEK NEAR LEESBURG, VA	USGS	02070008	39.0196	-77.5775	4507266
1	1		1		01646580	POTOMAC RIVER AT CHAIN BRIDGE, AT WAS	USGS	02070010	38.9296	-77.1169	22340529
1	1		1		01649500	NORTH EAST BRANCH ANACOSTIA RIVER AT	USGS	02070010	38.9603	-76.926	22337975
1	1		1		01651000	ON ANACOSTIA RIVER AT BLANDESBURG MD.	USGS	02070010	38.9428	-76.9675	22337977
1	1		1		01658000	MATTAWOMAN CREEK NEAR POMONKEY, MD	USGS	02070011	38.5961	-77.056	4529063
1	1		1		01658500	SOUTH FORK QUANTICO CR NEAR INDEPENDE	USGS	02070011	38.5873	-77.4286	4529107
1	1		1		01659000	NORTH BRANCH CHOPAWAMSIC CREEK NEAR J	USGS	02070011	38.5662	-77.4297	4529217
1	1		1		01660500	BEAVERDAM RUN NEAR GARRISONVILLE, VA	USGS	02070011	38.5071	-77.4289	4529453
1	1		1		01661050	ST CLEMENT CREEK NEAR CLEMENTS, MD	USGS	02070011	38.3333	-76.725	4530917
1	1		1		01668000	RAPPAHANNOCK RIVER NEAR FREDERICKSBUR	USGS	02080104	38.3085	-77.5292	8476948
1	1		1		01673000	PAMUNKEY RIVER NEAR HANOVER, VA	USGS	02080106	37.7676	-77.3322	8506380
1	1		1		01674500	MATTAPONI RIVER NEAR BEULAHVILLE, VA	USGS	02080105	37.8879	-77.163	8494082
1	1		1		01677000	WARE CREEK NEAR TOANO, VA	USGS	02080107	37.4382	-76.7863	9896768
1	1		0		02-ASH	RTE 63 Bridge	11113300	01080201	42.7861	-72.4865	6778371
1	1		1		02035000	RT. 45 BRIDGE AT CARTERSVILLE	USGS	02080205	37.6703	-78.0869	8575903
1	1		1		02038850	HOLIDAY CR NR ANDERSONVILLE VA	USGS	02080207	37.4154	-78.6358	8611825
1	0		1		02041650	APPOMATTOX RIVER AT MATOACA, VA	USGS	02080207	37.2252	-77.4753	8608039
1	1		0		02042500	CHICKAHOMINY RIVER NEAR PROVIDENCE FO	USGS	02080206	37.4363	-77.0608	10067073
1	1		0		03-OSS	RTE 153 Bridge	11113300	01060002	43.7964	-71.0563	9315959
1	1		0		04282636	LITTLE OTTER CR AB MIDDLE BRK RD NR F	USGS	02010008	44.1989	-73.2121	22220369
1	1		1		04296000	BLACK RIVER AT COVENTRY, VT	USGS	01110000	44.8689	-72.2701	4599789
1	1		1		05-LMP	RTE 108	11113300	01060003	43.0821	-70.935	5845174
1	1		1		05-OYS	RTE 108 Bridge	11113300	01060003	43.1309	-70.9186	5845086
1	0		1		06 1021	CHENANGO R. IN NORWICH @ BR.BTW.E.& W	21NYDEC1	02050102	42.4725	-75.5456	9422705
1	1		0		06-NSH	RTE 111 Bridge	11113300	01070004	42.7128	-71.5486	6076147
1	1		1		102011	SHELLPOT CR AT US RT 13 BRDG (GOV PRI	21DELAWQ	02040205	39.7531	-75.5167	4651906
1	1		1		103041	RED CLAY CREEK, ROAD 258A IN ASHLAND	21DELAWQ	02040205	39.8022	-75.6622	4651124
1	1		0		103051	RED CLAY CREEK, ROAD 252 IN YORKLYN	21DELAWQ	02040205	39.8061	-75.6806	4651090
1	1		0		104031	BRANDYWINE, DU PONT STATION AT HAGLE	21DELAWQ	02040205	39.7889	-75.5756	4652094
1	1		0		104041	BRANDYWINE CREEK AT ROCKLAND BRIDGE	21DELAWQ	02040205	39.7969	-75.5753	4655358
1	1		1		104051	BRANDYWINE CREEK AT SMITH BRIDGE	21DELAWQ	02040205	39.8375	-75.5792	4655354
1	1		1		105011	WHITE CLAY CREEK, AT DE RT 7 BRIDGE,	21DELAWQ	02040205	39.7069	-75.6525	4651938
1	1		0		105021	WHITE CLAY CREEK AT DE RT 2 BRIDGE NE	21DELAWQ	02040205	39.6922	-75.7236	4651394
1	1		0		105031	WHITE CLAY CREEK AT ROAD 329 NEAR THO	21DELAWQ	02040205	39.7328	-75.7603	4651292
1	1		1		106031	CHRISTINA RIVER AT SMALLEYS DAM SPILL	21DELAWQ	02040205	39.655	-75.6694	4651950
1	1		0		106111	CHRISTINA RIVER AT RD 346 BRIDGE	21DELAWQ	02040205	39.6381	-75.6811	4651470
1	1		1		106141	CHRISTINA RIVER, ROAD 364 AT CHESTNUT	21DELAWQ	02040205	39.6536	-75.755	4651956
1	0		1		11010215	HUDSON RIVER AT CORINTH NY	21NYDECA	02020003	43.2487	-73.8321	22288397
1	0		1		11021037	HOOSIC R. IN PETERSBURG @ RT.346 BR.	21NYDECA	02020003	42.8092	-73.2864	22290349
1	1		1		12010049	MOHAWK R. IN FONDA @ RT.30A BR.	21NYDECA	02020004	42.9503	-74.3697	22745895
1	1		1		14010001	Delaware River at Port Jervis, NY	21NYDECA	02040104	41.3706	-74.6974	4151524
1	1		0		14021001	DELAWARE RIVER	21NYDECA	02040104	41.3613	-74.6861	4151516
1	0		1		14021008	NEVERSINK R. IN GODEFFROY @ GRAHAM RD	21NYDECA	02040104	41.4381	-74.6017	4150304
1	1		0		16-MER	RR tressel near Rt. 3 Bridge	11113300	01070006	43.0955	-71.4645	6742074
1	1		1		1610400	Waites Run Near Wardensville, VA		02070003	39.0427	-78.5983	8433400
1	1		1		1621050	Muddy Creek at Mount Clinton, VA		02070005	38.4865	-78.9609	5908085
1	1		0		1904000365	NB RARITAN R AT NORTH BRANCH NJ	21NJDEP1	02030105	40.6	-74.6742	9513148
1	1		1		1AABR000.78	APPROX 0.2 MILES ABOVE RT.7 BRIDGE	21VASWCB	02070004	39.1792	-78.0861	5895952
1	1		1		1AACO006.10	RT. 790	21VASWCB	02070010	38.7286	-77.2033	22339471
1	0		1		1AAUA014.51	RT. 641 BRIDGE	21VASWCB	02070011	38.4908	-77.4344	4533657
1	1		1		1ACAX004.57	ROUTE 663	21VASWCB	02070008	39.255	-77.5767	4505612
1	0		1		1ACER006.00	ROUTE 646 BRIDGE	21VASWCB	02070010	38.6414	-77.5122	22339917
1	1		1		1ACER016.46	RT. 806 BRIDGE	21VASWCB	02070010	38.6367	-77.6261	22339947
1	1		1		1ADIF000.86	ROUTE 193 BRIDGE	21VASWCB	02070008	38.9758	-77.2461	4509202
1	1		0		1AFOU000.19	GEORGE WASHIGTON PARKWAY BRIDGE	21VASWCB	02070010	38.8411	-77.0483	22338859
1	1		1		1AGOO002.38	ROUTE #7 BRIDGE	21VASWCB	02070008	39.0856	-77.5114	4507238
1	1		1		1AGOO022.44	RT. 734 BRIDGE	21VASWCB	02070008	39.0133	-77.7003	4506950
1	1		1		1BCDR013.29	ROUTE 628 BRIDGE	21VASWCB	02070006	39.0778	-78.3261	8440247

Table S1. Description of sites, load data, and calculated loads used in regional nutrient SPARROW models.

Major river basin identifier	If tp site	If tn site	Station identifier	Station_name	WQ data program	8-digit cataloging unit	Station latitude	Station longitude	NHDPlus reach identifier	ERF1_2 reach identifier	Major river basin reach identifier	Station upstream area (km2)	DEM-estimated drainage area (km2)	RF1 estimate of mean flow (ft3/s)	Flow station identifier	Daily flow record start date for prediction'Flow station upstream area (km2)	Daily flow record start date for prediction	Daily flow record end date for prediction	Mean daily flow (ft3/s)	Standard deviation of daily flow (ft3/s)	No. days with valid flow data for prediction	Daily flow record start date for estimation	Daily flow record end date for estimation	If flow stats detrended to base year	Mean detrended daily flow (ft3/s)	Std. dev. detrended daily flow (ft3/s)	TN record start date	TN record end date	Number of TN observations	Number of censored TN observations	Median days between TN observations	Max. days between TN observations	Mean flow for TN observ. days (ft3/s)	Std. dev. flow for TN obs. days (ft3/s)	TN model root mean square error	TN model estimation method code	TN mean annual load (kg/yr)	Std. error TN mean annual load (kg/yr)	Std. dev. TN daily load (kg/yr)	TN nested area	TN model least squares weight	LN TN mean annual load	LN TN predicted mean annual load	LN TN residual	TP record start date	TP record end date	Number of TP observations	Number of censored TP observations	Median days between TP observations	Max. days between TP observations	Mean flow for TP observ. days (ft3/s)	Std. dev. flow for TP obs. days (ft3/s)	TP model root mean square error	TP model estimation method code	TP mean annual load (kg/yr)	Std. error TP mean annual load (kg/yr)	Std. dev. TP daily load (kg/yr)	TP nested area	TP model least squares weight	LN TP mean annual load	LN TP predicted mean annual load	LN TP residual
1	1	1	1BLNV000.21	DOWNSTREAM OF RT. 257 BRIDGE	21VASWCB	02070006	38.6181	-78.7986	8441047	3758		119.242	119.242	40.88471	01632082	119.242	10/1/1985	9/30/2005	39.63635866	99.82425598	7305	8/9/1985	9/30/2005	1	41.19378615	103.2368743	9/23/1985	12/10/1998	142	0	30	118	48.90211268	89.11363967	0.485301586	2.1	248090.7909	22991.9767	3706.984719	119.242	1	12.42155005	12.47623784	-0.054687789	9/23/1985	12/10/1998	142	44	30	118	48.90211268	89.11363967	0.742646625	2.1	4739.042738	921.1335467	116.421856	119.242	1	8.463590432	9.572623127	-1.109032695
1	1	1	1BNFS000.57	APPROX. 0.1 MILE BELOW RT. 340/522 BR	21VASWCB	02070006	38.9494	-78.1983	8441255	3735		2680.425	2680.425	919.04184	01634000	2003.487	10/1/1975	9/30/2005	894.9138275	1679.474356	10958	10/1/1975	9/30/2005	1	943.8754168	1767.916446	11/17/1975	12/1/1998	231	0	31	246	898.7077958	1340.51478	0.45158178	2.1	1785164.096	110930.5949	16778.20457	165.263	1	14.3950209	14.49637857	-0.101357665	3/1/1979	12/1/1998	202	103	31	246	913.7984687	1356.593223	0.430604114	2.1	91658.65863	7028.975722	643.8461723	165.263	1	11.42582672	12.05895719	-0.633130466
1	1	1	1BNFS029.69	RT. 600 BRIDGE (SHENANDOAH COUNTY)	21VASWCB	02070006	38.9094	-78.4264	8440817	3740		1861.726	1861.726	638.33313	01634000	2003.487	10/1/1975	9/30/2005	621.5746907	1166.501982	10958	10/1/1975	9/30/2005	1	655.5816351	1227.93065	7/16/1991	6/6/1995	47	0	29	59	612.8850973	1055.767974	0.248652217	2.1	1547743.913	114514.2618	10150.72271	474.98	1	14.25230889	14.14439445	0.107914432	7/16/1991	6/6/1995	47	14	29	59	612.8850973	1055.767974	0.474688791	2.1	82898.4736	13159.47333	750.3312972	474.98	1	11.32537193	11.14067705	0.184694875
1	1	1	1BNFS070.67	ROUTE 698 BRIDGE	21VASWCB	02070006	38.7456	-78.6392	8441291	3748		1386.746	1386.746	475.47594	01633000	1386.746	10/1/1975	9/30/2005	442.1033126	950.172525	10958	10/1/1975	9/30/2005	1	442.0610017	950.6209954	4/23/1979	12/7/1998	165	0	31	1202	523.3393939	875.411816	0.300752252	2.1	1054510.367	42558.87626	7248.931181	279.677	1	13.86858711	14.04839074	-0.179803631	4/23/1979	12/7/1998	165	65	31	1202	523.3393939	875.411816	0.543342083	2.1	47708.37473	4597.313452	398.8159386	279.677	1	10.77286223	10.86904467	-0.096182434
1	1	1	1BNFS081.42	RT. 617/953 BRIDGE, W OF NEW MARKET	21VASWCB	02070006	38.6521	-78.698	8441309	3749		857.861	857.861	294.13625	01632000	547.295	10/1/1975	9/30/2005	331.6534313	948.1909165	10958	10/1/1975	9/30/2005	1	383.3124324	1104.682897	11/11/1975	12/7/1998	230	0	31	123	363.0299685	828.0964121	0.296877077	2.1	841651.0371	30649.15077	6241.939686	191.324	1	13.64312077	13.84550617	-0.202385405	4/23/1979	12/7/1998	200	77	31	118	391.4643756	878.0454408	0.583195079	2.1	32499.78114	3640.353117	272.7362744	191.324	1	10.38898863	10.63338011	-0.24439148
1	1	1	1BNFS093.53	ROUTE 259 BRIDGE	21VASWCB	02070006	38.6372	-78.8525	8441037	3750		547.295	547.295	187.65196	01632000	547.295	10/1/1975	9/30/2005	211.5870341	604.9233473	10958	10/1/1975	9/30/2005	1	244.5442533	704.7615246	11/11/1975	12/7/1998	226	2	32	180	234.4090265	532.6133405	0.51008951	2.1	388697.5956	31254.64974	5655.795473	547.295	1	12.87055694	12.37844598	0.492110967	4/23/1979	12/7/1998	200	161	31	118	249.747	560.3579057	0.541757874	2.1	16348.94787	2404.160459	175.5262395	547.2951	1	9.701918826	9.514182485	0.187736341
1	1	1	1BNTH021.00	RIVER MILE AT WILDWOOD PARK DAM IN BR	21VASWCB	02070005	38.3842	-78.9886	5908707	3707		767.744	767.744	308.68183	01622000	973.69	10/1/1975	9/30/2005	332.7385355	604.1029763	10958	10/1/1975	9/30/2005	1	343.2088969	622.5564807	8/4/1988	12/21/1998	100	0	34	189	313.9133002	430.7285814	0.353753185	2.1	572727.3757	33897.95999	2637.765427	724.668	1	13.25816511	13.32784872	-0.069683616	8/4/1988	12/21/1998	101	66	33.5	189	311.8279416	429.0816478	0.329522835	2.1	28827.64946	2849.427195	174.8731477	724.668	1	10.26909026	10.22652611	0.042564151
1	1	1	1BPSG001.36	RT. 55 BRIDGE	21VASWCB	02070006	38.9589	-78.2667	8441257	3760		227.101	227.101	77.8665	01635500	227.101	10/1/1975	9/30/2005	81.41363387	186.6768183	10958	10/1/1975	9/30/2005	1	90.36178505	206.09274	10/9/1975	12/2/1998	218	47	33	284	85.84036697	171.399711	0.531562507	2.1	33843.92895	3141.117678	448.1798289	227.101	1	10.42951494	10.89884703	-0.469332087	4/11/1979	12/2/1998	192	157	30	284	88.5578125	178.5037666	0.347881187	2.1	6053.978069	752.2163874	40.52955508	227.101	1	8.708470872	8.155403393	0.553067479
1	1	1	1BSHN022.63	RT. 7 BRIDGE, CASTLEMANS FERRY BRIDGE	21VASWCB	02070007	39.1246	-77.8916	8445178	3764		7577.051	7577.051	2845.78274	01636500	7875.258	10/1/1975	9/30/2005	2887.612019	4403.262186	10958	10/1/1975	9/30/2005	1	3048.214097	4645.326717	10/14/1975	12/1/1998	218	0	32	246	2858.163755	3297.454356	0.30729464	2.1	4389518.614	160886.3261	25221.70522	371.118	1	15.29473012	15.34208483	-0.047354707	3/2/1979	12/1/1998	193	61	31.5	246	2900.349501	3318.670892	0.351749235	2.1	300907.7132	15699.69687	1535.13319	371.1188	1	12.6145589	12.65310459	-0.038545691
1	1	1	1BSHN048.00	RT. 624 BRIDGE	21VASWCB	02070007	38.9583	-78.1218	8446510	3764		7205.933	7205.933	2723.95332	01636500	7875.258	10/1/1975	9/30/2005	2746.179053	4187.593866	10958	10/1/1975	9/30/2005	1	2898.914967	4417.802267	10/14/1975	12/1/1998	130	0	31	3471	2879.913824	3180.702631	0.364657604	2.1	4346286.681	222910.1237	21509.80844	255.473	1	15.2848324	15.2698811	0.014951299	9/1/1988	12/1/1998	103	28	31	256	2949.616336	3166.133909	0.299264147	2.1	299606.3618	17418.26504	1606.635284	255.473	1	12.61022476	12.63622536	-0.026000592
1	1	1	1BSMT004.60	RT. 620 BRIDGE	21VASWCB	02070006	38.6939	-78.6433	8441303	3759		249.208	249.208	85.44637	01632900	249.208	10/1/1975	9/30/2005	80.15558496	156.5596476	10958	10/1/1975	9/30/2005	1	78.90220043	154.5530011	4/23/1979	12/7/1998	200	0	31	121	89.2495	146.9722772	0.288803651	2.1	203847.3197	7895.217272	1706.797729	249.208	1	12.22512656	12.67442164	-0.449295075	4/23/1979	12/7/1998	199	58	31	162	89.50201005	147.2994528	0.516386688	2.1	9476.200778	772.4915182	78.07423776	249.208	1	9.156538755	9.911453479	-0.754914723
1	1	1	1BSSF000.58	APPROX. 0.4 MILE BELOW RT340/522 BRID	21VASWCB	02070005	38.9433	-78.1928	5907065	3702		4270.035	4270.035	1716.82515	01631000	4235.232	10/1/1975	9/30/2005	1722.189779	2883.621848	10958	10/1/1975	9/30/2005	1	1763.330491	2951.686941	10/9/1975	9/22/2005	479	0	17	1000	2443.321435	6190.084856	0.361386934	2.3	2343829.799	77901.01978	16566.69756	960.122	1	14.66729682	14.96721142	-0.299914599	2/6/1979	9/22/2005	448	60	15	1000	2528.019852	6387.736561	0.548021738	2.3	206235.3068	12184.20669	1521.914578	960.1222	1	12.23677306	12.43268132	-0.195908257
1	1	1	1BSSF078.20	RI. MI. NEAR DAM ON S FRK SHEN. NEAR	21VASWCB	02070005	38.4825	-78.6278	5908617	3703		3309.913	3309.913	1330.79515	01629500	3561.877	10/1/1979	9/30/2005	1406.494629	2378.695126	9497	6/1/1979	9/30/2005	1	1365.006839	2311.090051	8/2/1988	12/7/1998	107	0	30.5	256	1560.489579	2093.40043	0.269433134	2.1	2506769.787	98229.5536	11952.15581	535.878	1	14.73450555	14.75119313	-0.016687579	8/2/1988	12/7/1998	106	12	31	256	1571.073338	2100.467075	0.441510262	2.1	202704.418	16110.52128	1272.080894	535.8784	1	12.21950413	12.07789747	0.141606658
1	1	1	1BSSF100.10	RT. 708 BRIDGE	21VASWCB	02070005	38.3128	-78.7703	5908729	3704		2774.035	2774.035	1115.33817	01628500	2806.073	10/1/1975	9/30/2005	1106.820639	1824.440965	10958	10/1/1975	9/30/2005	1	1097.817288	1810.800215	4/24/1979	12/21/1998	190	0	34	175	1161.927982	1768.038762	0.223270365	2.1	1976237.335	44297.64284	6755.776469	2006.291	1	14.49670526	15.03692345	-0.540218196	4/24/1979	12/21/1998	191	32	34	168	1159.534952	1763.69	0.528797629	2.1	138113.1161	8155.405965	502.6697305	1677.2525	1	11.83582831	12.16660549	-0.330777184
1	1	0	1BSTH027.85	ROUTE 664 BRIDGE - CITY OF WAYNESBORO	21VASWCB	02070005	38.0572	-78.9081	5908521	3728		329.039	329.039	132.29457	01626000	329.039	10/1/1975	9/30/2005	160.9146742	284.8687619	10958	10/1/1975	9/30/2005	1	160.0432139	283.5044381																			4/30/1979	12/15/1998	198	107	32	168	173.5555556	229.2981444	0.469185884	2.1	13348.97606	1162.773402	78.67377723	329.0392	1	9.499194964	9.522801484	-0.02360652
1	1	0	2-16-ASH	ASHUELOT RIVER	11113300	01080201	42.8867	-72.2883	6778119	1293		807.182	807.182	562.27261	01161000	1090.557	10/1/1975	9/30/2005	544.9255647	602.0111839	10958	10/1/1975	9/30/2005	1	544.6595318	601.6812828																			5/19/1976	9/13/2003	82	0	29	2807	334.992673	393.9781702	0.709753032	2.3	13246.46726	2910.554346	34.48094617	807.1825	1	9.491486177	9.733606423	-0.242120246
1	1	1	2-APP050.23	RT. 360 GOODES BRIDGE	21VASWCB	02080207	37.3548	-77.8518	8607197	4253		2327.558	2327.558	845.39519	02040000	1877.282	10/1/1975	9/30/2005	915.6467698	1361.232394	10958	10/1/1975	9/30/2005	1	834.1803529	1241.87911	7/1/1976	12/21/1998	224	2	31	206	987.8934399	1390.569144	0.377248884	2.1	545287.1951	24020.77941	3355.205315	893.661	1	13.20906791	13.30656495	-0.09749704	8/15/1979	12/21/1998	224	64	29	160	964.9063016	1368.796001	0.750313638	2.1	100754.9553	11663.09378	953.3458626	590.5011	1	11.52044666	11.03772138	0.482725282
[illegible]																																																															

Table S1. Description of sites, load data, and calculated loads used in regional nutrient SPARROW models.

Major river basin identifier	tp	If site	tn	If site	Station identifier	Station_name
2		1		1	02306774	ROCKY CREEK AT ST HWY 587 AT CITRUS PARK FL
2		1		1	02314500	SUWANNEE RIVER AT US 441, AT FARGO, GA
2		1		0	02317500	ALAPAHA RIVER AT STATENVILLE, GA
2		1		0	023177483	WITHLACOOCHEE RIVER AT MCMILLAN RD,NEAR
2		1		1	02318500	WITHLACOOCHEE RIVER AT US 84, NEAR QUITMA
2		1		0	02318940	WITHLACOOCHEE R (COUNTY RD) NR CLYATTVIL
2		1		0	02332017	CHATTAHOOCHEE RIVER BELTON BRIDGE ROAD
2		1		1	02334885	SUWANEE CREEK AT SUWANEE, GA
2		1		1	02336300	PEACHTREE CREEK AT ATLANTA, GA
2		1		0	02337000	SWEETWATER CREEK NEAR AUSTELL, GA
2		1		1	02337170	CHATTAHOOCHEE RIVER NEAR FAIRBURN, GA
2		1		1	02337500	SNAKE CREEK NEAR WHITESBURG, GA
2		1		1	02338000	CHATTAHOOCHEE RIVER NEAR WHITESBURG, GA
2		1		0	02338660	NEW RIVER AT GA 100, NEAR CORINTH, GA
2		1		1	02343801	CHATTAHOOCHEE RIVER NEAR COLUMBIA, AL
2		1		0	02344500	FLINT RIVER NEAR GRIFFIN, GA
2		1		0	02347500	FLINT RIVER NEAR CULLODEN, GA
2		1		1	02349500	FLINT RIVER AT MONTEZUMA, GA
2		1		1	02350080	LIME CREEK NEAR COBB, GA
2		1		0	02351890	MUCKALEE CREEK AT GA 195, NEAR LEESBURG, GA
2		1		1	02353000	FLINT RIVER AT NEWTON, GA
2		1		1	02353500	ICHAWAYNOCHAWAY CREEK AT MILFORD, GA
2		1		0	02357000	SPRING CREEK NEAR IRON CITY, GA
2		1		1	02392000	ETOWAH RIVER AT CANTON, GA
2		1		0	02397000	COOSA RIVER NEAR ROME, GA
2		1		1	02397530	COOSA RIVER AT STATE LINE, AL/GA

Table S1. Description of sites, load data, and calculated loads used in regional nutrient SPARROW models.

Major river basin identifier	If tp_site	If tn_site	Station identifier	Station_name	WQ data program	8-digit cataloging unit	Station latitude	Station longitude	NHDPlus reach identifier	ERF1_2 reach identifier	Major river basin reach identifier	Station upstream area (km2)
2	1	1	21SC60WQ.SV-137	12 MI CR AT S-39-52 ABOVE CENTRAL OUTFALL	SC DEPT HEALTH & E	03060101	34.7427	-82.8022	6266958	10363	663750	359
2	1	1	21SC60WQ.SV-325	UPPER THREE RUNS CK AT ROAD A AT SRP	SC DEPT HEALTH & E	03060106	33.2389	-81.7439	22723903	6617	664160	529
2	1	1	21SC60WQ.SV-326	FOUR MILE CK AT ROAD A-7 AT SRP	SC DEPT HEALTH & E	03060106	33.2354	-81.6978	22723667	6615	663780	34
2	1	1	21SC60WQ.SV-328	LOWER THREE RUNS CK AT S-06-20 7.5 MI SW BR	SC DEPT HEALTH & E	03060106	33.1758	-81.4809	22723771	6604	663790	160
2	0	1	21SC60WQ.SV-346	ROCKY RIVER AT S-04-244	SC DEPT HEALTH & E	03060103	34.3832	-82.5771	11735427	6482	663800	289
2	1	1	21SC60WQ.SV-354	STEVENS CREEK AT S-33-88/S-19-143	SC DEPT HEALTH & E	03060107	33.6885	-82.1498	11730084	6675	663810	1470
2	1	1	21VASWCB.4ABAN005.58	ROUTE 360 BRIDGE NE OF S BOSTON	VA DEPT OF ENVIRO	03010105	36.7456	-78.8419	8710699	4771	4771	1495
2	1	1	21VASWCB.4ABOR000.62	ROUTE 712 BRIDGE, NEAR CONFLUENCE WITH R	VA DEPT OF ENVIRO	03010101	37.1389	-79.2442	8627315	4557	4557	1002
2	1	1	21VASWCB.4ADAN015.30	RT. 501 ABOVE TOWN OF S. BOSTON	VA DEPT OF ENVIRO	03010104	36.6939	-78.9007	8693827	4738	4738	7157
2	1	1	21VASWCB.4ADAN042.80	RT. 62 AT VA. - N.C. STATE LINE	VA DEPT OF ENVIRO	03010104	36.5408	-79.2144	8696463	4746	4746	6064
2	1	1	21VASWCB.4ADAN053.35	STA. #10 AT DGIF BOAT RAMP BELOW DANVILLE S	VA DEPT OF ENVIRO	03010104	36.5606	-79.3639	8696461	4750	4750	5482
2	1	1	21VASWCB.4ADAN075.22	RT. 880 BRIDGE AT STATE LINE	VA DEPT OF ENVIRO	03010103	36.5414	-79.6053	8673773	4653	4653	4598
2	1	1	21VASWCB.4AFRV002.78	OFF ROUTE 600	VA DEPT OF ENVIRO	03010102	37.0489	-78.9294	8648430	4623	4623	591
2	1	1	21VASWCB.4ANMR002.60	NORTH MAYO AT GAGE NEAR SPENCER RT. 629 E	VA DEPT OF ENVIRO	03010103	36.5681	-79.9875	8673051	4689	4689	279
2	1	1	21VASWCB.4APGG003.29	ROUTE 605 BRIDGE	VA DEPT OF ENVIRO	03010101	36.9886	-79.5158	8632185	10626	10626	1010
2	1	1	21VASWCB.4AROA059.12	ROUTE 360 BRIDGE, EAST OF CLOVER	VA DEPT OF ENVIRO	03010102	36.8381	-78.6684	8653074	10114	10114	8342
2	1	1	21VASWCB.4AROA067.91	RT.746 BRIDGE (WATKINS BRIDGE) NEAR RANDO	VA DEPT OF ENVIRO	03010102	36.9153	-78.7406	8653008	4612	4612	7672
2	1	1	21VASWCB.4AROA097.46	ROANOKE RIVER AT BROOKNEAL GAGE , RT. 501	VA DEPT OF ENVIRO	03010102	37.0403	-78.9514	8648984	4632	4632	6215
2	1	1	21VASWCB.4AROA129.55	ROUTE 29 BRIDGE, AT GAGE - PITTSYLVANIA COL	VA DEPT OF ENVIRO	03010101	37.105	-79.2956	8626299	4566	4566	4605
2	1	1	21VASWCB.4AROA227.42	RT. 773 AT GAGING STA. IN LAFAYEETTE	VA DEPT OF ENVIRO	03010101	37.2369	-80.2092	8629291	4577	4577	659
2	1	1	21VASWCB.4ASMR016.09	GAGE NEAR NETTLE RIDGE, RT 700 BRIDGE	VA DEPT OF ENVIRO	03010103	36.5708	-80.1297	8675163	4699	4699	229
2	1	1	21VASWCB.4ASRE007.90	RT. 622 BRIDGE, MORGAN FORD BRIDGE	VA DEPT OF ENVIRO	03010103	36.5586	-79.7461	8673065	4659	4659	1388
2	1	1	21VASWCB.4ASRE022.71	FOOT BRIDGE ABOVE MARTINSVILLE STP OUTFALL	VA DEPT OF ENVIRO	03010103	36.6461	-79.8369	8673529	4664	4664	1001
2	1	1	21VASWCB.4ASRE033.19	RT. 701 BELOW FIELDCREST MILL	VA DEPT OF ENVIRO	03010103	36.7011	-79.9331	8672015	4666	4666	843
2	1	1	21VASWCB.4ASRE075.69	RT. 708 BRIDGE	VA DEPT OF ENVIRO	03010103	36.7792	-80.2481	8675427	4667	4667	69
2	1	1	21VASWCB.5ABLW014.28	RT. 58 BRIDGE	VA DEPT OF ENVIRO	03010202	36.6806	-76.9178	11585548	4861	4861	1720
[illegible]	[illegible]	[illegible]	[illegible]	[illegible]	[illegible]	[illegible]	[illegible]	[illegible]	[illegible]	[illegible]	[illegible]	[illegible]

Station identifier	DEM-estimated drainage area (km2)	RF1 estimate of mean flow (ft3/s)	Flow station identifier	Daily flow record start date for prediction'Flow station upstream area (km2)	Daily flow record start date for prediction	Daily flow record end date for prediction	Mean daily flow (ft3/s)	Standard deviation of daily flow (ft3/s)	No. days with valid flow data for prediction	Daily flow record start date for estimation	Daily flow record end date for estimation	If flow stats detrended to base year	Mean detrended daily flow (ft3/s)	Std. dev. detrended daily flow (ft3/s)
21SC60WQ.SV-137	357.44995	809.95	02186000	275	10/1/1989	9/30/2001	246.78423	308.03507	4383	6/8/1989	9/30/2001	1	147.6904	185.6377
21SC60WQ.SV-325	505.26999	119.87	02197315	526	10/1/1974	9/30/2002	235.92709	106.34965	9497	10/1/1974	9/30/2002	1	199.9706	90.0795
21SC60WQ.SV-326	42.150002	31.68	02197342	34	10/1/1976	9/30/2002	16.93004	21.87953	9131	10/1/1974	9/30/2002	1	13.6689	18.3077
21SC60WQ.SV-328	159.62	30.59	02197400	153	10/1/1974	9/30/2002	82.63927	60.83563	9497	10/1/1974	9/30/2002	1	59.6017	44.8665
21SC60WQ.SV-346	286.57	163.04	02187910	287	10/1/1989	9/30/2001	130.43072	209.58453	2921	5/25/1989	9/30/2003	1	115.8642	187.2152
21SC60WQ.SV-354	1470.42	552.58	02196000	1411	10/1/1974	9/30/2003	411.09738	1179.94747	8400	10/1/1974	9/30/2003	1	288.4284	853.3683
21VASWCB.4ABAN005.58	1544.7198	553.94	02077000	1417	10/1/1974	9/30/2004	553.80324	962.79388	10593	10/1/1974	9/30/2004	1	506.6916	880.6911
21VASWCB.4ABOR000.62	1024.97	304.59	02061500	829	10/1/1974	9/30/2004	417.46423	855.49728	10958	10/1/1974	9/30/2004	1	380.6694	786.0612
21VASWCB.4ADAN015.30	7211.8799	3039.4	02075500	6604	10/1/1974	9/30/2004	3102.96913	3821.20916	10958	10/1/1974	9/30/2004	1	2948.1551	3625.3027
21VASWCB.4ADAN042.80	6055.5103	2632.56	02075045	5452	10/1/1995	9/30/2004	2386.51583	3149.62617	3288	10/1/1995	9/30/2004	1	2268.7247	2975.484
21VASWCB.4ADAN053.35	5498.1201	2430.46	02075045	5452	10/1/1995	9/30/2004	2157.47528	2847.34781	3288	10/1/1995	9/30/2004	1	2050.9889	2689.9185
21VASWCB.4ADAN075.22	4697.29	2087.03	02075045	5452	10/1/1995	9/30/2004	1809.68466	2388.34795	3288	10/1/1995	9/30/2004	1	1720.3642	2256.2967
21VASWCB.4AFRV002.78	621.88995	243.68	02064000	448	10/1/1974	9/30/2004	223.41962	541.28097	10958	10/1/1974	9/30/2004	1	212.8629	517.5779
21VASWCB.4ANMR002.60	283.06	133.99	02070000	280	10/1/1974	9/30/2004	135.4814	214.73406	10958	10/1/1974	9/30/2004	1	123.391	194.2395
21VASWCB.4APGG003.29	1017.95	316.22	02058400	906	10/1/1974	9/30/2004	434.26969	856.18663	10958	10/1/1974	9/30/2004	1	431.7159	850.8952
21VASWCB.4AROA059.12	8451.2393	2702.59	02066000	7710	10/1/1974	9/30/2004	3355.13313	4735.12541	10958	10/1/1974	9/30/2004	1	3294.114	4643.3967
21VASWCB.4AROA067.91	7759.5396	2454.05	02066000	7710	10/1/1974	9/30/2004	3085.60555	4354.73905	10958	10/1/1974	9/30/2004	1	3029.4882	4270.3791
21VASWCB.4AROA097.46	6252.8999	1910.85	02062500	6255	10/1/1974	9/30/2004	2480.23947	3644.05008	10958	10/1/1974	9/30/2004	1	2574.5003	3795.3922
[illegible]	[illegible]	[illegible]	[illegible]	[illegible]	[illegible]	[illegible]	[illegible]	[illegible]	[illegible]	[illegible]	[illegible]	[illegible]	[illegible]	[illegible]

Station identifier	TN record start date	TN record end date	Number of TN observations	Number of censored TN observations	Median days between TN observations	Max. days between TN observations	Mean flow for TN observ. days (ft3/s)	Std. dev. flow for TN obs. days (ft3/s)	TN model root mean square error	TN model estimation method code	TN mean annual load (kg/yr)	Std. error TN mean annual load (kg/yr)	Std. dev. TN daily load (kg/yr)	TN nested area	TN model least squares weight	LN TN mean annual load	LN TN predicted mean annual load	LN TN residual
21SC60WQ.SV-137	11/17/1995	9/24/2001	32	7	29	1134	220.7561	274.0036	0.2965	2.1	75495.1825	3736.5983	265.699	39.64	1	11.23182413	11.42257078	-0.19074665
21SC60WQ.SV-325	1/18/1984	9/9/2002	189	9	29	844	243.7436	119.8423	0.6038	2.3	70169.9897	8285.2962	157.0384	505.27	1	11.158676	11.60672673	-0.448050725
21SC60WQ.SV-326	1/18/1984	9/9/2002	188	4	29	840	19.6474	20.0982	0.6644	2.3	24766.9078	3672.0175	90.0543	42.15	1	10.11726368	9.01916861	1.098095068
21SC60WQ.SV-328	12/21/1983	12/12/2000	197	5	30	156	77.6901	62.5675	0.3974	2.3	20627.3561	1357.5303	52.9029	159.62	1	9.93437344	10.28425879	-0.349885348
21SC60WQ.SV-346	1/4/1995	9/15/2003	29	0	47	1064	121.1865	203.3694	0.2275	2.3	133760.1871	9364.8363	296.5043	286.57	1	11.80380383	11.85678888	-0.052985049
21SC60WQ.SV-354	11/13/1995	9/29/2003	41	1	41	1113	226.4284	580.6931	0.3727	2.3	209605.4241	75918.9317	2644.7379	1470.42	1	12.25298211	12.55143334	-0.298451231
21VASWCB.4ABAN005.58	11/13/1974	8/26/2004	281	8	33	164	590.1798	980.933	0.4083	2.3	325963.8816	21173.954	2359.2155	1544.71	1	12.69454186	12.74196003	-0.047418167
21VASWCB.4ABOR000.62	2/23/1993	9/13/2004	118	4	30	153	383.4574	413.9212	0.342	2.3	274200.82	21220.7992	2653.2291	1024.97	1	12.52161604	12.55477884	-0.033162806
21VASWCB.4ADAN015.30	11/13/1974	8/26/2004	284	4	33	217	3306.4334	4020.1726	0.3825	2.3	2266363.022	154504.707	8838.0542	1156.37	1	14.63368691	14.57121371	0.062473201
21VASWCB.4ADAN042.80	10/16/1995	2/26/2001	63	1	29	71	3427.9223	5860.086	0.2699	2.1	1889448.231	76171.74	8543.1432	557.39	1	14.4517954	14.22566103	0.226134374
21VASWCB.4ADAN053.35	8/21/1997	6/12/2003	29	0	70	182	1614.9301	1334.2137	0.2636	2.3	1394607.255	133012.4649	3425.1753	800.83	1	14.1481234	14.19276016	-0.044636763
21VASWCB.4ADAN075.22	10/30/1995	9/23/2004	87	1	33	93	1583.4	1181.6051	0.333	2.3	1093545.127	83716.8126	3954.7659	448.73	1	13.90493539	13.98165917	-0.076723784
21VASWCB.4AFRV002.78	9/2/1997	3/13/2003	30	0	70	106	156.5407	362.0772	0.3519	2.3	212731.064	69796.9856	4734.4142	621.89	1	12.26778404	12.13945365	0.128330389
21VASWCB.4ANMR002.60	7/23/1979	6/25/2003	127	19	35	3296	114.0188	126.7339	0.4096	2.3	74210.7997	9682.2706	848.8027	283.06	1	11.21466497	10.92240207	0.292262895
21VASWCB.4APGG003.29	2/23/1993	8/31/2004	39	1	58	1258	380.0071	306.0774	0.3537	2.3	298292.3312	65646.6748	3036.0126	1017.95	1	12.60582926	12.37418503	0.231644237
21VASWCB.4AROA059.12	11/13/1974	9/9/2004	287	10	32	206	3528.9429	4452.3214	0.3464	2.3	2117209.691	87609.651	12612.7589	691.7	1	14.5656096	14.5482132	0.017396393
21VASWCB.4AROA067.91	7/2/1990	8/18/2004	68	2	66	191	2716.5671	2772.4044	0.3143	2.3	1944913.747	284047.7587	16016.5659	884.74	1	14.48072819	14.59126933	-0.110541141
21VASWCB.4AROA097.46	9/11/1979	9/30/2004	264	2	30	157	2386.3892	3099.812	0.2955	2.3	1780191.272	75125.892	9682.6129	554.59	1	14.39223137	14.19211648	0.200114895
[illegible]	[illegible]	[illegible]	[illegible]	[illegible]	[illegible]	[illegible]	[illegible]	[illegible]	[illegible]	[illegible]	[illegible]	[illegible]	[illegible]	[illegible]	[illegible]	[illegible]	[illegible]	[illegible]

Station identifier	TP record start date	TP record end date	Number of TP observations	Number of censored TP observations	Median days between TP observations	Max. days between TP observations	Mean flow for TP observ. days (ft3/s)	Std. dev. flow for TP obs. days (ft3/s)	TP model root mean square error	TP model estimation method code	TP mean annual load (kg/yr)	Std. error TP mean annual load (kg/yr)	Std. dev. TP daily load (kg/yr)	TP nested area	TP model least squares weight	LN TP mean annual load	LN TP predicted mean annual load	LN TP residual
21SC60WQ.SV-137	6/29/1989	10/22/1996	30	2	30	918	261.7772	257.2096	0.5739	2.1	5456.8029	1181.503	77.7138	39.64	1	8.604618348	9.272969101	-0.668350753
21SC60WQ.SV-325	1/18/1984	9/9/2002	177	48	29	1385	245.1361	121.6855	0.6689	2.3	6742.5518	1029.9764	24.425	505.27	1	8.816193738	9.471511551	-0.655317813
21SC60WQ.SV-326	1/18/1984	9/9/2002	175	51	29	1385	19.9155	20.4293	0.8317	2.3	1963.7989	400.5913	15.419	42.15	1	7.582636091	6.378886881	1.20374921
21SC60WQ.SV-328	12/21/1983	6/1/1998	170	48	30	156	83.7754	65.2331	0.7105	2.1	2638.5289	188.0451	8.8622	159.62	1	7.877976806	7.099232333	0.778744473
21SC60WQ.SV-346																		
21SC60WQ.SV-354	11/13/1995	9/29/2003	30	8	35	1920	190.6718	278.1988	0.3724	2.3	14639.303	4681.7224	194.4581	1470.42	1	9.591465177	10.33153856	-0.740073379
21VASWCB.4ABAN005.58	8/7/1979	8/26/2004	249	61	31	164	602.9768	1029.5255	0.4843	2.3	35165.8311	2819.3389	373.1078	1544.71	1	10.46783018	10.3806346	0.087195578
21VASWCB.4ABOR000.62	2/23/1993	9/13/2004	119	24	30	153	409.4914	500.5327	0.4935	2.3	47424.0073	6445.1509	1422.2975	1024.97	1	10.76688386	10.25268141	0.514202455
21VASWCB.4ADAN015.30	8/7/1979	8/26/2004	259	12	31	164	3477.8982	4321.9677	0.4895	2.3	295101.2336	22877.1873	1820.5086	1156.37	1	12.59507374	12.71566477	-0.120591027
21VASWCB.4ADAN042.80	10/16/1995	2/26/2001	63	0	29	71	3427.9223	5860.086	0.4103	2.1	312675.4628	28756.5381	2877.8803	557.39	1	12.65292107	11.97336524	0.679555833
21VASWCB.4ADAN053.35	8/21/1997	6/12/2003	30	0	69	126	1595.9555	1315.1212	0.3101	2.3	148195.8935	16523.6815	357.9445	800.83	1	11.90629028	11.9799918	-0.073701519
21VASWCB.4ADAN075.22	10/30/1995	9/23/2004	87	15	33	93	1583.4	1181.6051	0.495	2.3	128291.4289	15695.0701	537.7155	448.73	1	11.76205974	12.10515021	-0.343090463
21VASWCB.4AFRV002.78	9/2/1997	3/13/2003	31	5	66	105	154.5117	356.1706	0.6106	2.3	13159.2975	8536.8808	474.9006	621.89	0.5	9.484883822	9.742551851	-0.257668029
21VASWCB.4ANMR002.60	7/23/1979	6/25/2003	127	68	35	3296	114.0188	126.7339	0.5973	2.3	9973.9679	2197.335	241.3375	283.06	1	9.207733768	8.482879085	0.724854683
21VASWCB.4APGG003.29	2/23/1993	8/31/2004	40	4	58	1258	466.6521	625.7594	0.5292	2.3	34476.5957	9937.7556	962.7277	1017.95	1	10.44803599	9.829699943	0.618336044
21VASWCB.4AROA059.12	8/7/1979	9/9/2004	262	64	30	160	3575.1294	4384.154	0.5521	2.3	249217.8795	21652.9886	2187.2063	691.7	1	12.42608281	12.27109252	0.154990291
21VASWCB.4AROA067.91	2/1/1990	8/18/2004	83	2	63	191	3156.2902	3289.8988	0.4336	2.3	201042.1386	24202.1755	2013.0396	884.74	1	12.21126981	12.37252011	-0.161250303
21VASWCB.4AROA097.46	9/11/1979	9/30/2004	265	105	30	157	2443.0068	3228.2981	0.5184	2.3	211351.1684	13013.3134	1643.6276	554.59	1	12.26127633	11.84337364	0.41790269
[illegible]	[illegible]	[illegible]	[illegible]	[illegible]	[illegible]	[illegible]	[illegible]	[illegible]	[illegible]	[illegible]	[illegible]	[illegible]	[illegible]	[illegible]	[illegible]	[illegible]	[illegible]	[illegible]

Table S1. Description of sites, load data, and calculated loads used in regional nutrient SPARROW models.

Major river basin identifier	tp	If site	tn	If site	Station identifier	Station name	WQ data program	8-digit cataloging unit	Station latitude	Station longitude
3		1		1	05316500	REDWOOD RIVER NEAR REDWOOD FALLS, MN	MNPCA1	07020006	44.52366	-95.17169
3		1		1	05317000	COTTONWOOD RIVER NEAR NEW ULM, MN	MNPCA1	07020008	44.29119	-94.44053
3		1		1	05319500	WATONWAN RIVER NEAR GARDEN CITY, MN	MNPCA1	07020010	44.0465	-94.19482
3		1		1	05320270	LITTLE COBB RIVER NEAR BEAUFORD, MN	USGS	07020011	43.99641	-93.90871
3		1		1	05322000	BLUE EARTH RIVER AT MOUTH AT MANKATO, MN	MNPCA1	07020009	44.1633	-94.0369
3		1		0	05325000	MINNESOTA RIVER AT MANKATO, MN	USGS	07020007	44.16966	-94.00307
3		1		1	05330000	MINNESOTA RIVER NEAR JORDAN, MN	USGS	07020012	44.69314	-93.64191
3		1		1	05331000	MISSISSIPPI RIVER AT ST. PAUL, MN	21MINN	07010206	44.93309	-93.10457
3		1		1	05331570	MISSISSIPPI RIVER AT NININGER, MN	USGS	07010206	44.77583	-92.89836
3		1		1	05331580	MISSISSIPPI RIVER BELOW L&D #2 AT HASTINGS, MN	USGS	07010206	44.74663	-92.85243
3		1		1	05333500	ST. CROIX RIVER NEAR DANBURY, WI	USGS	07030001	46.07524	-92.24766
3		1		1	05338500	SNAKE RIVER NEAR PINE CITY, MN	MNPCA1	07030004	45.84057	-92.93188
3		1		1	05340500	ST. CROIX RIVER AT ST. CROIX FALLS, WI	USGS	07030005	45.40688	-92.64824
3		1		1	05355200	CANNON RIVER AT WELCH, MN	MNPCA1	07040002	44.5645	-92.73164
3		1		0	05367500	RED CEDAR RIVER NEAR COLFAX, WI	21WIS	07050007	45.05308	-91.7119
3		1		0	05368000	HAY RIVER AT WHEELER, WI	21WIS	07050007	45.04785	-91.91102
3		1		1	05369500	CHIPPEWA RIVER AT DURAND, WI	USGS	07050005	44.62891	-91.97018
3		1		1	05378183	JOOS VALLEY CREEK NEAR FOUNTAIN CITY, WI	21WIS	07040003	44.21479	-91.66514
3		1		0	05378185	EAGLE CREEK AT CT HIGHWAY G NEAR FOUNTAI	USGS	07040003	44.2094	-91.67838
3		1		1	05378500	MISSISSIPPI RIVER AT WINONA, MN	USGS	07040003	44.05672	-91.63669
3		1		1	05379500	TREMPEALEAU RIVER AT DODGE, WI	21WIS	07040005	44.1316	-91.55309
3		1		1	05382000	BLACK RIVER NEAR GALESVILLE, WI	21WIS	07040007	44.06063	-91.28719
3		1		1	05388250	Upper Iowa River near Dorchester, IA	21IOWA	07060002	43.42111	-91.50884
3		1		1	054	MISSISSIPPI RIVER AT BR ON MN-25 AT MONTICELLO	21MINN	07010203	45.30886	-93.79187
3		1		1	05401050	TENMILE CREEK NEAR NEKOOSA, WI	USGS	07070003	44.26262	-89.8106
3		1		1	05405000	BARABOO RIVER NEAR BARABOO, WI	21WIS	07070004	43.48174	-89.63648
3		1		1	05406491	GARFOOT CREEK NEAR CROSS PLAINS, WI	21WIS	07070005	43.10998	-89.67937
3		1		1	05406500	BLACK EARTH CREEK AT BLACK EARTH, WI	USGS	07070005	43.13388	-89.73253
3		1		1	05407000	WISCONSIN RIVER AT MUSCODA, WI	USGS	07070005	43.1995	-90.44326
3		1		1	05407500	KICKAPOO RIVER AT ONTARIO, WI	21WIS	07070006	43.71476	-90.58685
3		1		1	05412500	Turkey River at Garber, IA	21IOWA	07060004	42.73972	-91.26163
3		1		1	05413500	GRANT RIVER AT BURTON, WI	USGS	07060003	42.72037	-90.81953
3		1		1	05418500	Maquoketa River near Maquoketa, IA	21IOWA	07060006	42.08363	-90.63249
3		1		0	05418950	APPLE RIVER NEAR ELIZABETH, IL	21ILAMB	07060005	42.31862	-90.25429
3		1		1	05420500	Mississippi River at Clinton, IA	USGS	07080101	41.77928	-90.25105
3		1		1	05420680	Wapsipinicon River near Tripoli, IA	USGS	07080102	42.83627	-92.25706
3		1		1	05422000	Wapsipinicon River near De Witt, IA	21IOWA	07080103	41.76649	-90.53472
3		1		0	05423000	WEST BRANCH ROCK RIVER NEAR WAUPUN, WI	21WIS	07090001	43.66803	-88.65244
3		1		0	05423510	WEST BRANCH ROCK RIVER @ STATE HWY 49 NR	USGS	07090001	43.63383	-88.68463
3		1		1	05424057	ROCK RIVER AT HORICON, WI	USGS	07090001	43.4501	-88.63234
3		1		1	05424082	ROCK RIVER AT HUSTISFORD, WI	USGS	07090001	43.34537	-88.59803
3		1		1	05425500	ROCK RIVER AT WATERTOWN, WI	USGS	07090001	43.18806	-88.72647
3		1		1	05426000	CRAWFISH RIVER AT MILFORD, WI	21WIS	07090002	43.09992	-88.84968
3		1		1	05427085	ROCK RIVER AT ROBERT STREET AT FORT ATKIN	USGS	07090001	42.92751	-88.84288
3		1		1	05427570	ROCK RIVER AT INDIANFORD, WI	21WIS	07090001	42.80392	-89.08915
3		1		1	05430175	YAHARA RIVER NEAR FULTON, WI	USGS	07090001	42.82649	-89.17245
3		1		1	05430500	ROCK RIVER AT AFTON, WI	21WIS	07090001	42.60937	-89.0704
3		1		1	05431486	TURTLE CREEK AT CARVERS ROCK ROAD NEAR (	USGS	07090001	42.59752	-88.82898
3		1		1	05434500	PECATONICA RIVER AT MARTINTOWN, WI	21WIS	07090003	42.50961	-89.79931
3		1		0	05435500	PECATONICA RIVER AT FREEPORT, IL	21ILAMB	07090003	42.30304	-89.619
3		1		0	05435800	PECATONICA RIVER AT HARRISON, IL	21ILAMB	07090003	42.42751	-89.19567
3		1		1	05437500	ROCK RIVER AT ROCKTON, IL	USGS	07090005	42.44956	-89.06969
3		1		0	05438250	COON CREEK AT RILEY, IL	21ILAMB	07090006	42.1828	-88.64126
3		1		0	05438600	KISHWAUKEE R AB SOUTH BRANCH NR PERRYVII	21ILAMB	07090006	42.2018	-88.97919
3		1		0	05439500	SOUTH BRANCH KISHWAUKEE RIVER NR FAIRDALE, IL	21ILAMB	07090006	42.11032	-88.90084
3		1		0	05440000	KISHWAUKEE RIVER NEAR PERRYVILLE, IL	21ILAMB	07090006	42.19379	-89.0002
3		1		1	05440700	ROCK RIVER AT BYRON, IL	21ILAMB	07090005	42.12273	-89.25566
3		1		1	05442200	ROCK RIVER AT GRAND DETOUR, IL	21ILAMB	07090005	41.88998	-89.42055
3		1		1	05443500	ROCK RIVER AT COMO, IL	21ILAMB	07090005	41.78184	-89.7504
3		1		0	05444000	ELKHORN CREEK NEAR PENROSE, IL	USGS	07090005	41.90255	-89.69672
3		1		1	05446500	ROCK RIVER NEAR JOSLIN, IL	USGS	07090005	41.55601	-90.18526
3		1		1	05447500	GREEN RIVER NEAR GENESEO, IL	21ILAMB	07090007	41.48886	-90.15783
3		1		1	05449500	Iowa River near Rowan, IA	USGS	07080207	42.75993	-93.62229
3		1		1	05451210	South Fork Iowa River NE of New Providence, IA	USGS	07080207	42.31483	-93.15241
3		1		1	05453100	Iowa River at Marengo, IA	USGS	07080208	41.81218	-92.06426
3		1		1	05455100	Old Mans Creek near Iowa City, IA	21IOWA	07080209	41.60642	-91.6157
3		1		1	05458000	Little Cedar River near Ionia, IA	21IOWA	07080201	43.03352	-92.5037
3		1		1	05458900	West Fork Cedar River at Finchford, IA	21IOWA	07080204	42.62936	-92.54324
3		1		1	05463050	Cedar River at Cedar Falls, IA	USGS	07080205	42.53935	-92.44966
3		1		1	05464020	Cedar River at Gilbertville, IA	21IOWA	07080205	42.41582	-92.21879
3		1		1	05464220	Wolf Creek near Dysart, IA	USGS	07080205	42.2517	-92.29882
3		1		1	05464760	Cedar River near Bertram, IA	21IOWA	07080206	41.92572	-91.55024
3		1		1	05465500	Iowa River at Wapello, IA	USGS	07080209	41.17835	-91.18098
3		1		0	05466500	EDWARDS RIVER NEAR NEW BOSTON, IL	21ILAMB	07080104	41.18696	-90.96764
3		1		0	05469000	HENDERSON CREEK NEAR OQUAWKA, IL	21ILAMB	07080104	41.00188	-90.8543
3		1		1	05471500	South Skunk River near Oskaloosa, IA	21IOWA	07080105	41.35524	-92.65707
3		1		1	05474000	Skunk River at Augusta, IA	USGS	07080107	40.75317	-91.27611
3		1		1	05474500	Mississippi River at Keokuk, IA	USGS	07080104	40.39247	-91.37181
3		1		0	05476000	DES MOINES RIVER AT JACKSON, MN	USGS	07100001	43.61937	-94.98427
3		1		1	05481650	Des Moines River near Saylorville, IA	USGS	07100004	41.68033	-93.66881
3		1		1	05484500	Raccoon River at Van Meter, IA	21IOWA	07100006	41.53414	-93.94991
3		1		1	05486000	North River near Norwalk, IA	21IOWA	07100008	41.45784	-93.65503
3		1		1	05490600	Des Moines River at St. Francisville, MO	USGS	07100009	40.46211	-91.56707
3		1		1	05495000	Fox River at Wayland, MO	USGS	07110001	40.39215	-91.59814
3		1		0	05495500	BEAR CREEK NEAR MARCELLINE, IL	21ILAMB	07110001	40.14358	-91.34146
3		1		1	05500000	South Fabius River near Taylor, MO	USGS	07110003	39.89678	-91.57987
3		1		1	05514500	Cuivre River near Troy, MO	USGS	07110008	39.00937	-90.97636
3		1		1	05518000	KANKAKEE RIVER AT SHELBY, IN	21IND	07120001	41.18248	-87.34354
3		1		1	05520500	KANKAKEE RIVER AT MOMENCE, IL	USGS	07120001	41.15944	-87.66816
3		1		0	05525000	IROQUOIS RIVER AT IROQUOIS, IL	USGS	07120002	40.82321	-87.58175
3		1		1	05525500	SUGAR CREEK AT MILFORD, IL	USGS	07120002	40.63164	-87.7243
3		1		1	05526000	IROQUOIS RIVER NEAR CHEBANSE, IL	USGS	07120002	41.00908	-87.82526
3		1		1	05527500	KANKAKEE RIVER NEAR WILMINGTON, IL	21ILAMB	07120001	41.34607	-88.18808
3		1		1	05527800	DES PLAINES RIVER AT RUSSELL, IL	USGS	07120004	42.48755	-87.92545
3		1		1	05528000	DES PLAINES RIVER NEAR GURNEE, IL	21ILAMB	07120004	42.34398	-87.94056
3		1		0	05529000	DES PLAINES RIVER NEAR DES PLAINES, IL	21ILAMB	07120004	42.08194	-87.89028
3		1		1	05530590	DES PLAINES RIVER NEAR SCHILLER PARK, IL	21ILAMB	07120004	41.95333	-87.85458
3		1		1	05531500	SALT CREEK AT WESTERN SPRINGS, IL	USGS	07120004	41.82604	-87.89995
3		1		1	05532500	DES PLAINES RIVER AT RIVERSIDE, IL	USGS	07120004	41.82278	-87.82005
3		1		1	05534050	DES PLAINES RIVER AT LOCKPORT, IL	21ILAMB	07120004	41.59642	-88.06861
3		1		1	05536195	LITTLE CALUMET RIVER AT MUNSTER, IND.	21IND	07120003	41.57715	-87.52229
3		1		1	05536995	CHICAGO SANITARY AND SHIP CANAL AT ROMEO	USGS	07120004	41.64088	-88.05999
3		1		0	05539000	HICKORY CREEK AT JOLIET, IL	21ILAMB	07120004	41.51445	-88.07413
3		1		0	05539900	WEST BRANCH DU PAGE RIVER NEAR WEST CHIC	21ILAMB	07120004	41.91086	-88.17918
3		1		1	05540095	WEST BRANCH DU PAGE RIVER NEAR WARRENVI	21ILAMB	07120004	41.82189	-88.17244
3		1		0	05540210	EAST BRANCH DU PAGE RIVER AT ROUTE 34 AT L	21ILAMB	07120004	41.80059	-88.08145
3		1		0	05540290	DU PAGE RIVER NEAR NAPERVILLE, IL	21ILAMB	07120004	41.69003	-88.16618
3		1		1	05540500	DU PAGE RIVER AT SHOREWOOD, IL	USGS	07120004	41.5223	-88.1932
3		1		0	05542000	MAZON RIVER NEAR COAL CITY, IL	21ILAMB	07120005	41.28711	-88.36064
3		1		1	05543500	ILLINOIS RIVER AT MARSEILLES, IL	USGS	07120005	41.3259	-88.71807
3		1		0	055451345	WHITE RIVER AT CENTER STREET AT LAKE GENE	USGS	07120006	42.59057	-88.43371
3		1		1	05546700	FOX RIVER NEAR CHANNEL LAKE, IL	21ILAMB	07120006	42.47919	-88.17833
3		1		1	05548280	NIPPERSINK CREEK NEAR SPRING GROVE, IL	21ILAMB	07120006	42.44352	-88.24723
3		1		1	05549600	FOX RIVER AT BURTONS BRIDGE, IL	21ILAMB	07120006	42.27948	-88.22711
3		1		1	05550000	FOX RIVER AT ALGONQUIN, IL	USGS	07120006	42.16596	-88.29041
3		1		1	05551000	FOX RIVER AT SOUTH ELGIN, IL	21ILAMB	07120007	41.9962	-88.2941
3		1		1	05551700	BLACKBERRY CREEK NEAR YORKVILLE, IL	21ILAMB	07120007	41.67123	-88.44618
3		1		1	05551995	SOMONAUK CREEK AT SHERIDAN, IL	21ILAMB	07120007	41.54361	-88.68667
3		1		1	05552500	FOX RIVER AT DAYTON, IL	USGS	07120007	41.38325	-88.79087
3		1		1	05553500	ILLINOIS RIVER AT OTTAWA, IL	USGS	07130001	41.34199	-88.84671
3		1		0	05554490	VERMILION RIVER AT MC DOWELL, IL	21ILAMB	07130002	40.83059	-88.57478
3		1		0	05555300	VERMILION RIVER NEAR LEONORE, IL	21ILAMB	07130002	41.20959	-88.93729
3		1		0	05556200	ILLINOIS RIVER AT HENNEPIN	21ILAMB	07130001	41.25788	-89.34883
3		1		0	05556500	BIG BUREAU CREEK AT PRINCETON, IL	21ILAMB	07130001	41.36494	-89.49758
3		1		0	05558995	ILLINOIS RIVER AT LACON, IL	21ILAMB	07130001	41.02504	-89.41731
3		1		1	05559900	ILLINOIS RIVER AT WATER COMPANY AT PEORIA, IL	21ILAMB	07130001	40.72493	-89.54726
3		1		1	05563800	ILLINOIS RIVER AT PEKIN, IL	21ILAMB	07130003	40.57309	-89.65482
3		1		0	05567510	MACKINAW RIVER BELOW CONGERVILLE, IL	USGS	07130004	40.58672	-89.27846
3		1		0	05568005	MACKINAW RIVER BELOW GREEN VALLEY, IL	21ILAMB	07130004	40.44754	-89.69122
3		1		1	05568800	INDIAN CREEK NEAR WYOMING, IL	21ILAMB	07130005	41.01846	-89.83588
3		1		0	05568915	SPOON RIVER NEAR DAHINDA, IL	21ILAMB	07130005	40.90791	-90.08681
3		1		0	05569500	SPOON RIVER AT LONDON MILLS, IL	21ILAMB	07130005	40.70676	-90.28286
3		1		1	05570000	SPOON RIVER AT SEVILLE, IL	USGS	07130005	40.48497	-90.34219
3		1		0	05570520	ILLINOIS RIVER AT POWER COMPANY AT HAVANA	21ILAMB	07130003	40.28032	-90.08151
3		1		1	05570910	SANGAMON RIVER AT FISHER, IL	21ILAMB	07130006	40.31119	-88.32265
3		1		1	05572000	SANGAMON RIVER AT MONTICELLO, IL	USGS	07130006	40.02573	-88.58914
3		1		1	05572125	SANGAMON R AT ALLERTON PARK NEAR MONTIC	21ILAMB	07130006	40.00226	-88.63534
3		1		1	05573504	SANGAMON R. AT L DECATUR WATER INTAKE AT	21ILAMB	07130006	39.82893	-88.95972
3		1		0	05573540	SANGAMON RIVER AT ROUTE 48 AT DECATUR, IL	21ILAMB	07130006	39.83094	-88.97628
3		1		0	05573650	SANGAMON RIVER NEAR NIANTIC, IL	21ILAMB	07130006	39.79723	-89.10541
3		1		0	05573800	SANGAMON RIVER AT ROBY, IL	21ILAMB	07130006	39.74227	-89.39917
3		1		1	05575500	SOUTH FORK SANGAMON RIVER AT KINCAID, IL	21ILAMB	07130007	39.57884	-89.39252
3		1		0	05576022	SOUTH FORK SANGAMON RIVER BELOW ROCHES	21ILAMB	07130007	39.75411	-89.56615
3		1		1	05576500	SANGAMON RIVER AT RIVERTON, IL	21ILAMB	07130008	39.84754	-89.55004
3		1		0	05577505	SPRING CR AT BURNS LANE BRIDGE AT SPRINGF	21ILAMB	07130008	39.82055	-89.68765
3		1		0	05578000	SANGAMON RIVER AT PETERSBURG, IL	21ILAMB	07130008	40.01102	-89.84401
3		1		0	05578500	SALT CREEK NEAR ROWELL, IL	21ILAMB	07130009	40.11457	-89.05048
3		1		0	05579500	LAKE FORK NEAR CORNLAND, IL	21ILAMB	07130009	39.95123	-89.38413
3		1		0	05580000	KICKAPOO CREEK AT WAYNESVILLE, IL	21ILAMB	07130009	40.25517	-89.12829
3		1		0	05580500	KICKAPOO CREEK NEAR LINCOLN, IL	21ILAMB	07130009	40.1864	-89.36675
3		1		0	05582000	SALT CREEK NEAR GREENVIEW, IL	21ILAMB	07130009	40.13304	-89.73666
3		1		1	05583000	SANGAMON RIVER NEAR OAKFORD, IL	USGS	07130008	40.12302	-89.98523
3		1		1	05584500	LA MOINE RIVER AT COLMAR, IL	USGS	07130010	40.33088	-90.89598
3		1		1	05585000	LA MOINE RIVER AT RIPLEY, IL	21ILAMB	07130010	40.02384	-90.63083
3		1		1	05586100	ILLINOIS RIVER AT VALLEY CITY, IL	USGS	07130011	39.70341	-90.64615
3		1		0	05587000	MACOUPIN CREEK NEAR KANE, IL	21ILAMB	07130012	39.23422	-90.39456
3		1		1	05587455	MISSISSIPPI RIVER BELOW GRAFTON, IL	USGS	07110009	38.95033	-90.37151
3		1		0	05587900	CAHOKIA CREEK AT EDWARDSVILLE, IL	21ILAMB	07140101	38.82447	-89.97496
3		1		1	05591200	KASKASKIA RIVER AT COOKS MILLS, IL	USGS	07140201	39.58315	-88.41316
3		1		1	05591300	KASKASKIA RIVER AT ALLENVILLE, IL	21ILAMB	07140201	39.57281	-88.53222
3		1		1	05591400	JONATHAN CREEK NEAR SULLIVAN, IL	21ILAMB	07140201	39.60111	-88.54611
3		1		1	05591700	WEST OKAW RIVER NEAR LOVINGTON, IL	21ILAMB	07140201	39.73133	-88.66232
3		1		1	05592000	KASKASKIA RIVER AT SHELBYVILLE, IL	21ILAMB	07140201	39.39884	-88.78805
3		1		1	05592100	KASKASKIA RIVER NEAR COWDEN, IL	USGS	07140201	39.22962	-88.84489
3		1		0	05592195	BECK CREEK AT HERRICK, IL	21ILAMB	07140201	39.21639	-89.02056
3		1		1	05592500	KASKASKIA RIVER AT VANDALIA, IL	21ILAMB	07140202	38.95644	-89.08769
3		1		1	05592600	HICKORY CREEK NEAR BLUFF CITY, IL	21ILAMB	07140202	38.92525	-89.0393
3		1		1	05592800	HURRICANE CREEK NEAR MULBERRY GROVE, IL	21ILAMB	07140202	38.91851	-89.24281
3		1		1	05592900	EAST FORK KASKASKIA RIVER NEAR SANDOVAL, IL	21ILAMB	07140202	38.69236	-89.09992
3		1		1	05592930	NORTH FORK KASKASKIA RIVER NEAR PATOKA, IL	21ILAMB	07140202	38.77361	-89.08611
3		1		1	05593010	KASKASKIA RIVER BELOW CARLYLE, IL	21ILAMB	07140202	38.57449	-89.36925
3		1		0	05593505	CROOKED CREEK NEAR ODIN, IL	21ILAMB	07140202	38.56389	-89.05028
3		1		0	05594000	SHOAL CREEK NEAR BREESE, IL	21ILAMB	07140203	38.60959	-89.49452
3		1		1	05594100	KASKASKIA RIVER NEAR VENEDY STATION, IL	USGS	07140204	38.44995	-89.62978
3		1		0	05594450	SILVER CREEK NEAR TROY, IL	21ILAMB	07140204	38.7152	-89.83039
3		1		0	05594800	SILVER CREEK NEAR FREEBURG, IL	USGS	07140204	38.39653	-89.87296
3		1		0	05595200	RICHLAND CREEK NEAR HECKER, IL	21ILAMB	07140204	38.32375	-89.97107
3		1		1	05595700	BIG MUDDY RIVER NEAR MT. VERNON, IL	21ILAMB	07140106	38.30935	-88.98868
3		1		1	05595730	RAYSE CREEK NEAR WALTONVILLE, IL	21ILAMB	07140106	38.25381	-89.03986
3		1		1	05595830	CASEY FORK AT ROUTE 37 NEAR MT. VERNON, IL	21ILAMB	07140106	38.26947	-88.8989
3		1		0	05597000	BIG MUDDY RIVER AT PLUMFIELD, IL	21ILAMB	07140106	37.90148	-89.01428
3		1		0	05597500	CRAB ORCHARD CREEK NEAR MARION, IL	21ILAMB	07140106	37.73092	-88.88947
3		1		1	05599500	BIG MUDDY RIVER AT MURPHYSBORO, IL	USGS	07140106	37.74924	-89.34713
3		1		1	07014500	Meramec River near Sullivan, MO	USGS	07140102	38.15847	-91.10858
3		1		1	07016400	Bourbeuse River above Union, MO	USGS	07140103	38.432	-91.01986
3		1		1	07018100	Big River near Richwoods, MO	USGS	07140104	38.15988	-90.70545
3		1		1	07019280	Meramec River at Paulina Hills, MO	USGS	07140102	38.46283	-90.41484
3		1		1	090162	SAGINAW R AT MIDLAND ST BR; BANGOR TWP, SE	21MICH	04080206	43.60139	-83.89194
3		1		1	093051	CHIPPEWA R @ NSP HOLCOMBE DM TAILRACE	21WIS	07050005	45.22441	-91.12812
3		1		1	10070001	Beaver Creek near Cedar Falls	21IOWA	07080205	42.5732	-92.5065
3		1		1	10070002	Wolf Creek at La Porte City	21IOWA	07080205	42.3158	-92.1936
3		1		1	10070004	Black Hawk Creek at Waterloo	21IOWA	07080205	42.4695	-92.4134
3		1		1	10090001	Cedar River near Janesville	21IOWA	07080201	42.6496	-92.4664
3		1		1	10120001	Shell Rock River at Shell Rock	21IOWA	07080202	42.7116	-92.5812
3		1		1	10160001	Cedar River at Cedar Bluff	21IOWA	07080206	41.78801	-91.31241
3		1		1	10170002	Winnebago River Upstream of Mason City	21IOWA	07080203	43.1929	-93.2103
3		1		1	10170003	Winnebago River Downstream of Mason City	21IOWA	07080203	43.1257	-93.1231
3		1		1	10220002	Volga River near Elkport	21IOWA	07060004	42.7497	-91.2766
3		1		1	10250001	South Raccoon River near Redfield	21IOWA	07100007	41.5798	-94.1827
3		1		1	103094	BLACK RIVER	21WIS	07040007	44.57076	-90.59517
3		1		1	10340001	Cedar River near Charles City	21IOWA	07080201	43.00726	-92.60293
3		1		1	10370001	North Raccoon River near Jefferson	21IOWA	07100006	42.0119	-94.4286
3		1		1	10400001	Boone River near Stratford	21IOWA	07100005	42.3411	-93.892
3		1		1	10420001	Iowa River near Gifford	21IOWA	07080207	42.3102	-93.0755
3		1		1	10440001	Cedar Creek near Oakland Mills	21IOWA	07080107	40.9254	-91.6735
3		1		1	10460001	West Fork Des Moines River near Humboldt	21IOWA	07100002	42.6744	-94.2069
3		1		1	10490001	North Fork Maquoketa River near Hurstville	21IOWA	07060006	42.09025	-90.67231
3		1		1	10490002	Maquoketa River near Maquoketa	21IOWA	07060006	42.0757	-90.6823
3		1		1	10500001	Indian Creek near Colfax	21IOWA	07080105	41.74621	-93.22642
3		1		1	10520002	Iowa River Upstream of Iowa City	21IOWA	07080209	41.6937	-91.5466
3		1		1	10520003	Iowa River Downstream of Iowa City	21IOWA	07080209	41.5547	-91.5186
3		1		1	10540001	North Skunk River	21IOWA	07080106	41.28387	-92.25744
3		1		1	10550001	East Fork of The Des Moines River near St. Joseph	21IOWA	07100003	42.95138	-94.23353
3		1		1	10570002	Cedar River Upstream of Cedar Rapids	21IOWA	07080205	42.0692	-91.7847
3		1		1	10630001	Whitebreast Creek near Knoxville	21IOWA	07100008	41.3026	-93.1713
3		1		1	10630002	Cedar Creek near Bussey	21IOWA	07100009	41.2191	-92.9077
3		1		1	10640002	Iowa River Downstream of Marshalltown	21IOWA	07080208	42.0508	-92.8464
3		1		1	10640003	Iowa River Upstream of Marshalltown	21IOWA	07080208	42.0917	-93.0002
3		1		1	10700001	Cedar River near Conesville	21IOWA	07080206	41.4093	-91.29
3		1		1	10770001	Beaver Creek near Grimes	21IOWA	07100004	41.6883	-93.7353
3		1		1	10770003	Des Moines River Downstream of Des Moines	21IOWA	07100008	41.4877	-93.3384
3		1		1	10850003	South Skunk River Upstream of Ames	21IOWA	07080105	42.06631	-93.62066
3		1		1	10900002	Des Moines River Downstream of Ottumwa	21IOWA	07100009	40.95677	-92.33997
3		1		1	10900003	Des Moines River Upstream of Ottumwa	21IOWA	07100009	41.0874	-92.5258
3		1		1	10910001	Middle River near Indianola	21IOWA	07100008	41.4247	-93.5871
3		1		1	10940002	Des Moines River Upstream of Fort Dodge	21IOWA	07100004	42.5436	-94.2163
3		1		1	123016	Mississippi R. at LD 9	21WIS	07060001	43.21167	-91.10253
3		1		1	123017	Kickapoo R. at Stueben	21WIS	07070006	43.18214	-90.85064
3		1		1	130001	Maquoketa River	USGSBRD	07060006	42.18667	-90.31056
3		1		1	130003	APPLE RIVER NEAR HANOVER, IL	USGSBRD	07060005	42.18718	-90.23599
3		1		1	130202	BATTLE CREEK R@ 9 MILE RD; PENNFIELD TWP S	21MICH	04050003	42.36417	-85.12222
3		1		1	133416	SUGAR RIVER AT STH 69 DWNSTM BRIDGE	21WIS	07090004	42.94904	-89.54451
3		1		1	133417	SUGAR RIVER AT VALLEY RD.	21WIS	07090004	42.9725	-89.56667
3		1		1	140001	Wapsipinicon River	USGSBRD	07080103	41.7273	-90.36456
3		1		0	160376	E BR DUPAGE R .5 MI S NAPERVILLE T37N R10E NE	21ILL	07120004	41.71111	-88.12778
3		1		0	160383	E BR DUPAGE R MAPLE AV LISLE T38N R10E SE10	21ILL	07120004	41.78571	-88.07922
3		1		1	163001	ST. LOUIS RIVER AT OLIVER	21-WLSSD	04010201	46.65669	-92.20044
3		1		1	163002	BOIS BRULE RIVER	21WIS	04010301	46.67936	-91.59491
3		1		0	171408	MISSISSINEWA RIVER AT PERU MILE POINT 0.8	21IND	05120103	40.75389	-86.02306
3		1		1	171420	ST. MARY'S RIVER NEAR FT WAYNE AT MI PT 10.83	21IND	04100004	40.988	-85.09897
3		1		1	171433	WABASH R AT US 27 BRIDGE N OF GENEVA, MI PT	21IND	05120101	40.61667	-84.95417
3		1		0	173051	RED CEDAR R @ CTH Y BRIDGE	21WIS	07050007	44.71927	-91.90336
3		1		1	173208	Red Cedar R. at Menomonie	21WIS	07050007	44.88336	-91.93193
3		1		1	173993	WABASH RIVER AT VINCENNES US 50 AT MI PT 128.8	21IND	05120111	38.70722	-87.51917
3		1		1	174000	WABASH R NR HUNTINGTON IND AT MILE POINT 4	21IND	05120101	40.85479	-85.49827
3		1		0	174001	WHITE RIVER NEAR HAZELTON.	21IND	05120202	38.49083	-87.56528
3		1		1	174035	WHITE R AT PERKINSVILLE MILE POINT 278.610	21IND	05120201	40.14167	-85.88
3		1		0	174303	BLUE R ON INTERSTATE 70 AT MILE POINT 63.76	21IND	05120204	39.85401	-85.47729
3		1		1	174317	WHITE RIVER AT EDWARDSPORT, MILE POINT 80.98	21IND	05120202	38.80696	-87.24588
3		1		1	174319	WHITE RIVER AT SPENCER MILE POINT 162.22	21IND	05120202	39.28778	-86.74583
3		1		0	174350	TIPPECANOE R-ROCHESTER US 31 N OF TOWN M	21IND	05120106	41.10603	-86.22039
3		1		1	174554	WHITE R-W FORK-WAVERLY S.R. 144 BR MI PT 210	21IND	05120201	39.55972	-86.27444
3		0		1	1BBFW0001	BEECH FORK LAKE	11COEHUN	05090102	38.29936	-82.39669
3		0		1	1DEWW0001	JOHNS CR OF LEVISA FORK KY	11COEHUN	05070203	37.73908	-82.73133
3		0		1	1DILW0003	LICKING CK AT TOBOSO GAGING STA	11COEHUN	05040006	40.05694	-82.22
3		1		1	1ELTW0031	East Fork Twelvepole Ck	11COEHUN	05090102	38.05222	-82.30806
3		0		1	1ELTW0032	Kiah Creek	11COEHUN	05090102	38.06098	-82.266
3		1		1	1FRLW0001	LEVISA FORK KY	11COEHUN	05070202	37.42494	-82.41214
3		0		1	1JWFW0001	POUND RIVER VA	11COEHUN	05070202	37.23734	-82.34334
3		0		1	1JWFW0002	POUND RIVER VA	11COEHUN	05070202	37.16594	-82.52325
3		0		1	1JWFW0003	CRANES NEST R OF POUND RIVER VA	11COEHUN	05070202	37.12397	-82.4385
3		1		1	1PCSW0002	Paint Creek, Main Inflow to Lake	11COEHUN	05060003	39.32166	-83.3866
3		0		1	1PESW0003	STILLWATER CREEK ABOVE POOL	11COEHUN	05040001	40.10192	-81.14611
3		1		1	1PESW0008	STILLWATER CREEK	11COEHUN	05040001	40.10259	-81.1311
3		0		1	1RDBW0012	Guyandot River	11COEHUN	05070101	37.61083	-81.73417
3		0		1	1RDBW0015	Clear Fork of Guyandot River	11COEHUN	05070101	37.60917	-81.72417
3		0		1	1SUMW0004	MUDDLETY CREEK OF GAULEY RIVER	11COEHUN	05050005	38.32675	-80.83264
3		0		1	1SUTW0008	RIGHT FK HOLLY RIVER WV	11COEHUN	05050007	38.63576	-80.46625
3		1		1	230028	GRAND RIVER AT WEBSTER RD BR; DELTA TWP S	21MICH	04050004	42.76111	-84.64961
3		1		1	233001	Sugar R. at Broadhead	21WIS	07090004	42.55023	-89.3663
3		1		1	243020	Fox R. at Berlin	21WIS	04030201	43.96667	-88.95021
3		1		1	250098	FLINT R AT CARPENTER RD BR; CITY OF FLINT	21MICH	04080204	43.07639	-83.65417
3		1		1	260001	Cuivre River	USGSBRD	07110008	38.92417	-90.74889
3		1		0	263001	MONTREAL RIVER NEAR SAXON, WI	21WIS	04010302	46.55658	-90.41405
3		1		1	273038	BLACK RIVER AT BYPASS CHANNEL @ K	21WIS	07040007	44.38942	-90.76014

Table S1. Description of sites, load data, and calculated loads used in regional nutrient SPARROW models.

Major river basin identifier	If tp_site	If tn_site	Station identifier	Station_name	WQ data program	8-digit cataloging unit	Station latitude	Station longitude	NHDPlus reach identifier	ERF1_2 reach identifier	Major river basin reach identifier	Station upstream area (km2)	DEM-estimated drainage area (km2)	RF1 estimate of mean flow (ft3/s)	Flow station identifier	Daily flow record start date for prediction'Flow station upstream area (km2)	Daily flow record start date for prediction	Daily flow record end date for prediction
3	1	1	303066	Fox (IL) R. near New Munster	21WIS	07120006	42.61085	-88.22541	14769576	21095	90581	2040.8682	2042.13	513.1	05545750	2100.49	10/1/1970	9/30/2006
3	1	1	313038	KEWAUNEE RIVER NEAR KEWAUNEE, WI	21WIS	04030102	44.45745	-87.55573	13064729	11040	90583	343.4081	332.05	77.4	04085200	328.93	10/1/1970	9/30/2006
3	1	1	320540	Iowa River HWY 92 BR AT COLUMBUS JUCTION	21IOWA	07080209	41.27885	-91.34473	11919769	19825	91104	31516.4668	31757.7	9253.4	05465500	32375	10/1/1970	9/30/2006
3	1	0	324015	CEDAR RIVER AT LEWIS ACCESS, 6.5 MI N OF PAL	21IOWA	07080205	42.16135	-91.81074	22473773	19686	91224	15903.7137	16081.93	4759	05464500	16860.9	10/1/1970	9/30/2006
3	1	1	343033	Wolf R. at Langlade	21WIS	04030202	45.18998	-88.73365	9027875	11196	90585	1204.8939	1215.88	401	04074950	1199.17	10/1/1970	9/30/2006
3	1	1	353068	Wisconsin R. at Merril	21WIS	07070002	45.17822	-89.68624	14728798	19260	90586	7122.0338	7199.19	2433	05395000	7148.4	10/1/1970	9/30/2006
3	1	1	373001	WISCONSIN RIVER AT WAUSAU	21WIS	07070002	44.95773	-89.63582	14732266	19254	91428	7829.1038	7985.61	2711	05398000	10411.8	10/1/1970	9/30/2006
3	1	1	373325	BIG EAU PLEINE RIVER AT STRATFORD, WI	USGS	07070002	44.82076	-90.07877	14733228	21139	90591	631.96	576.85	145	05399500	580.16	10/1/1970	9/30/2006
3	1	1	380031x	GRAND R AT MAPLE GROVE RD; RIVES TWP, SEC	21MICH	04050004	42.34167	-84.40163	12246112	11376	91283	932.4	961.01	169.8	04111000	1711.99	10/1/1970	9/30/2006
3	1	1	380153	SHEYENNE RIVER - BALHILL DAM	21NDHDWQ	09020204	47.02735	-98.08619	14824909	38526	91473	9847.4908	11677.52	179.1	05058000	19347.3	10/1/1970	9/30/2006
3	1	1	3822350602001	HOCKING R. DST LANCASTER - 1ST U.S. RT 33 BR	21OHIO	05030204	39.68595	-82.57378	15418191	13338	91020	179.746	173.2	63.8	03157000	230.51	10/1/1970	9/30/2006
3	1	1	383001	PESHTIGO RIVER AT PESHTIGO, WI	21WIS	04030105	45.05381	-87.74706	6819036	11061	90592	2794.61	2884.28	872	04069500	2797.2	10/1/1970	9/30/2006
3	1	1	390057	KALAMAZOO R AT "G" AVE BRIDGE; CITY OF AUG	21MICH	04050003	42.33528	-85.34528	3473567	11352	91276	2356.1489	2391.11	743.5	04105500	2134.16	10/1/1970	9/30/2006
3	1	1	390553	South Skunk River CO RD BR E57	21IOWA	07080105	41.97322	-93.58058	6982264	19538	91182	1480.0037	1516.55	382.2	05470000	815.85	10/1/1970	9/30/2006
3	1	1	390566	SOUTH SKUNK RIVER APPROX 3.5 MI NNW OF CA	21IOWA	07080105	41.95035	-93.55114	6982272	19538	91180	1523.7747	1540.61	388.6	05471000	1440.04	10/1/1970	9/30/2006
3	0	1	391732085414401	CLIFTY CREEK AT CO RD 1150 E NEAR HARTSVILL	USGS	05120206	39.29227	-85.69554	18454477	65600	65600	227.661	234.66	57.5	03364500	236.726	10/1/1970	9/30/2006
3	1	1	394340085524601	SUGAR CREEK AT CO RD 400 S AT NEW PALESTI	USGS	05120204	39.72782	-85.87942	18460036	15657	90594	239.834	270.51	95	03361650	243.201	10/1/1970	9/30/2006
3	1	1	40007	Cannon River	USGSBRD	07040002	44.58477	-92.56284	2028697	18710	90596	3835.1684	3823.44	954.5	05355200	3470.6	10/1/1970	9/30/2006
3	1	1	410050	GRAND RIVER AT KNAPP STREET; ADA TWP, SEC	21MICH	04050006	43.00611	-85.5425	9005295	11425	91328	11577.7144	11658.41	3773.2	04119000	12691	10/1/1970	9/30/2006
3	1	1	410052	GRAND R AT M-11 BRIDGE; CITY OF GRANDVILLE	21MICH	04050006	42.91524	-85.76668	9008449	11420	91325	12790.1711	12924.59	4269.2	04119000	12691	10/1/1970	9/30/2006
3	0	1	413002	MILWAUKEE RIVER @ BROWN DEER ROAD	21WIS	04040003	43.17769	-87.95608	12162670	65154	91329	1632.3993	1680.92	490.7	04087000	1802.64	10/1/1970	9/30/2006
3	1	1	423014	North Raccoon River CO RD BR D46 SEC 13/24 AT S	21IOWA	07100006	42.33971	-94.98585	6608110	20144	91234	1834.5488	1894.48	379.3	05482300	1813	10/1/1970	9/30/2006
3	1	1	426046	South River BRIDGE HIWAY 92	21IOWA	07100008	41.37214	-93.43088	22253729	20199	91108	1230.8198	1226.75	227	05487470	1191.4	10/1/1970	9/30/2006
3	0	1	430380	Des Moines River CO RD E26 NW OF BOONE	21IOWA	07100004	42.08029	-93.93816	6595222	20078	91205	14065.6166	14228.48	3156.7	05481300	14120.68	10/1/1970	9/30/2006
3	1	1	433002	OCONTO RIVER NEAR OCONTO, WI	21WIS	04030104	44.88271	-87.88429	6810610	11051	90599	2504.53	2548.52	677.3	04071765	2501.94	10/1/1988	9/30/2006

Table S1. Description of sites, load data, and calculated loads used in regional nutrient SPARROW models.

Major river basin identifier	tp	lf site	tn	lf site	Station identifier	Station_name	WQ data program	8-digit cataloging unit	Station latitude	Station longitude	NHDPlus reach identifier	ERF1_2 reach identifier	Major river basin reach identifier	Station upstream area (km2)	DEM-estimated drainage area (km2)	RF1 estimate of mean flow (ft3/s)	Flow station identifier
3	1			1	WEL170-0001	E Fk White River	21IND	05120208	38.53917	-87.22306	18446060	15700	90727	14876.9859	14894.85	7247.1	03374000
3	1			1	WEM070-0001	Vernon Fk Muscatatuck Riv	21IND	05120207	38.97639	-85.62	18451107	15694	90729	516.4201	518.35	202.2	03369500
3	1			1	WEU040-0001	E Fk White River	21IND	05120206	38.98722	-85.89889	18455443	15665	90731	6043.8945	6049.74	2869.8	03365500
3	1			1	WLV030-0003	Wabash River	21IND	05120108	40.41194	-87.03639	10213655	15447	90732	19508.1131	19610.99	7566.1	03335500
3	1			1	WLV080-0003	Wabash River	21IND	05120108	40.255	-87.29972	10206599	15443	90734	21085.3713	21079.2	8109.7	03336000
3	1			1	WLV140-0001	Wabash River	21IND	05120108	39.95167	-87.41917	10209941	15442	90735	25311.8369	25296.39	9789.5	03340500
3	1			1	WLV150-0001	Wabash River	21IND	05120108	39.79222	-87.37444	10210063	15436	90736	28903.0791	28877.7	11222.9	03340500
3	1			1	WLV160-0001	Big Raccoon Cr	21IND	05120108	39.79028	-86.95861	10207781	15434	90737	456.5134	453.58	153.9	03340800
3	1			1	WLV190-0012	Big Raccoon Cr	21IND	05120108	39.72917	-87.325	10209489	15433	90738	1229.214	1222.23	462.3	03341300
3	1			1	WLV200-0001	Wabash River	21IND	05120108	39.65639	-87.39556	10210123	15432	90739	30428.1488	30414.98	11732	03340500
3	1			1	WMI060-0004	Mississinewa River	21IND	05120103	40.62806	-85.73583	18503029	15372	90741	1838.6669	1803.49	697.8	03326500
3	1			1	WMI060-0005	Mississinewa River	21IND	05120103	40.57611	-85.65972	18503041	15381	90742	1751.4357	1724.22	667.7	03326500
3	1			1	WMI060-0006	Mississinewa River	21IND	05120103	40.74917	-86.01194	18503223	15372	90743	2103.7534	2068.27	812.2	03326500
3	1			1	WPA040-0003	Patoka River	21IND	05120209	38.32972	-86.96639	18439478	15732	90744	1126.2615	1120.98	545.4	03375500
3	1			1	WPA060-0002	Patoka River	21IND	05120209	38.3825	-87.33333	18440262	15731	90745	1688.0325	1678.67	853.5	03376300
3	1			0	WQN0001	GENESEE RIVER	21PA	04130002	41.9942	-77.8703	15551341	12048	91322	222.0925	221.65	112.2	03010655
3	1			0	WQN0643	CONNEAUT CREEK	21PA	04120101	41.918	-80.4692	9841752	11936	91282	398.1607	380.63	317	04213000
3	1			1	WQN0702	MONONGAHELA RIVER	21PA	05020005	40.1519	-79.9042	3785965	12884	91089	13502.4988	13533.71	9407.5	03075070
3	1			0	WQN0726	CASSELMAN RIVER	21PA	05020006	39.7325	-79.1009	3808779	12950	91058	193.7061	183.65	91.8	03078000
3	1			0	WQN0801	ALLEGHENY RIVER	21PA	05010009	40.52745	-79.84604	11050050	12716	91132	29846.7974	29962.27	20326.2	03049500
3	1			1	WQN0802	ALLEGHENY RIVER	21PA	05010006	40.7611	-79.5458	4742211	12680	91161	23274.3616	23255.8	16824.9	03036500
3	1			0	WQN0807	ALLEGHENY RIVER	21PA	05010001	41.965	-78.3861	8974256	12609	91314	1422.6352	1421.02	849.2	03010500
3	1			0	WQN0843	CLARION RIVER	21PA	05010005	41.1296	-79.5548	6877337	12670	91217	3003.0791	2998.58	1738.1	03030500
3	1			0	WQN0845	FRENCH CREEK	21PA	05010004	41.402	-79.8316	9053513	12657	91240	3187.3576	3181.02	2438.7	03024000
3	1			0	WQN0846	FRENCH CREEK	21PA	05010004	41.5893	-80.1502	9052735	12662	91254	2094.4294	2098.58	1613.5	03023100
3	1			0	WQN0861	MAHONING CREEK	21PA	05010006	40.9221	-79.0064	4740129	12684	91194	411.3697	414.15	255	03034000
3	1			0	WQN0867	ALLEGHENY RIVER	21PA	05010003	41.2638	-79.8415	10222268	12642	91229	16226.1169	16209.08	11936.8	03025500
3	1			0	WQN0869	FRENCH CREEK	21PA	05010004	41.7717	-80.1076	9052233	12663	91273	1542.1896	1527.85	1192.7	03023100
3	1			0	WQN0901	OHIO RIVER	21PA	05030101	40.62058	-80.55885	3821157	12969	91135	60905.2486	60877.43	40409.8	03086000
3	1			0	WQN0902	OHIO RIVER	21PA	05030101	40.533	-80.1881	3821227	12973	91124	50462.4463	50447.52	35112.2	03086000
3	1			1	WSA020-0002	Salamonie River	21IND	05120102	40.55917	-85.27861	18505908	15371	90746	657.7046	629.65	215.4	03324300
3	1			1	WSA040-0001	Salamonie River	21IND	05120102	40.82972	-85.71889	18506684	15371	90747	1452.6533	1424.82	534.4	03324300
3	1			1	WSA040-0005	Salamonie River	21IND	05120102	40.74167	-85.50889	18505952	15371	90748	1154.4925	1126.18	408.5	03324300
3	1			1	WSU020-0003	Sugar Cr	21IND	05120110	40.14333	-86.69583	18482194	15470	90749	749.6755	681.71	322.2	03339500

[illegible]

Table S1. Description of sites, load data, and calculated loads used in regional nutrient SPARROW models.

Major river basin identifier	tp lf site	tn lf site	Station identifier	Station_name	WQ data program	8-digit cataloging unit	Station latitude	Station longitude	NHDPlus reach identifier	ERF1_2 reach identifier	Major river basin reach identifier
4	1	1	06892350	KANSAS RIVER AT DE SOTO	USGS	10270104	38.98	-94.96	3730399	65665	65665
4	1	1	06893500	Blue River at Kansas City, MO	USGS	10300101	38.95	-94.55	4392705	26302	91544
4	1	1	06893552	BLUE RIVER STATION 40 (BR-40)	USGS	10300101	39.03	-94.52	4390829	26302	91821
4	1	1	06893590	Blue River at 12th Street in Kansas City, MO	USGS	10300101	39.09	-94.49	4391195	26302	91565
4	1	1	06898100	Thompson River at Mount Moriah, MO	USGS	10280102	40.33	-93.76	5140843	25845	91614
4	1	1	06902000	Grand River near Sumner, MO	USGS	10280103	39.64	-93.27	5969716	65275	65275
4	1	1	06905500	Chariton River near Prairie Hill, MO	USGS	10280202	39.53	-92.79	5816473	65276	65276
4	1	1	06906300	East Fork Little Chariton R. near Huntsville, MO	USGS	10280203	39.45	-92.56	4462574	25948	91579
4	1	1	06907300	Lamine River near Pilot Grove, MO	USGS	10300103	38.89	-93.03	5996890	26382	91540
4	1	1	06911900	DRAGON C 2.25MI S/BURLINGAME,KAN	USGS	10290101	38.71	-95.83	10117754	26532	91527
4	1	1	06918070	Osage River above Schell City, MO	USGS	10290105	38.05	-94.14	2985390	65277	65277
4	1	0	06918440	Sac River near Dadeville, MO	USGS	10290106	37.44	-93.68	7374295	26730	91509
4	1	1	06918600	LITTLE SAC RIVER NEAR WALNUT GROVE, MO	USGS	10290106	37.39	-93.41	7374611	26106	91508
4	1	1	06921070	POMME DE TERRE RIVER	USGS	10290107	37.68	-93.37	7388043	26448	91510
4	1	1	06926510	Osage River below St. Thomas, MO	USGS	10290111	38.42	-92.2	5987239	65278	65278
4	1	1	06930450	Big Piney River at Devils Elbow, MO	USGS	10290202	37.84	-92.06	7434891	26241	91514
4	1	1	06930800	Gasconade River above Jerome, MO	USGS	10290203	37.91	-91.97	7447479	26262	65632
4	1	1	06934500	Missouri River at Hermann, MO	USGS	10300200	38.7	-91.43	6013072	65279	65279
4	1	1	06935890	Creve Coeur Creek near Creve Coeur, MO	USGS	10300200	38.68	-90.48	6009374	26439	91856
4	1	1	06935955	Fee Fee Creek near Bridgeton, MO	USGS	10300200	38.72	-90.44	6009232	26440	91533
4	1	1	06935980	Cowmire Creek at Bridgeton, MO	USGS	10300200	38.76	-90.43	6010012	26441	92191
4	1	1	06936475	Coldwater Creek near Black Jack, MO	USGS	10300200	38.81	-90.25	6010004	26443	91542
4	1	1	080000	BEAR CREEK ABOVE HARRIMAN CANAL		10190002	39.65	-105.18	188633	23470	91604
4	1	1	08CHI2	PLUM CREEK ABOVE CHATFIELD LAKE		10190002	39.51	-105.02	188691	23537	91590
4	1	1	10040001	Chariton River near Centerville		10280201	40.79	-92.85	5121408	25912	91626
4	1	1	10180001	Little Sioux River near Larrabee		10230003	42.86	-95.46	14622326	24369	91651
4	1	1	10210003	1.7 MILES DWNSTRM SPENCER STP		10230003	43.13	-95.11	14620538	24389	91665
4	1	1	10270001	HWY 69 BR AT USGS GAGE DAVIS CITY		10280102	40.64	-93.8	5138325	25848	91617
4	1	1	10430001	Boyer River near Missouri Valley		10230007	41.57	-95.84	17208720	24495	91637
4	1	1	10430002	CO RD BR F-20 NR PISGAH		10230001	41.83	-95.93	17246982	24310	91640
4	1	1	10650001	WEST NISHNABOTNA RIVER; 3 MI EAST OF MALVERN		10240002	40.98	-95.52	19210295	24547	91633
4	1	1	10670002	Maple River near Mapleton		10230005	42.17	-95.79	17226997	24450	91641
4	1	1	10840001	ROCK RIVER, 6 MILES NORTH OF HAWARDEN, IOWA		10170204	43.08	-96.44	7242977	28834	91660
4	1	1	10970001	Little Sioux River near Smithland		10230003	42.25	-95.9	14625332	24357	91643
4	1	1	180418	BIG BULL CK AT I-35		10290102	38.75	-94.97	2993494	26030	91530
4	1	1	226_184_7_0	Missouri R. @ Nebraska City, NE		10240001	40.68	-95.84	7312363	65267	65267
4	1	1	280430	LINDLEY CREEK MO HWY 64 BRIDGE		10290107	37.75	-93.28	7387709	26123	91511
4	1	1	280509	POM DE TER R -MO.HWY Y HT-9		10290107	37.98	-93.31	7388781	26450	91517
4	1	0	280518	S GRAND R -HENRY CO HWY K HT-18		10290108	38.45	-94	2335475	26469	91521
4	1	0	280525	SAC R -MO HWY 54		10290106	37.89	-93.75	7373017	26073	91515
4	1	0	280541	LITTLE OSAGE R - VERNON CO RD V		10290103	37.98	-94.56	7362815	26039	91516
4	1	1	280624	TURNBACK CREEK-HWY O BRIDGE SE OF GREENFIELD,MO.		10290106	37.4	-93.8	7374577	26731	91507
4	1	1	380013	JAMES RIVER - JAMESTOWN		10160003	46.88	-98.67	11463132	28407	91794
4	1	1	380015	PIPESTEM INFLOW SITE 5		10160002	47.06	-98.91	11500144	28375	91796
4	1	1	380022	LITTLE MISSOURI RIVER - MEDORA		10110203	46.91	-103.53	14567692	34499	91797
4	1	1	380058	BEAVER CREEK - S OF LINTON		10130104	46.25	-100.23	14665399	27379	91779
4	1	1	380059	LITTLE MISSOURI RIVER - HWY 85		10110205	47.59	-103.25	14469321	65239	65239
4	1	1	380060	SPRING CREEK - S OF ZAP		10130201	47.27	-101.86	16248829	27412	91802
4	1	1	380152	PIPESTEM CREEK - W OF PINGREE		10160002	47.16	-98.96	11500082	28375	91798
4	1	1	384056	BEAVER CREEK		10130104	46.26	-100.25	14665387	27378	91781
4	1	1	384058	BEAVER CREEK		10130104	46.26	-100.39	14665133	29025	91780
4	1	1	384183	CEDAR CREEK		10130205	46.22	-102.68	21859950	27562	91783
4	1	1	384212	PIPESTEM CREEK		10160002	47.33	-99.14	11499118	28376	91800
4	1	0	384215	BEAR CREEK		10160003	46.22	-98.07	11468868	28393	91777
4	1	0	385026	MAPLE RIVER		10160004	46.15	-98.43	11440827	28430	91771
4	1	1	391557095531100	SOLDIER C, I RD NR DELIA, KS SC-7	USGS	10270102	39.26	-95.88	3645494	25494	91571
4	1	0	402114105350101	Big Thompson R. in Moraine Park at Old NPS Gage	USGS	10190006	40.35	-105.58	13584	0	91859
4	1	0	460039	WQM 39 - MOREAU RIVER NEAR USTA.		10130306	45.19	-102.15	11252528	27777	91749
4	1	1	460113	WQM 113 - James River near Columbia		10160003	45.66	-98.3	11473592	29183	29183
4	1	1	460117	WQM 117 - BIG SIOUX RIVER NEAR SIOUX FALLS.		10170203	43.6	-96.63	7223418	28793	91676
4	1	0	460121	SKUNK CREEK WEST OF SIOUX FALLS		10170203	43.53	-96.79	7223720	28815	91670
4	1	0	460122	WQM 122 - WHITEWOOD CREEK NEAR DEADWOOD.		10120202	44.35	-103.73	5458062	27282	91721
4	1	0	460123	WQM 123 - WHITEWOOD CREEK NEAR DEADWOOD.		10120202	44.38	-103.72	5457948	27282	91723
4	1	0	460126	WQM 126 - BEAR BUTTE CREEK ABOVE GALENA		10120202	44.32	-103.64	5458150	27285	91716
4	1	0	460132	WQM 132 - Cheyenne River near Red Shirt		10120109	43.67	-102.89	14399328	26990	26990
4	0	1	460133	WQM 133 - Cheyenne River at Highway 63 crossing		10130105	44.69	-101.21	19246633	29102	91732
4	1	0	460136	WQM 136 - Elm River at Highway 281 crossing		10160004	45.66	-98.51	11446173	28428	91757
4	1	0	460137	WQM 137 - Firesteel Creek near Mount Vernon		10160011	43.77	-98.26	12664828	28604	91686
4	1	0	460140	WQM 140 - James River at Highway 212 crossing		10160006	44.87	-98.33	12695142	28507	91737
4	0	1	460152	WQM 152 - White River at Highway 83 Crossing		10140202	43.71	-100.68	16076140	28053	91689
4	1	0	460646	WQM 46 - CASTLE CREEK NEAR MYSTIC		10120110	44.07	-103.64	17533199	27023	91711
4	1	0	460649	WQM 49 - SPRING CREEK NEAR RAPID CITY.		10120109	43.98	-103.4	14395752	28890	91706
4	1	0	460650	WQM 50 - GRACE COOLIDGE CREEK NEAR GAME LODGE		10120109	43.76	-103.36	14396556	26987	91695
4	1	0	460651	WQM 51 - FRENCH CREEK NEAR BLUEBELL LODGE		10120109	43.71	-103.47	14396676	0	91860
4	1	0	460652	WQM 52 - WHITEWOOD CREEK NEAR WHITEWOOD.		10120202	44.47	-103.62	5457710	27282	91727
4	1	0	460654	WQM 54 - SPRING CREEK NEAR SHERIDAN LAKE.		10120109	43.96	-103.48	14397536	26977	91704
4	1	0	460657	WQM 57 - FALL RIVER NEAR THE CONFLUENCE WITH CHEYE		10120109	43.4	-103.41	14400448	27007	91677
4	1	1	460666	WQM 66 - BIG SIOUX RIVER NEAR HUDSON.		10170203	43.13	-96.44	7226200	28769	91664
4	1	1	460667	WQM 67 - BIG SIOUX RIVER NEAR ALCESTER.		10170203	42.99	-96.49	7226072	28767	91656
4	1	0	460669	WQM 69 - RAPID CREEK NEAR RAPID CITY.		10120110	44.04	-103.32	17533267	27017	91709
4	1	0	460674	WQM 74 - MISSOURI RIVER AT GAVINS POINT DAM		10170101	42.85	-97.48	11761582	29225	91650
4	1	0	460676	WQM 76 - BELLE FOURCHE RIVER NORTHWEST OF ELM SPRI		10120202	44.36	-102.54	5460192	27215	91715
4	1	0	460678	WQM 78 - SOUTH FORK GRAND RIVER NEAR BISON.		10130302	45.61	-102.45	16187400	27605	91761
4	1	0	460685	WQM 85 - WHITEWOOD CREEK NEAR DEADWOOD.		10120202	44.38	-103.7	5457894	27282	91724
4	1	0	460686	WQM 86 - WHITEWOOD CREEK NEAR PLUMA.		10120202	44.35	-103.73	5458062	27282	91722
4	1	0	460707	WQM 7 - JAMES RIVER NEAR MITCHELL		10160011	43.65	-97.91	12667026	28589	91679
4	1	0	460734	WQM 34 - JAMES RIVER NEAR STRATFORD		10160003	45.34	-98.33	11479028	28382	91750
4	1	0	460737	WQM 37- JAMES RIVER NEAR MITCHELL.		10160011	43.7	-97.96	12666952	28590	91682
4	1	1	460761	WQM 8 - JAMES RIVER NEAR YANKTON		10160011	42.99	-97.36	12669690	28574	91657
4	1	0	460815	WQM 10 - KEYA PAHA RIVER NEAR WEWELA		10150006	43.02	-99.77	11654348	28293	91659
4	1	1	460825	WQM 12 - WHITE RIVER NEAR OACOMA		10140204	43.74	-99.54	11547654	28114	91684
4	1	1	460831	WQM 31 - BIG SIOUX RIVER NEAR BRANDON.		10170203	43.59	-96.59	7223530	28793	91675
4	1	1	460832	WQM 32 - BIG SIOUX RIVER NEAR RICHLAND.		10170203	42.76	-96.63	7226684	28760	91648
4	1	1	460840	WQM 13 - LITTLE WHITE RIVER NEAR WHITE RIVER		10140203	43.6	-100.74	20177973	28087	91680
4	1	0	460842	WQM 42 - WHITE RIVER NEAR OGLALA.		10140201	43.18	-102.8	16098241	27995	91668
4	1	0	460865	WQM 15 - CHEYENNE RIVER NEAR WASTA		10120111	44.08	-102.4	14555097	27037	91708
4	1	0	460875	WQM 14 - CHEYENE RIVER NEAR EDGEMONT		10120106	43.3	-103.82	9555429	26904	91671
4	1	0	460880	WQM 21 - BELLE FOURCHE RIVER NEAR VOLUNTEER.		10120202	44.51	-103.13	5454116	27243	91729
4	1	0	460895	WQM 23 - REDWATER RIVER NEAR BELLE FOURCHE		10120203	44.66	-103.83	5479729	27293	91736
4	1	0	460900	WQM 22 - SPEARFISH CREEK NEAR SPEARFISH.		10120203	44.48	-103.86	5481765	27310	91730
4	1	0	460905	WQM 17 - BATTLE CREEK NEAR HAYWARD.		10120109	43.87	-103.33	14396112	26984	91697
4	1	0	460925	WQM 30 - BOX ELDER CREEK NEAR NEMO.		10120111	44.14	-103.45	14553227	27042	91713
4	1	1	468860	WQM 16 - CHEYENNE RIVER NEAR BRIDGER.		10120112	44.52	-101.99	14376633	27067	91725
4	1	1	46BS18	WQM BS18 - BIG SIOUX RIVER NORTH OF FLANDREAU		10170203	44.07	-96.58	7219270	28808	91698
4	1	1	46BS29	WQM BS29 - BIG SIOUX RIVER RIVER NEAR SIOUX FALLS.		10170203	43.57	-96.68	7223598	28795	91673
4	1	0	46MN32	WQM MN32 - SPEARFISH CREEK NEAR ELMORE.		10120203	44.32	-103.89	5481937	27312	91718
4	1	0	46MN33	WQM MN33 - SPEARFISH CREEK NEAR ELMORE.		10120203	44.33	-103.89	5481925	27312	91719
4	1	0	46MN34	WQM MN34 - SPEARFISH CREEK NEAR ELMORE.		10120203	44.33	-103.9	5481921	27312	91720
4	1	1	682_59_0	E. Fk. Chariton near Hwy 36		10280203	39.74	-92.51	4461478	25948	91587
4	1	1	696_6_6	Long Br. @ Inkwell St.		10280203	39.89	-92.49	4461060	25949	91599
4	1	1	821008	EAST NISHNABOTNA R AT HWY 59 BR N OF SHENANDOAH		10240003	40.78	-95.38	19167057	24577	91625
4	1	1	950110	CO RD BR 3 MI N OF SIOUX CITY		10230002	42.54	-96.33	17240020	24343	91645
4	1	1	BDC	Big Dry Creek 50 yards upstream USGS Guage Station		10190003	40.06	-104.83	225687	23565	91822
4	1	1	L01	South Platte River Upstream		10190002	39.56	-105.04	188319	65258	91593
4	1	1	L04	South Platte River at Mineral		10190002	39.58	-105.03	188305	65258	91595
4	1	0	S000-097	ROCK RIVER BR ON STATELINE RD 10 MI S OF LUVERNE		10170204	43.5	-96.18	7240989	28858	91669
5	1	0	000008	ARKANSAS RIVER NEAR SALIDA		11020001	38.6333333	-106.0666667	918151	35182	92782
5	1	0	000009	ARKANSAS R. BELOW LEADVILLE, COL		11020001	39.2572222	-106.3430556	916811	35193	92790
5	1	1	000015	FOUNTAIN CR. @ PUEBLO		11020003	38.2708889	-104.6003611	1532253	35256	92774
5	1	0	000574	OTTER CREEK NEAR CLIMAX		11070102	37.708626	-96.223617	21517052	35967	92745
5	1	1	000597	WALNUT CREEK AT GREAT BEND		11030008	38.42	-98.847778	21201294	35490	92769
5	1	1	0116	Tickfaw River at Springville, Louisiana		08070203	30.43527778	-90.6775	20091282	22380	92393
5	1	1	0119	Amite River at Grangeville, Louisiana		08070202	30.73611111	-90.84166667	18996374	22345	92411
5	1	1	050021	LITTLE RIVER NEAR HORATIO ARK		11140109	33.919444	-94.3875	3750770	37583	92553
5	1	0	050152	DORCHEAT BAYOU EAST OF TAYLOR, AR		11140203	33.098056	-93.386111	19934169	37623	92523
5	1	0	050198	WHITE RIVER ABOVE JACKSONPORT ARK		11010013	35.639444	-91.315278	11799705	35140	92643
5	1	1	0553	Saline Bayou at bridge on Forest 507 near Goldonna, Louisiana		11140208	32.06333333	-92.90194444	19913410	37974	92457
5	1	1	07024000	BAYOU DE CHIEN NEAR CLINTON, KY	USGS	08010201	36.62867072	-88.96395098	14126587	21328	92705
5	1	1	07029500	HATCHIE RIVER AT BOLIVAR, TN	USGS	08010208	35.27532938	-88.97663034	14078174	65284	65284
5	1	1	07030392	WOLF R 72.6 @ HWY 8113 NEAR LAGRANGE QUA	USGS	08010210	35.032222	-89.2475	14208484	21567	92618
5	1	1	07031692	FLETCHER CREEK AT SYCAMORE VIEW ROAD AT	USGS	08010210	35.16936978	-89.86612539	14206348	21574	92622
5	1	1	07047942	LAnguille River near Colt, AR	USGS	08020205	35.14453417	-90.87844889	22860477	21658	92621
5	1	1	07048600	White River near Fayetteville, AR	USGS	11010001	36.07313417	-94.08131278	8590894	34852	92657
5	1	1	07048700	White River near Goshen, AR	USGS	11010001	36.10618861	-94.01158833	8590362	34852	92665
5	1	1	07048800	Richland Creek at Goshen, AR	USGS	11010001	36.10415833	-94.00758333	8590392	34858	92664
5	1	1	07049000	War Eagle Creek near Hindsville, AR	USGS	11010001	36.200075	-93.85519556	8588002	34863	92673
5	1	1	07050500	Kings River near Berryville, AR	USGS	11010001	36.42599167	-93.62319167	8586784	34865	65538
5	1	1	07051600	James River near Wilson Creek, MO	USGS	11010002	37.07644165	-93.37102299	7608069	37836	92722
5	1	1	07052152	Wilson Creek near Brookline, MO	USGS	11010002	37.14713611	-93.37546111	7607249	34890	92728
5	1	1	07052160	Wilson Creek nr Battlefield MO	USGS	11010002	37.11775833	-93.40386667	7607329	34890	92726
5	1	1	07052250	James River near Boaz, MO	USGS	11010002	37.00657222	-93.36467778	7607905	34884	92715
5	1	1	07052345	Finley Creek below Riverdale, MO	USGS	11010002	36.9749	-93.32788056	7608695	34883	92711
5	1	1	07052500	James River at Galena, MO	USGS	11010002	36.80539167	-93.461575	7609749	34882	92704
5	1	0	07053230	LONG CREEK BELOW DENVER ARK	USGS	11010001	36.429583	-93.306111	8589260	37853	92691
5	1	1	07053250	Yocum Creek near Oak Grove, AR	USGS	11010001	36.45395528	-93.3565725	8586574	37852	65539
5	1	1	07055646	BUFFALO RIVER AT WILDERNESS BOUNDARY	USGS	11010005	35.943056	-93.405556	11819355	65540	65540
5	1	1	07056000	Buffalo River near St. Joe, AR	USGS	11010005	35.983175	-92.74755278	11818755	65541	65541
5	1	1	07057500	North Fork River near Tecumseh, MO	USGS	11010006	36.62303333	-92.248125	7650991	34964	92700
5	1	0	07058500	NORTH FORK RIVER AT TECUMSEH	USGS	11010006	36.60531667	-92.27958611	7651767	34964	92698
5	1	1	07060500	White River at Calico Rock, AR	USGS	11010004	36.11618111	-92.14320917	11830684	34918	92669
5	1	1	07060710	NORTH SYLAMORE CREEK NR FIFTYSIX AR.	USGS	11010004	35.99173948	-92.21404354	11834118	65288	65288
5	1	0	07061105	WHITE RIVER AT OIL TROUGH, AR.	USGS	11010004	35.64341194	-91.4618	11833792	34922	92642
5	1	1	07061600	Black River below Annapolis, MO	USGS	11010007	37.32504888	-90.76400933	7668920	65542	65542
5	1	0	07064000	BLACK RIVER NEAR CORNING, ARK.	USGS	11010007	36.40200525	-90.54149892	7672652	34991	92692
5	1	1	07066000	Jacks Fork at Eminence, MO	USGS	11010008	37.15408056	-91.35816111	7518797	35058	92732
5	1	1	07066110	Jacks Fork above Two River, MO	USGS	11010008	37.17282601	-91.30013335	7518555	35051	92735
5	1	1	07068000	Current River at Doniphan, MO	USGS	11010008	36.62200294	-90.84762251	7523285	35027	92701
5	1	1	07071500	ELEVEN POINT RIVER NEAR BARDLEY	USGS	11010011	36.648611	-91.200833	7572703	35096	92702
5	1	0	07074100	STRAWBERRY RIVER NR SMITHVILLE, ARK.	USGS	11010012	36.027778	-91.325278	11808624	35113	92663
5	1	1	07077000	White River at DeValls Bluff, AR	USGS	08020301	34.79037167	-91.44596278	25189323	21664	92597
5	1	1	07077380	Cache River at Egypt, AR	USGS	08020302	35.85785222	-90.9334525	9273022	21698	92653
5	1	1	07077500	Cache River at Patterson, AR	USGS	08020302	35.26953083	-91.23762556	9277778	21694	92625
5	1	1	07077555	Cache River near Cotton Plant, AR	USGS	08020302	35.03536861	-91.32207194	9278434	21693	92614
5	1	1	07077700	Bayou DeView near Morton, AR	USGS	08020302	35.25203139	-91.1104	9280092	21681	92623
5	0	1	07083000	HALFMOON CREEK NEAR MALTA, CO.	USGS	11020001	39.17221259	-106.3891926	916821	35194	65291
5	1	1	07097000	ARKANSAS RIVER AT PORTLAND, CO.	USGS	11020002	38.38833418	-105.0160961	949993	35236	65292
5	1	1	07099400	ARKANSAS RIVER ABOVE PUEBLO, CO.	USGS	11020002	38.27166941	-104.718033	950993	35232	92775
5	1	1	07103780	MONUMENT C AB N.GATE BLVD AT USAF ACADEM	USGS	11020003	39.03110354	-104.8483128	1528197	35262	92788
5	1	0	07103970	MONUMENT CR ABV WOODMEN RD AT COLORAD	USGS	11020003	38.9338817	-104.8171992	1529541	35265	92787
5	0	1	07104000	MONUMENT CREEK AT PIKEVIEW, CO.	USGS	11020003	38.91777057	-104.8185877	1529569	35260	92786
5	0	1	07105500	FOUNTAIN CREEK AT COLORADO SPRINGS, CO.	USGS	11020003	38.81638158	-104.8227519	1529751	35258	92784
5	1	1	07105530	FOUNTAIN CR BL JANITELL RD BL COLO. SPRINGS	USGS	11020003	38.80304911	-104.7958069	1529807	35258	92783
5	1	0	07105800	FOUNTAIN CREEK AT SECURITY, CO.	USGS	11020003	38.7294404	-104.7338611	1529977	35258	92781
5	0	1	07105905	FOUNTAIN C AB L FOUNTAIN CR, BL FOUNTAIN, CO	USGS	11020003	38.63055318	-104.6810836	1530431	35257	92780
5	1	0	07106000	FOUNTAIN CREEK NEAR FOUNTAIN, CO.	USGS	11020003	38.6016647	-104.6702503	1530529	35258	92779
5	1	1	07106300	FOUNTAIN CREEK NEAR PINON, CO.	USGS	11020003	38.42944444	-104.5980556	1531919	35257	92778
5	1	1	07106500	FOUNTAIN CREEK AT PUEBLO, CO.	USGS	11020003	38.28778013	-104.6010849	1532253	35256	92776
5	1	1	07109500	ARKANSAS RIVER NEAR AVONDALE, CO.	USGS	11020002	38.24805799	-104.3991356	952589	35227	92772
5	1	1	07124000	ARKANSAS RIVER AT LAS ANIMAS, CO.	USGS	11020009	38.08083994	-103.2196523	1594283	35333	92765
5	1	1	07128500	PURGATOIRE RIVER NEAR LAS ANIMAS, CO.	USGS	11020010	38.0338961	-103.2004854	933650	35339	92762
5	0	1	07140000	ARKANSAS RIVER NEAR KINSLEY	USGS	11030004	37.927778	-99.375278	22084115	35463	92754
5	1	1	07143665	LITTLE ARKANSAS RIVER AT ALTA MILLS	USGS	11030012	38.11244	-97.59198	21176042	35530	92758
5	1	0	07143672	L ARKANSAS R AT HWY 50 NR HALSTEAD, KS	USGS	11030012	38.02862234	-97.54059705	21176300	35529	92755
5	1	0	07144100	L ARKANSAS R NR SEDGWICK, KS	USGS	11030012	37.88306739	-97.42448639	21177172	35523	92752
5	1	1	07144601	NF NINNESCAH R AT ARLINGTON, KS	USGS	11030014	37.89626956	-98.17110699	21159287	35554	92753
5	1	0	07144660	SILVER C NR ARLINGTON, KS	USGS	11030014	37.84279255	-98.19645308	21159421	35555	92750
5	1	1	07144780	NF NINNESCAH R AB CHENEY RE, KS	USGS	11030014	37.86260737	-98.01385325	21159337	38277	92751
5	1	1	07144795	NF NINNESCAH R AT CHENEY DAM, KS	USGS	11030014	37.72140097	-97.79449556	21160795	38278	92746
5	1	1	07145200	SOUTH FORK NINNESCAH RIVER NEAR MURDOCK	USGS	11030015	37.56176	-97.85362	21152052	35558	92743
5	1	1	07146500	Arkansas River Near Arkansas City	USGS	11030013	37.05641	-97.05897	21166925	35535	65294
5	1	1	07147070	WHITEWATER RIVER AT TOWANDA	USGS	11030017	37.79591	-97.01305	20078342	35584	92747
5	1	1	07154500	Cimarron River near Kenton, OK	USGS	11040001	36.92668694	-102.9590956	21126643	35617	92725
5	1	1	07159100	CIMARRON RIVER NEAR DOVER	USGS	11050002	35.951667	-97.914167	255558	35750	92654
5	1	1	07164500	Arkansas River at Tulsa, OK	USGS	11110101	36.14064806	-96.00638667	369211	36694	65297
5	1	0	07176000	Verdigris River near Claremore, OK	USGS	11070105	36.30731861	-95.69803722	833464	36024	92680
5	1	1	07178200	Bird Ck at State Highway 266 near Catoosa, OK	USGS	11070107	36.22315139	-95.81943222	847450	36054	92674
5	1	1	07185000	Neosho River near Commerce, OK	USGS	11070206	36.92868139	-94.9574575	24778185	36129	92707
5	1	1	07186000	Spring River near Waco, MO	USGS	11070207	37.24560556	-94.56641111	7590909	36162	92733
5	1	1	07188000	Spring River near Quapaw, OK	USGS	11070207	36.93451139	-94.74717111	7592691	36154	92708
5	1	1	07189000	Elk River near Tiff City, Mo	USGS	11070208	36.63146139	-94.58688861	7601075	36175	65544
5	1	0	07191000	Big Cabin Creek near Big Cabin, OK	USGS	11070209	36.56841778	-95.15218917	21770039	36196	92693
5	1	1	07191160	Spavinaw Creek near Maysville, AR	USGS	11070209	36.3645225	-94.55133	21770953	36191	92688
5	1	1	07191179	Spavinaw Creek near Cherokee, AR	USGS	11070209	36.34202361	-94.58772	21771041	36191	92685
5	1	1	07191220	Spavinaw Creek near Sycamore, OK	USGS	11070209	36.33472222	-94.64138889	21771103	36191	92683
5	1	1	071912213	Spavinaw Creek near Colcord, OK	USGS	11070209	36.32258083	-94.68522389	21771121	36190	92682
5	1	1	07191222	Beaty Creek: Lower	USGS	11070209	36.366	-94.727	21770925	36192	92687
5	1	1	07194800	Illinois River at Savoy, AR	USGS	11110103	36.10313556	-94.3443775	398478	36737	92661
5	1	1	07194830	Illinois River near Pedro, Ark	USGS	11110103	36.17563528	-94.39187917	397926	36736	92670
5	1	1	07195000	Osage Creek near Elm Springs, AR	USGS	11110103	36.22206944	-94.28658056	399500	36743	92676
5	1	1	07195430	ILLINOIS RIVER SOUTH OF SILOAM SPRINGS ARK	USGS	11110103	36.108611	-94.533333	398430	36733	92662
5	1	1	07195855	FLINT CREEK 1/8 MILE BELOW FAGEN CREEK	USGS	11110103	36.216278	-94.604167	397590	36744	92802
5	1	1	07196000	Flint Creek near Kansas, OK	USGS	11110103	36.1864725	-94.70689139	399514	36744	92671
5	1	1	07196090	ILLINOIS RIVER AT CHEWEY BRIDGE	USGS	11110103	36.104083	-94.78275	398442	65545	92660
5	1	1	07196500	Illinois River near Tahlequah, OK	USGS	11110103	35.92286889	-94.92356583	401664	36732	65545
5	1	1	07196520	Illinois River nr Park Hill, OK	USGS	11110103	35.85314694	-94.91550917	401900	36732	92647
5	1	1	07196900	Baron Fork at Dutch Mills, AR	USGS	11110103	35.88008222	-94.48660639	400822	36730	92648
5	1	1	07197360	Caney Creek near Barber, OK	USGS	11110103	35.78481278	-94.85606194	401366	37932	92644
5	1	1	07198000	Illinois River near Gore, OK	USGS	11110103	35.57315111	-95.06884583	402478	36726	92638
5	0	1	07211500	CANADIAN RIVER NEAR TAYLOR SPRINGS, NM	USGS	11080003	36.29697667	-104.4938739	20050371	36233	92694
5	1	1	07221500	CANADIAN RIVER NEAR SANCHEZ, NM	USGS	11080003	35.65226417	-104.3780369	20052023	36227	65300
5	1	1	07227500	Canadian Rv nr Amarillo, TX	USGS	11090105	35.47032612	-101.8796281	19984864	36348	92639
5	1	1	07228000	Canadian River near Canadian, TX	USGS	11090106	35.93504194	-100.3706883	19972126	65302	65302
5	1	1	07231500	Canadian River at Calvin, OK	USGS	11090202	34.97786694	-96.24361722	6030344	36429	65303
5	1	0	07235000	Wolf Creek at Lipscomb, TX	USGS	11100203	36.23864889	-100.2756889	13812194	36587	92681
5	1	1	07237500	North Canadian River at Woodward, OK	USGS	11100301	36.43670333	-99.27843833	388800	36522	65305
5	1	1	07239450	North Canadian River near Calumet, OK	USGS	11100301	35.61699333	-98.06533167	391180	36602	92640
5	1	1	07241000	North Canadian River blw Lk Overholser nr OKC, OK	USGS	11100301	35.47866861	-97.66337528	393414	36599	92630
5	1	1	07241550	North Canadian River near Harrah, OK	USGS	11100302	35.50034111	-97.19391944	414203	36625	92631
5	1	1	07242000	North Canadian River near Wetumka, OK	USGS	11100302	35.26564556	-96.20611667	418429	65306	65306
5	1	1	07243500	Deep Fork near Beggs, OK	USGS	11100303	35.6739875	-96.06860778	536835	36645	65307
5	1	1	07247000	Poteau River at Cauthron, AR	USGS	11110105	34.91898861	-94.298825	6048196	36799	92604
5	1	1	07247015	Poteau River: Loving	USGS	11110105	34.8797222	-94.484	6047872	36799	92598
5	1	1	07247250	Black Fork below Big Creek nr Page, OK	USGS	11110105	34.77371472	-94.51216861	6048082	36797	92592
5	1	1	07247345	Black Fork at Hodgen, OK	USGS	11110105	34.84315722	-94.62467056	6056044	36796	92596
5	1	1	07247500	Fourche Maline near Red Oak, OK	USGS	11110105	34.91259917	-95.15579861	6049306	36791	92601
5	1	1	07247650	Fourche Maline near Leflore, OK	USGS	11110105	34.91982139	-94.9455125	6047750	36794	92603
5	1	1	07249400	James Fork near Hackett, AR	USGS	11110105	35.16259583	-94.40716083	6041516	36805	92616
5	1	0	07249440	POTEAU RIVER NR FORT SMITH ARK	USGS	11110105	35.345278	-94.4525	6041364	36781	92624
5	1	1	07249455	Arkansas River at Ft. Smith, Ar.	USGS	11110104	35.39175778	-94.43243722	1543497	36757	92627
5	0	1	07249985	Lee Creek near Short, OK	USGS	11110104	35.51925472	-94.46632639	1539671	36746	92633
5	1	0	07261500	Fourche LaFave River Near Gravelly, AR	USGS	11110206	34.87259472	-93.6568625	7830338	36914	92599
5	1	1	07263295	Maumelle River at Williams Junction, AR	USGS	11110207	34.87592278	-92.77461167	22846153	38039	92602
5	1	1	07263450	ARK RIVER AT MURRAY LOCK AND DAM	USGS	11110207	34.790833	-92.358889	22848095	36933	92595
5	1	1	07263620	ARKANSAS R @ DAVID D TERRY L&D BL LITTLE R	USGS	11110207	34.66720556	-92.15714167	22848177	65771	65771
5	1	1	07268000	LITTLE TALLAHATCHIE RIVER AT ETTA, MS	USGS	08030201	34.4825	-89.22444444	15288074	21783	92589
5	1	0	07273100	HOTOPHA CREEK NR BATESVILLE, MS	USGS	08030201	34.36388889	-89.87861111	15289204	21775	92585
5	1	0	07274252	OTOUCALOFA CREEK CANAL NR WATER VALLEY,	USGS	08030203	34.14416667	-89.6525	15275898	22734	92578
5	1	0	07277700	HICKAHALA CREEK NR SENATOBIA, MS	USGS	08030204	34.63194444	-89.92444444	15251676	21851	92593
5	1	1	07283000	SKUNA RIVER AT BRUCE, MS	USGS	08030205	33.97361111	-89.34777778	18015234	22849	92571
5	1	0	07287405	BLACK CREEK AT HOWARD, MS	USGS	08030206	33.11957152	-90.19119704	17998628	21907	92525
5	1	1	07288650	BOGUE PHALIA NR LELAND, MS	USGS	08030207	33.39666667	-90.84777778	17963095	21932	92535
5	1	1	07288955	YAZOO RIVER BL STEELE BAYOU NR LONG LAKE,	USGS	08030208	32.44416667	-90.91416667	17948636	21936	92478
5	1	1	07289350	BIG BLACK RIVER At WEST AT HWY 19	USGS	08060201	33.19447222	-89.77116667	19222549	22213	92530
5	1	1	07290000	BIG BLACK RIVER NR BOVINA, MS	USGS	08060202	32.34777778	-90.69694444	19187384	65311	65311
5	1	1	07290650	BAYOU PIERRE NR WILLOWS, MS	USGS	08060203	32.01777778	-90.87694444	19138473	22266	92458
5	1	1	07292500	HOMOCHITTO RIVER AT ROSETTA, MS	USGS	08060205	31.32472222	-91.10944444	19112978	22301	65312
5	0	1	07297910	Pr Dog Twn Fk Red Rv nr Wayside, TX	USGS	11120103	34.83755505	-101.4140578	13925051	36976	65313
5	1	1	07299540	Prairie Dog Town Fork Red River near Childress, TX	USGS	11120105	34.56922722	-100.1940022	13956737	36996	65314
5	1	1	07300000	Salt Fork Red River near Wellington, TX	USGS	11120202	34.95755306	-100.2209492	13531193	37039	65315
5	1	0	07301500	North Fork Red River near Carter, OK	USGS	11120302	35.16810833	-99.50731278	13756121	38162	92617
5	1	0	07308200	Pease Rv nr Vernon, TX	USGS	11130105	34.17925232	-99.27813681	14003839	37167	92573
5	1	1	07308500	Red River near Burkburnett, TX	USGS	11130102	34.11009333	-98.53172333	13971012	37130	65318
5	1	1	07311000	East Cache Creek near Walters, OK	USGS	11130202	34.3623075	-98.28254694	674726	37209	92581
5	1	1	07311200	Blue Beaver Creek near Cache, OK	USGS	11130203	34.62340611	-98.56367111	572690	65319	65319
5	0	1	07311600	N Wichita Rv nr Paducah, TX	USGS	11130204	33.9506365	-100.0648269	13525140	37249	92564
5	0	1	07311630	MIDDLE WICHITA RIVER 240 M UPSTREAM OF FAF	USGS	11130204	33.795994	-100.074675	13525368	37258	92547
5	1	1	07311700	N Wichita Rv nr Truscott, TX	USGS	11130204	33.82064216	-99.78648219	13526822	37247	92549
5	0	1	07311782	S Wichita Rv at Low Flow Dam nr Guthrie, TX	USGS	11130205	33.62203029	-100.2089951	13520211	37263	92542
5	0	1	07311800	S Wichita Rv nr Benjamin, TX	USGS	11130205	33.64425703	-99.80092517	13520741	37259	92543
5	0	1	07311900	Wichita Rv nr Seymour, TX	USGS	11130206	33.70036966	-99.3886905	13733388	37259	92545
5	1	1	07312100	Wichita Rv nr Mabelle, TX	USGS	11130206	33.7600934	-99.14284945	13732942	38120	92546
5	1	1	07312500	Wichita Rv at Wichita Falls, TX	USGS	11130206	33.90954178	-98.53366629	13734150	37266	92554
5	1	0	07312700	Wichita Rv nr Charlie, TX	USGS	11130206	34.05315042	-98.29671387	13732288	37272	92569
5	1	1	07315500	Red River near Terral, OK	USGS	11130201	33.87871056	-97.93447972	3134443	37191	92550
5	1	1	07316000	Red River near Gainesville, TX	USGS	11130201	33.72788333	-97.16001306	3134651	37169	65320

Table S1. Description of sites, load data, and calculated loads used in regional nutrient SPARROW models.

Major river basin identifier	If tp_site	If tn_site	Station identifier	Station_name	WQ data program	8-digit cataloging unit	Station latitude	Station longitude	NHDPlus reach identifier	ERF1_2 reach identifier	Major river basin reach identifier	Station upstream area (km2)	DEM-estimated drainage area (km2)	RF1 estimate of mean flow (ft3/s)	Flow station identifier
5	1	1	07326500	Washita River at Anadarko, OK	USGS	11130302	35.08422833	-98.243385	683473	37349	92612	9468.996128	9445.9307	571.9	07326500
5	1	0	07328100	Washita River at Alex, OK	USGS	11130303	34.92590306	-97.77392722	19958172	37331	92605	12398.27256	12368.752	848.1	07328100
5	1	1	07331000	Washita River near Dickson, OK	USGS	11130304	34.23342722	-96.97584472	430522	37384	65321	18653.09358	18598.021	2256.1	07331000
5	1	1	07331600	Red River at Denison Dam nr Denison, TX	USGS	11140101	33.81899111	-96.56332639	8350531	37427	65322	102874.3234	102614.71	6402.1	07331600
5	1	1	07332500	Blue River near Blue, OK	USGS	11140102	33.99704083	-96.24109861	700694	37450	92563	1232.834288	1235.23	327.4	07332500
5	1	1	07335500	Red River at Arthur City, TX	USGS	11140101	33.875105	-95.50190222	8348933	37413	92548	115334.7556	115052.51	10336.1	07335500
5	1	1	07335700	Kiamichi River near Big Cedar, OK	USGS	11140105	34.63843694	-94.61272361	588170	65323	65323	103.8585188	102.46	83.4	07335700
5	1	1	07336200	Kiamichi River near Antlers, OK	USGS	11140105	34.24871194	-95.6052475	592734	37495	92574	2947.406344	2929.26	1496	07336200
5	1	1	07337000	Red Rv at Index, AR	USGS	11140106	33.55206716	-94.04130093	9544237	37515	65324	124397.1236	124019.56	14158.6	07337000
5	1	1	07337900	Glover River near Glover, OK	USGS	11140107	34.09760667	-94.90216667	616744	37544	92570	815.84622	829.84	425.8	07337900
5	1	0	07338720	MT FORK NEAR HATFIELD ARK	USGS	11140108	34.503333	-94.430556	629433	37562	92584	435.117984	439.58	299.1	07338750
5	0	1	07340300	COSSATOT R NR VANDERVOORT AR	USGS	11140109	34.37955418	-94.235763	3746094	65731	65731	232.0629248	141.48	178	07340300
5	1	0	07340520	COSSATOT RIV. WEST OF LOCKESBURG	USGS	11140109	33.971389	-94.222222	3748752	37578	92561	997.14538	1070.84	684.7	07340500
5	1	0	07341200	SALINE RIVER NR LOCKESBURG	USGS	11140109	33.961944	-94.052778	3748876	37573	92560	663.036928	662.67	304.5	07341200
5	1	0	07342500	S Sulphur Rv nr Cooper, TX	USGS	11140301	33.35649902	-95.59495624	4300941	37713	92529	1364.923676	1355.13	383.9	07342500
5	1	1	07343000	N Sulphur Rv nr Cooper, TX	USGS	11140301	33.47483009	-95.58773472	4300911	37705	92534	714.836688	791.64	264	07343000
5	1	1	07346000	Big Cypress Ck nr Jefferson, TX	USGS	11140306	32.74958345	-94.49881024	1017893	37956	37956	2201.4898	2290.8601	535	07346000
5	1	0	07346045	Black Cypress Bayou at Jefferson, TX	USGS	11140306	32.77791731	-94.3574153	1017865	37808	92499	945.34562	959.3099	313.7	07346045
5	1	1	07346070	Little Cypress Ck nr Jefferson, TX	USGS	11140307	32.71291998	-94.3460265	1042491	37815	92493	1748.2419	1805.0801	510.1	07346070
5	1	1	07355500	RED R @ ALEXANDRIA, LA	USGS	08040301	31.31296019	-92.4429148	19408130	37667	65328	174824.19	174267.3	36373.8	07355500
5	1	0	07356000	Ouachita River near Mount Ida, AR	USGS	08040101	34.61009806	-93.69741722	15234772	21947	92587	1072.255032	1081.3999	692.7	07356000
5	1	1	07362000	Ouachita River at Camden, AR	USGS	08040102	33.59650222	-92.81821806	22005200	21975	65329	13874.56572	13959.09	7691.8	07362000
5	1	1	07362065	OUACHITA RIVER BELOW CAMDEN, ARK.	USGS	08040201	33.48428444	-92.75321667	21956246	22016	92536	14506.92942	14614.899	8034.5	07362000
5	1	0	07362110	SMACKOVER CREEK N OF SMACKOVER ARK	USGS	08040201	33.379444	-92.719167	21954844	22018	92533	1064.485068	1070.5399	407	07362100
5	1	1	07362587	Alum Fork Saline River near Reform, AR	USGS	08040203	34.79731361	-92.9335075	21919761	22709	92594	69.929676	69.08	36.9	07362587
5	1	1	07364133	Bayou Bartholomew at Garrett Bridge, AR	USGS	08040205	33.86649222	-91.65623889	19469497	22059	92557	984.19544	1107.7	468.6	07364133
5	1	1	07364150	Bayou Bartholomew near McGehee, AR	USGS	08040205	33.62855278	-91.44640278	19470295	22065	92544	1491.833088	1660.33	705.6	07364150
5	1	1	07364200	BAYOU BARTHOLOMEW NEAR JONES, LOUISIANA	USGS	08040205	32.990405	-91.65567778	19474395	22058	92518	3074.315756	3439.8101	1265.3	07364200
5	1	1	07366200	Little Corney Bayou near Lillie, LA	USGS	08040206	32.92930641	-92.6329305	15221924	22070	92511	538.717504	438.31	220.4	07366200
5	1	1	07369500	TENSAS R @ TENDAL, LA	USGS	08050003	32.43208694	-91.36678139	19273960	22194	65330	800.306292	611.09	212.8	07369500
5	1	1	07373000	BIG CR AT POLLOCK LA	USGS	08040304	31.5362873	-92.40847139	19376616	22141	65331	132.089388	132.63	53.7	07373000
5	1	1	07373420	MISSISSIPPI R NR ST. FRANCISVILLE, LA	USGS	08070100	30.75851659	-91.39594643	19085457	22318	65708	2914513.496	2812681.56	528983.9	07295100
5	1	1	07375050	Tchefuncte River near Covington, LA	USGS	08090201	30.49463641	-90.16952437	18931936	65710	65710	375.54826	433.6	148	07375000
5	1	1	07375500	Tangipahoa River at Robert, LA	USGS	08070205	30.50658047	-90.36175242	18975529	22397	65332	1673.132248	1699.9	1211.6	07375500
5	1	1	07377000	Amite River near Darlington, LA	USGS	08070202	30.88906941	-90.84454541	18985842	22345	92417	1502.19304	1523.84	866	07377000
5	1	0	07380120	Amite River at Port Vincent, LA	USGS	08070202	30.33269391	-90.85204188	18990284	22342	65333	4133.620848	4029.51	2554.9	07380120
5	1	1	07381495	(COE) Atchafalaya River at Melville, LA	USGS	08080101	30.69074302	-91.73622618	15169599	65667	65667	241687.3202	366724.97	222556.1	07381490
5	1	1	07382000	BAYOU COCODRIE NR CLEARWATER, LA	USGS	08080102	31.00019083	-92.37957726	15147890	22451	92420	621.59712	580.2	215.6	07382000
5	1	1	07382500	Bayou Courtableau at Washington, LA	USGS	08080102	30.61825175	-92.05567412	15148368	22446	92404	1851.84142	1843.0499	661.3	07382500
5	1	1	08010000	BAYOU DES CANNES NR EUNICE, LA	USGS	08080201	30.48288889	-92.49069444	15140365	22476	92392	339.288428	379.45	190.7	08010000
5	1	1	08013500	Calcasieu River near Oberlin, LA	USGS	08080203	30.64047572	-92.81403683	15085713	22521	92403	1950.260964	1966.61	1087.1	08013500
5	1	1	08014500	Whiskey Chitto Creek Near Oberlin, LA	USGS	08080204	30.69875	-92.89319444	15078398	22539	92407	1320.89388	1264.3098	778.2	08014500
5	1	1	08015500	Calcasieu River near Kinder, LA	USGS	08080203	30.50255556	-92.91541667	15085965	65337	65337	4402.9796	4453.1597	2698.9	08015500
5	1	0	08018500	Sabine Rv nr Mineola, TX	USGS	12010002	32.61374667	-95.4857901	5275740	38897	92485	3514.613716	3561.1499	807.8	08018500
5	1	0	08020000	Sabine Rv nr Gladewater, TX	USGS	12010002	32.52708909	-94.96021751	5274290	38935	92477	7228.656508	7232.1499	1817.4	08020000
5	1	0	08022040	Sabine Rv nr Beckville, TX	USGS	12010002	32.32737887	-94.35353015	5278868	38927	92468	9295.466932	9335.6807	2639.2	08022040
5	1	1	08028500	Sabine River West of Merryville, Louisiana	USGS	12010005	30.746944	-93.608333	8330800	38988	92408	21313.01125	21470.562	7014.6	08028500
5	1	1	08030500	Sabine Rv nr Ruliff, TX	USGS	12010005	30.30381684	-93.74377844	8331804	38985	65339	24161.99805	24100.404	8502.6	08030500
5	1	1	08032000	Neches Rv nr Neches, TX	USGS	12020001	31.89239037	-95.43078599	4452936	39020	92451	2965.53626	2962.0303	708.5	08032000
5	1	0	08033500	Neches Rv nr Rockland, TX	USGS	12020003	31.025	-94.39944444	1109655	40944	92419	9417.196368	9405.8906	2557.2	08033500
5	1	1	08041000	Neches Rv at Evadale, TX	USGS	12020003	30.35576378	-94.09323728	1114217	39068	65340	20592.99459	20532.707	6966.6	08041000
5	1	1	08044500	W Fk Trinity Rv nr Boyd, TX	USGS	12030101	33.08539917	-97.55863596	1305941	41300	92519	4467.7293	4428.6304	276.3	08044500
5	1	1	08045850	Clear Fk Trinity Rv nr Weatherford, TX	USGS	12030102	32.74040699	-97.65197113	1268504	39175	92495	313.388548	311.57	34	08045850
5	1	1	08049500	W Fk Trinity Rv at Grand Prairie, TX	USGS	12030102	32.7987406	-97.02973015	1268384	39177	92500	7938.31322	7902.1299	862.2	08049500
5	1	1	08049700	WALNUT CREEK AT MATLOCK ROAD 2.6 MI NORTI	USGS	12030102	32.58086	-97.102135	1268648	39181	92482	162.6512464	164.24	20.5	08049700
5	1	1	08050410	ELM FORK TRINITY RIVER IMMEDIATELY DOWNST	USGS	12030103	33.582363	-97.128349	1274854	39203	92540	477.0763076	509.63	98.2	08050400
5	1	1	08051500	Clear Ck nr Sanger, TX	USGS	12030103	33.3362269	-97.1794587	1276892	39210	65582	764.04646	750.65	106.1	08051500
5	1	0	08052700	Little Elm Ck nr Aubrey, TX	USGS	12030103	33.28344977	-96.8927805	1278534	39186	92526	195.544094	191.91	48.8	08052700
5	1	1	08053000	Elm Fk Trinity Rv nr Lewisville, TX	USGS	12030103	33.04567733	-96.96111727	1278624	39185	41029	4333.049924	4315.96	764.2	08053000
5	1	1	08057200	White Rock Ck at Greenville Ave, Dallas, TX	USGS	12030105	32.88929207	-96.75666636	1260353	41356	92507	171.9752032	174.62	69	08057200
5	1	1	08057410	Trinity Rv bl Dallas, TX	USGS	12030105	32.70763139	-96.73583187	1260375	39249	65583	16259.94466	16204.199	2367.6	08057410
5	1	1	08058900	E Fk Trinity Rv at McKinney, TX	USGS	12030106	33.24400417	-96.60887971	1292088	41040	65584	424.758032	434.23	123.8	08058900
5	1	1	08062000	E Fk Trinity Rv nr Crandall, TX	USGS	12030106	32.63874423	-96.48526511	1293094	39261	92490	3253.024928	3255.3499	738.3	08062000
5	1	1	08062500	Trinity Rv nr Rosser, TX	USGS	12030105	32.42652988	-96.46304152	1262531	39244	92474	21100.63224	21040.188	3734.4	08062500
5	1	1	08062700	Trinity Rv at Trinidad, TX	USGS	12030105	32.13487534	-96.10580408	1262643	65341	92460	22113.31754	22048.438	4613.4	08062700
5	1	1	08064100	CHAMBERS CREEK AT FM 1126	USGS	12030109	32.197498	-96.521385	1446644	39312	65585	2090.120316	1654.6198	402.5	08064100
[illegible]	[illegible]	[illegible]	[illegible]	[illegible]	[illegible]	[illegible]	[illegible]	[illegible]	[illegible]	[illegible]	[illegible]	[illegible]	[illegible]	[illegible]	[illegible]

Daily flow record start date for prediction/Flow station upstream area (km2)	Daily flow record start date for prediction	Daily flow record end date for prediction	Mean daily flow (ft3/s)	[illegible]
[illegible]	[illegible]	[illegible]	[illegible]	[illegible]

Table S1. Description of sites, load data, and calculated loads used in regional nutrient SPARROW models.

Major river basin identifier	tp	If site	tn	If site	Station identifier	Station_name	WQ data program	8-digit cataloging unit	Station latitude	Station longitude	NHDPlus reach identifier	ERF1_2 reach identifier	Major river basin reach identifier	Station upstream area (km2)	DEM-estimated drainage area (km2)	RF1 estimate of mean flow (ft3/s)	Flow station identifier	Daily flow record start date for prediction'Flow station upstream area (km2)	Daily flow record start date for prediction	Daily flow record end date for prediction
5	1		0		HATCH122.1HR	HATCHIE R 122.1 HWY 100 BRIDGE NEAR TOONE		08010208	35.347222	-89.015278	14081134	21509	92635	3955.680902	3997.49	2727.5	07029500	3833.18224	10/1/1970	9/30/2007
5	1		1		OK121500-02-036	Dog Creek @ Spavinaw Flowline		11070105	36.24861	-95.59778	833378	36021	92677	283.5052665	273.46	74.6	07178520	193.9901012	10/1/1997	9/30/2004
5	1		1		OK121700-03-001	ILLINOIS RIVER AT ECHOTA ACCESS AREAS SITE 19		11110103	35.942667	-94.911722	400260	65545	92651	2446.818902	1639.6201	599.8	07196500	2483.798492	10/1/1970	9/30/2007
5	1		1		OK121700-03008B	ILLINOIS RIVER AT NO HEAD HOLLOW ACCESS POINT		11110103	35.96575	-94.909639	400078	65545	92655	2354.743534	1756.9102	632.3	07196500	2483.798492	10/1/1970	9/30/2007
5	1		1		OK121700-03008M	ILLINOIS RIVER AT ROUND HOLLOW ACCESS AREA		11110103	36.095833	-94.828	398522	65545	92659	2235.229056	2394.9202	958.5	07196500	2483.798492	10/1/1970	9/30/2007
5	0		1		OK121700-03028B	ILLINOIS RIVER ABOVE FLINT CREEK CONFLUENCE		11110103	36.173694	-94.720389	398108	37835	92804	1759.767088	2302.8901	926.7	07195500	1644.64238	10/1/1970	9/30/2007
5	1		1		OK121700-03028M	ILLINOIS RIVER AT CAMP PADDLETRAILS		11110103	36.128944	-94.6025	398298	37835	92666	1637.654074	2181.2102	884.6	07195500	1644.64238	10/1/1970	9/30/2007
5	1		0		RED0031	COSSATOT RIVER AT HWY 4 E OF WICKES ARK		11140109	34.3	-94.183333	3745712	37578	92577	371.1103156	365.68	278.6	07340300	232.0629248	10/1/1970	9/30/2007
5	1		1		SC098	NEOSHO RIVER NEAR LE ROY		11070204	38.08356	-95.65584	20889653	36117	92757	8242.012623	8268.0098	1631.4	07182510	7878.743496	10/1/1970	9/30/2007
5	1		1		SC213	Spring River Near Baxter Springs		11070207	37.02395	-94.72081	7591167	36157	92713	6301.984701	6339.29	2260.4	07188000	6500.86988	10/1/1970	9/30/2007
5	1		1		SC215	VERDIGRIS RIVER NEAR COFFEYVILLE		11070103	37.00553	-95.59228	21797449	35981	92712	8537.364494	8841.8301	2594.5	07170990	8650.55992	10/1/2002	9/30/2007
5	1		1		SC217	CANEY RIVER NEAR ELGIN		11070106	37.00375	-96.31618	21782934	36044	92710	1111.266726	1111.45	243.8	07172000	1152.54466	10/1/1970	9/30/2007
5	1		1		SC220	Medicine Lodge River Near Kiowa		11060003	37.03935	-98.46972	21014649	35838	92717	2310.010297	2310.1299	149.4	07149000	2338.759164	10/1/1970	9/30/2007
5	1		1		SC274	Cottonwood River Near Emporia		11070203	38.36582	-96.11486	20907153	36099	92766	4818.900593	4844.1299	825.6	07182250	4506.57912	10/1/1970	9/30/2007
5	1		1		SC280	NINNESCAH RIVER NEAR BELLE PLAINE		11030016	37.36385	-97.27648	21145422	35565	92739	5708.217003	5757.0996	572.3	07145500	5514.084452	10/1/1970	9/30/2007
5	1		1		SC282	Little Arkansas River At Valley Center		11030012	37.83215	-97.38802	21177332	35520	92748	3374.723284	3468.8501	374.4	07144200	3436.914076	10/1/1970	9/30/2007
5	1		1		SC522	COW CREEK NEAR WILLOWBROOK		11030011	38.11497	-98.00107	21187187	35512	92759	1888.259241	2216.49	67.2	07143300	1885.511264	10/1/1970	9/30/2007
5	1		1		SC523	Arkansas River Near Hutchinson		11030010	38.05712	-97.9932	22076721	35508	92756	88092.64025	101109.84	414.2	07141220	88064.77198	10/1/1998	9/30/2007
5	1		1		SC525	North Fork Ninnescah River Near Castleton		11030014	37.84446	-97.93593	21160597	38277	92749	2211.678554	2218.24	147.8	07144795	2333.579188	10/1/1970	9/30/2007
5	1		1		SC528	SLATE CREEK NEAR WELLINGTON		11030013	37.21628	-97.34836	21166319	35551	92729	482.1127983	507.65	61.7	07145700	398.858152	10/1/1970	9/30/2007
5	1		1		SC529	Chikaskia River Near Corbin		11060005	37.07957	-97.52865	20987069	35901	92719	2338.192475	2337.27	277.3	07151500	2056.450472	10/1/1975	9/30/2007
5	1		1		SC561	Verdigris River Near Altoona		11070101	37.52999	-95.67501	20955128	35946	92742	2714.341094	2726.28	689.4	07166500	2947.406344	10/1/1970	9/30/2007
[illegible]																				

Data S1. Text file describing the constituents considered when creating TN and TP, and protocols for combining those constituents.

/*
This text file was modified from a Statistical Analysis System (SAS) macro that was used to help create the datasets associated with the regional nutrient SPARROW models. It includes a list of nitrogen and phosphorus constituents that were considered for use in calculating total nitrogen and total phosphorus loads. The file also includes a description of the protocol (as defined by SAS code) for creating total nitrogen and total phosphorus values by combining constituents.
*/

```
*Parameter/constituent labels;
label v00600="Total nitrogen, unfiltered, milligrams per liter as N";
label v00605="Organic nitrogen, unfiltered, milligrams per liter as N";
label v00607="Organic nitrogen, filtered, milligrams per liter as N";
label v00608="Ammonia, filtered, milligrams per liter as N";
label v00610="Ammonia, unfiltered, milligrams per liter as N";
label v00613="Nitrite, filtered, milligrams per liter as N";
label v00615="Nitrite, unfiltered, milligrams per liter as N";
label v00618="Nitrate, filtered, milligrams per liter as N";
label v00620="Nitrate, unfiltered, milligrams per liter as N";
label v00623="Ammonia plus organic nitrogen, filtered, milligrams per liter as N";
label v00625="Ammonia plus organic nitrogen, unfiltered, milligrams per liter as N";
label v00630="Nitrite plus nitrate, unfiltered, milligrams per liter as N";
label v00631="Nitrite plus nitrate, filtered, milligrams per liter as N";
label v00635="Ammonia plus organic nitrogen, unfiltered, milligrams per liter as N";
label v00636="Ammonia plus organic nitrogen, filtered, milligrams per liter as N";
label v00665="Phosphorus, unfiltered, milligrams per liter as P";
label v49570="Particulate nitrogen, suspended in milligrams per liter as N";
label v49571="Dissolved nitrogen, filtered, in milligrams per liter as N";
label v62854="Total nitrogen, filtered, analytically determined, milligrams per liter as N";
label v62855="Total nitrogen, unfiltered, analytically determined, milligrams per liter as N";
label v71845="Ammonia, unfiltered, milligrams per liter as NH4";
label v71846="Ammonia, filtered, milligrams per liter as NH4";
label v71850="Nitrate, unfiltered, milligrams per liter as NO3";
label v71851="Nitrate, filtered, milligrams per liter as NO3";
label v71855="Nitrite, unfiltered, milligrams per liter as NO3";
label v71856="Nitrite, filtered, milligrams per liter as NO3";
label v71886="Phosphorus, unfiltered, milligrams per liter as PO4";
label v71887="Total nitrogen, unfiltered, milligrams per liter as NO3";
```

```
*Remark code labels;
label r00600="Total nitrogen, unfiltered, remark";
label r00605="Organic nitrogen, unfiltered, remark";
label r00607="Organic nitrogen, filtered, remark";
label r00608="Ammonia, filtered, remark";
label r00610="Ammonia, unfiltered, remark";
label r00613="Nitrite, filtered, remark";
label r00615="Nitrite, unfiltered, remark";
label r00618="Nitrate, filtered, remark";
label r00620="Nitrate, unfiltered, remark";
label r00623="Ammonia plus organic nitrogen, filtered, remark";
label r00625="Ammonia plus organic nitrogen, unfiltered, remark";
label r00630="Nitrite+nitrate, unfiltered, remark";
label r00631="Nitrite+nitrate, filtered, remark";
label r00635="Ammonia plus organic nitrogen, unfiltered, remark";
label r00636="Ammonia plus organic nitrogen, filtered, remark";
label r00665="Phosphorus, unfiltered, remark";
label r49570="Particulate nitrogen, suspended, remark";
label r49571="Dissolved nitrogen, filtered, remark";
label r62854="Total nitrogen, filtered, remark";
label r62855="Total nitrogen unfiltered, remark";
label r71845="Ammonia, unfiltered, remark";
label r71846="Ammonia, filtered, remark";
label r71850="Nitrate, unfiltered, remark";
label r71851="Nitrate, filtered, remark";
label r71855="Nitrite, unfiltered, remark";
label r71856="Nitrite, filtered, remark";
label r71886="Phosphorus, unfiltered, remark";
label r71887="Total nitrogen, unfiltered, remark";
```

```
*Updates to values based on remarks codes (also update remark codes where appropriate);
if r00600 in (">","L","N","Q","V","U","M") then v00600 =.;  if r00600 in (">","L","N","Q","V","U","M") then r00600 = ""; if r00600 = "K" then r00600 = "<";  if r00600 = "J" then r00600 = "E";
if r00605 in (">","L","N","Q","V","U","M") then v00605 =.;  if r00605 in (">","L","N","Q","V","U","M") then r00605 = ""; if r00605 = "K" then r00605 = "<";  if r00605 = "J" then r00605 = "E";
if r00607 in (">","L","N","Q","V","U","M") then v00607 =.;  if r00607 in (">","L","N","Q","V","U","M") then r00607 = ""; if r00607 = "K" then r00607 = "<";  if r00607 = "J" then r00607 = "E";
if r00608 in (">","L","N","Q","V","U","M") then v00608 =.;  if r00608 in (">","L","N","Q","V","U","M") then r00608 = ""; if r00608 = "K" then r00608 = "<";  if r00608 = "J" then r00608 = "E";
if r00610 in (">","L","N","Q","V","U","M") then v00610 =.;  if r00610 in (">","L","N","Q","V","U","M") then r00610 = ""; if r00610 = "K" then r00610 = "<";  if r00610 = "J" then r00610 = "E";
if r00613 in (">","L","N","Q","V","U","M") then v00613 =.;  if r00613 in (">","L","N","Q","V","U","M") then r00613 = ""; if r00613 = "K" then r00613 = "<";  if r00613 = "J" then r00613 = "E";
if r00615 in (">","L","N","Q","V","U","M") then v00615 =.;  if r00615 in (">","L","N","Q","V","U","M") then r00615 = ""; if r00615 = "K" then r00615 = "<";  if r00615 = "J" then r00615 = "E";
if r00618 in (">","L","N","Q","V","U","M") then v00618 =.;  if r00618 in (">","L","N","Q","V","U","M") then r00618 = ""; if r00618 = "K" then r00618 = "<";  if r00618 = "J" then r00618 = "E";
if r00620 in (">","L","N","Q","V","U","M") then v00620 =.;  if r00620 in (">","L","N","Q","V","U","M") then r00620 = ""; if r00620 = "K" then r00620 = "<";  if r00620 = "J" then r00620 = "E";
if r00623 in (">","L","N","Q","V","U","M") then v00623 =.;  if r00623 in (">","L","N","Q","V","U","M") then r00623 = ""; if r00623 = "K" then r00623 = "<";  if r00623 = "J" then r00623 = "E";
if r00625 in (">","L","N","Q","V","U","M") then v00625 =.;  if r00625 in (">","L","N","Q","V","U","M") then r00625 = ""; if r00625 = "K" then r00625 = "<";  if r00625 = "J" then r00625 = "E";
if r00630 in (">","L","N","Q","V","U","M") then v00630 =.;  if r00630 in (">","L","N","Q","V","U","M") then r00630 = ""; if r00630 = "K" then r00630 = "<";  if r00630 = "J" then r00630 = "E";
if r00631 in (">","L","N","Q","V","U","M") then v00631 =.;  if r00631 in (">","L","N","Q","V","U","M") then r00631 = ""; if r00631 = "K" then r00631 = "<";  if r00631 = "J" then r00631 = "E";
if r00635 in (">","L","N","Q","V","U","M") then v00635 =.;  if r00635 in (">","L","N","Q","V","U","M") then r00635 = ""; if r00635 = "K" then r00635 = "<";  if r00635 = "J" then r00635 = "E";
```

```
if r00636 in (">","L","N","Q","V","U","M") then v00636 =.;  if r00636 in (">","L","N","Q","V","U","M") then r00636 = ""; if r00636 = "K" then r00636 = "<";  if r00636 = "J" then r00636 = "E";
if r00665 in (">","L","N","Q","V","U","M") then v00665 =.;  if r00665 in (">","L","N","Q","V","U","M") then r00665 = ""; if r00665 = "K" then r00665 = "<";  if r00665 = "J" then r00665 = "E";
if r49570 in (">","L","N","Q","V","U","M") then v49570 =.;  if r49570 in (">","L","N","Q","V","U","M") then r49570 = ""; if r49570 = "K" then r49570 = "<";  if r49570 = "J" then r49570 = "E";
if r49571 in (">","L","N","Q","V","U","M") then v49571 =.;  if r49571 in (">","L","N","Q","V","U","M") then r49571 = ""; if r49571 = "K" then r49571 = "<";  if r49571 = "J" then r49571 = "E";
if r62854 in (">","L","N","Q","V","U","M") then v62854 =.;  if r62854 in (">","L","N","Q","V","U","M") then r62854 = ""; if r62854 = "K" then r62854 = "<";  if r62854 = "J" then r62854 = "E";
if r62855 in (">","L","N","Q","V","U","M") then v62855 =.;  if r62855 in (">","L","N","Q","V","U","M") then r62855 = ""; if r62855 = "K" then r62855 = "<";  if r62855 = "J" then r62855 = "E";
if r71845 in (">","L","N","Q","V","U","M") then v71845 =.;  if r71845 in (">","L","N","Q","V","U","M") then r71845 = ""; if r71845 = "K" then r71845 = "<";  if r71845 = "J" then r71845 = "E";
if r71846 in (">","L","N","Q","V","U","M") then v71846 =.;  if r71846 in (">","L","N","Q","V","U","M") then r71846 = ""; if r71846 = "K" then r71846 = "<";  if r71846 = "J" then r71846 = "E";
if r71850 in (">","L","N","Q","V","U","M") then v71850 =.;  if r71850 in (">","L","N","Q","V","U","M") then r71850 = ""; if r71850 = "K" then r71850 = "<";  if r71850 = "J" then r71850 = "E";
if r71851 in (">","L","N","Q","V","U","M") then v71851 =.;  if r71851 in (">","L","N","Q","V","U","M") then r71851 = ""; if r71851 = "K" then r71851 = "<";  if r71851 = "J" then r71851 = "E";
if r71855 in (">","L","N","Q","V","U","M") then v71855 =.;  if r71855 in (">","L","N","Q","V","U","M") then r71855 = ""; if r71855 = "K" then r71855 = "<";  if r71855 = "J" then r71855 = "E";
if r71856 in (">","L","N","Q","V","U","M") then v71856 =.;  if r71856 in (">","L","N","Q","V","U","M") then r71856 = ""; if r71856 = "K" then r71856 = "<";  if r71856 = "J" then r71856 = "E";
if r71886 in (">","L","N","Q","V","U","M") then v71886 =.;  if r71886 in (">","L","N","Q","V","U","M") then r71886 = ""; if r71886 = "K" then r71886 = "<";  if r71886 = "J" then r71886 = "E";
if r71887 in (">","L","N","Q","V","U","M") then v71887 =.;  if r71887 in (">","L","N","Q","V","U","M") then r71887 = ""; if r71887 = "K" then r71887 = "<";  if r71887 = "J" then r71887 = "E";

* Parameters 00600, 00605, 00618, 00620, 71845, 71846, 71851, 71856, and 71887;
* are calculated variables in NWIS.  However, some older values were manually;
* entered into NWIS.  These can be different than the sum of ammonia+organic
* and nitrate if there were updates to these parameters -- change calc_only;
* to 'Y' to ignore manually entered computed variables;
calc_only='N';
if calc_only='Y' then r00600="_";
if calc_only='Y' then v00600=.;
if calc_only='Y' then r00605="_";
if calc_only='Y' then v00605=.;
if calc_only='Y' then r00618="_";
if calc_only='Y' then v00618=.;
if calc_only='Y' then r00620="_";
if calc_only='Y' then v00620=.;
if calc_only='Y' then r71745="_";
if calc_only='Y' then v71745=.;
if calc_only='Y' then r71846="_";
if calc_only='Y' then v71846=.;
if calc_only='Y' then r71851="_";
if calc_only='Y' then v71851=.;
if calc_only='Y' then r71856="_";
if calc_only='Y' then v71856=.;
if calc_only='Y' then r71887="_";
if calc_only='Y' then v71887=.;

* Set computed variables to missing;
c00600=.;
c00605=.;
c00610=.;
c00615=.;
c00620=.;
c00625=.;
c00630=.;
c00665=.;
rc00600="_";
rc00605="_";
rc00610="_";
rc00615="_";
```

```
rc00620="_";
rc00625="_";
rc00630="_";
rc00665="_";

* Labels for computed values;
label c00600="Computed Total nitrogen, unfiltered, in mg/L as N"; *<--Total Nitrogen used in SPARROW models;
label c00605="Computed Organic nitrogen, unfiltered, in mg/L as N";
label c00610="Computed Ammonia, unfiltered, in mg/L as N";
label c00615="Computed NO2, unfiltered, in mg/L as N";
label c00620="Computed NO3, unfiltered, in mg/L as N";
label c00625="Computed NH3+orgN, unfiltered, in mg/L as N";
label c00630="Computed NO2+NO3, unfiltered, in mg/L as N";
label c00665="Computed Phosphorus, unfiltered, in mg/L as P"; *<--Total Phosphorus used in SPARROW models;

*----------------------------------------------------;
*--------Processing of nitrogen variables------------;
*----------------------------------------------------;

* Organic nitrogen variables;
* if Parameter 00605 is available use it, otherwise use 00607;
if c00605=. then mc00605=1;
rc00605=r00605;
c00605=v00605;
if c00605=. then mc00605=2;
if c00605=. and v00607 ne . then rc00605=r00607;
if c00605=. and v00607 ne . then c00605=v00607;
if c00605=. then mc00605=.;

* Ammonia variables;
* if Parameter 00610 is available use it, otherwise use Parameters 71845, 00608, or 71846 in;
* order of preference;
if c00610=. then mc00610=1;
rc00610=r00610;
c00610=v00610;
* need to transform Parameters 71845 and 71846 from as NH4 to as N;
if c00610=. then mc00610=2;
if c00610=. and v71845 ne . then rc00610=r71845;
if c00610=. and v71845 ne . then c00610=v71845*0.7778;
if c00610=. then mc00610=3;
if c00610=. and v00608 ne . then rc00610=r00608;
if c00610=. and v00608 ne . then c00610=v00608;
if c00610=. then mc00610=4;
if c00610=. and v71846 ne . then rc00610=r71846;
if c00610=. and v71846 ne . then c00610=v71846*0.7778;
if c00610=. then mc00610=.;

* Ammonia plus organic nitrogen variables ;
* if Parameter 00625 is available use it, if not use Parameter 00635, otherwise estimate it from Parameters;
* 00605 (organic nitrogen) and 00610 (ammonia) as determined in previous code, if still missing estimate using Parameters;
* 00623 or 00636 (filtered ammonia+organic);
if c00625=. then mc00625=1;
rc00625=r00625;
```

```
c00625=v00625;
if c00625=. then mc00625=2;
if c00625=. and v00635 ne . then rc00625=r00635;
if c00625=. and v00635 ne . then c00625=v00635;
* if both Parameters 00605 and 00610 are censored, sum the Parameters and set remark to "<";
if c00625=. then mc00625=3;
if c00625=. and c00610 ne . and rc00610="<" and c00605 ne . and rc00605="<" then rc00625="<";
if c00625=. and c00610 ne . and rc00610="<" and c00605 ne . and rc00605="<" then c00625=c00610 + c00605;
* if neither Parameters 00605 and 00610 are censored, sum the Parameters and set remark to "_";
if c00625=. then mc00625=4;
if c00625=. and c00610 ne . and rc00610 ne "<" and c00605 ne . and rc00605 ne "<" then rc00625="_";
if c00625=. and c00610 ne . and rc00610 ne "<" and c00605 ne . and rc00605 ne "<" then c00625=c00610 + c00605;
* if either Parameter 00605 and 00610 is censored but not both and the difference between the two is greater than a
* factor of 2, use a 1/2 substitution for the censored Parameter and sum the Parameters and set remark to "_";
if c00625=. then mc00625=5;
if c00625=. and c00610 ne . and rc00610="<" and c00605 ne . and rc00605 ne "<" and c00605>=2*c00610 then rc00625="_";
if c00625=. and c00610 ne . and rc00610="<" and c00605 ne . and rc00605 ne "<" and c00605>=2*c00610 then c00625=c00610/2 + c00605;
if c00625=. and c00610 ne . and rc00610 ne "<" and c00605 ne . and rc00605="<" and c00610>=2*c00605 then rc00625="_";
if c00625=. and c00610 ne . and rc00610 ne "<" and c00605 ne . and rc00605="<" and c00610>=2*c00605 then c00625=c00610 + c00605/2;
* if either Parameter 00605 and 00610 is censored but not both and the difference between the two is less than a
* factor of 2, sum the Parameters and set remark to "<";
if c00625=. then mc00625=6;
if c00625=. and c00610 ne . and rc00610="<" and c00605 ne . and rc00605 ne "<" and c00605<2*c00610 then rc00625="<";
if c00625=. and c00610 ne . and rc00610="<" and c00605 ne . and rc00605 ne "<" and c00605<2*c00610 then c00625=c00610 + c00605;
if c00625=. and c00610 ne . and rc00610 ne "<" and c00605 ne . and rc00605="<" and c00610<2*c00605 then rc00625="<";
if c00625=. and c00610 ne . and rc00610 ne "<" and c00605 ne . and rc00605="<" and c00610<2*c00605 then c00625=c00610 + c00605;

if c00625=. then mc00625=7;
if c00625=. and v00623 ne . then rc00625=r00623;
if c00625=. and v00623 ne . then c00625=v00623;
if c00625=. then mc00625=8;
if c00625=. and v00636 ne . then rc00625=r00636;
if c00625=. and v00636 ne . then c00625=v00636;
if c00625=. then mc00625=.;

* Nitrite variables;
* if Parameter 00615 is available use it, if not use Parameter 71855, otherwise in order of preference use;
* Parameter 00613 or 71856 (filtered nitrite);
if c00615=. then mc00615=1;
rc00615=r00615;
c00615=v00615;
* need to transform Parameter 71855 and 71856 from as NO3 to as N;
if c00615=. then mc00615=2;
if c00615=. and v71855 ne . then rc00615=r71855;
if c00615=. and v71855 ne . then c00615=v71855*0.2258;
if c00615=. then mc00615=3;
if c00615=. and v00613 ne . then rc00615=r00613;
if c00615=. and v00613 ne . then c00615=v00613;
if c00615=. then mc00615=4;
if c00615=. and v71856 ne . then rc00615=r71856;
if c00615=. and v71856 ne . then c00615=v71856*0.2258;
if c00615=. then mc00615=.;
```

```
* Nitrate variables;
* if Parameter 00620 is available use it, if not use Parameter 71850, otherwise in order of preference use;
* Parameter 00618 or 71851 (filtered nitrate);
if c00620=. then mc00620=1;
rc00620=r00620;
c00620=v00620;
* need to transform Parameter 71850 from as NO3 to as N;
if c00620=. then mc00620=2;
if c00620=. and v71850 ne . then rc00620=r71850;
if c00620=. and v71850 ne . then c00620=v71850*0.2258;
if c00620=. then mc00620=3;
if c00620=. and v00618 ne . then rc00620=r00618;
if c00620=. and v00618 ne . then c00620=v00618;
if c00620=. then mc00620=4;
if c00620=. and v71851 ne . then rc00620=r71851;
if c00620=. and v71851 ne . then c00620=v71851*0.2258;
if c00620=. then mc00620=.;

* Nitrate plus nitrate variables;
* if Parameter 00630 is available use it, if not estimate from Parameter 00631 (filtered nitrite+nitrate);
* if still missing estimate from 00615 (nitrite nitrogen) and 00620 (nitrate) as determined in previous code;
if c00630=. then mc00630=1;
rc00630=r00630;
c00630=v00630;
if c00630=. then mc00630=2;
if c00630=. and v00631 ne . then rc00630=r00631;
if c00630=. and v00631 ne . then c00630=v00631;
* if both Parameters 00615 and 00620 are censored, sum the Parameters and set remark to "<";
if c00630=. then mc00630=3;
if c00630=. and c00620 ne . and rc00620="<" and c00615 ne . and rc00615="<" then rc00630="<";
if c00630=. and c00620 ne . and rc00620="<" and c00615 ne . and rc00615="<" then c00630=c00620 + c00615;
* if neither Parameters 00615 and 00620 are censored, sum the Parameters and set remark to "_";
if c00630=. then mc00630=4;
if c00630=. and c00620 ne . and rc00620 ne "<" and c00615 ne . and rc00615 ne "<" then rc00630="_";
if c00630=. and c00620 ne . and rc00620 ne "<" and c00615 ne . and rc00615 ne "<" then c00630=c00620 + c00615;
* if either Parameter 00615 and 00620 is censored but not both and the difference between the two is greater than a
* factor of 2, use a 1/2 substitution for the censored Parameter and sum the Parameters and set remark to "_";
if c00630=. then mc00630=5;
if c00630=. and c00620 ne . and rc00620="<" and c00615 ne . and rc00615 ne "<" and c00615>=2*c00620 then rc00630="_";
if c00630=. and c00620 ne . and rc00620="<" and c00615 ne . and rc00615 ne "<" and c00615>=2*c00620 then c00630=c00620/2 + c00615;
if c00630=. and c00620 ne . and rc00620 ne "<" and c00615 ne . and rc00615="<" and c00620>=2*c00615 then rc00630="_";
if c00630=. and c00620 ne . and rc00620 ne "<" and c00615 ne . and rc00615="<" and c00620>=2*c00615 then c00630=c00620 + c00615/2;
* if either Parameter 00615 and 00620 is censored but not both and the difference between the two is less than a
* factor of 2, sum the Parameters and set remark to "<";
if c00630=. then mc00630=6;
if c00630=. and c00620 ne . and rc00620="<" and c00615 ne . and rc00615 ne "<" and c00615<2*c00620 then rc00630="<";
if c00630=. and c00620 ne . and rc00620="<" and c00615 ne . and rc00615 ne "<" and c00615<2*c00620 then c00630=c00620 + c00615;
if c00630=. and c00620 ne . and rc00620 ne "<" and c00615 ne . and rc00615="<" and c00620<2*c00615 then rc00630="<";
if c00630=. and c00620 ne . and rc00620 ne "<" and c00615 ne . and rc00615="<" and c00620<2*c00615 then c00630=c00620 + c00615;
if c00630=. then mc00630=.;

*Allow 00630 to be calculated if 00620 ne missing and 00615 eq missing;
if c00630=. then mc00630=7;
```

```
if c00630=. and c00620 ne . and rc00620="_" then rc00630="_";
if c00630=. and c00620 ne . and rc00620="_" then c00630=c00620;
if c00630=. and c00620 ne . and rc00620="<" then rc00630="<";
if c00630=. and c00620 ne . and rc00620="<" then c00630=c00620;

*-----Total Nitrogen-----;
* if Parameter 00600 is available use it, otherwise use in order of preference Parameters 62855 and 71887;
* then if available sum Parameters 49570 or 62854 and 49571 (dissolved and particulate nitrogen);
* finally estimate total nitrogen from Parameters 00625 (NH3+orgN) and 00630 (NO2+NO3) as determined in previous code;
if c00600=. then mc00600=1;
rc00600=r00600;
c00600=v00600;
if c00600=. then mc00600=2;
if c00600=. and v62855 ne . then rc00600=r62855;
if c00600=. and v62855 ne . then c00600=v62855;
* need to transform Parameter 71887 from as NO3 to as N;
if c00600=. then mc00600=3;
if c00600=. and v71887 ne . then rc00600=r71887;
if c00600=. and v71887 ne . then c00600=v71887*0.2258;
* if both Parameters 49570 and 49571 are censored, sum the Parameters and set remark to "<";
if c00600=. then mc00600=4;
if c00600=. and v49570 ne . and r49570="<" and v49571 ne . and r49571="<" then rc00600="<";
if c00600=. and v49570 ne . and r49570="<" and v49571 ne . and r49571="<" then c00600=v49570 + v49571;
* if neither Parameters 49570 and 49571 are censored, sum the Parameters and set remark to "_";
if c00600=. then mc00600=5;
if c00600=. and v49570 ne . and r49570 ne "<" and v49571 ne . and r49571 ne "<" then rc00600="_";
if c00600=. and v49570 ne . and r49570 ne "<" and v49571 ne . and r49571 ne "<" then c00600=v49570 + v49571;
* if either Parameter 49571 and 49570 is censored but not both and the difference between the two is greater than a
* factor of 2, use a 1/2 substitution for the censored Parameter and sum the Parameters and set remark to "_";
if c00600=. then mc00600=6;
if c00600=. and v49570 ne . and r49570="<" and v49571 ne . and r49571 ne "<" and v49571>=2*v49570 then rc00600="_";
if c00600=. and v49570 ne . and r49570="<" and v49571 ne . and r49571 ne "<" and v49571>=2*v49570 then c00600=v49570/2 + v49571;
if c00600=. and v49570 ne . and r49570 ne "<" and v49571 ne . and r49571="<" and v49570>=2*v49571 then rc00600="_";
if c00600=. and v49570 ne . and r49570 ne "<" and v49571 ne . and r49571="<" and v49570>=2*v49571 then c00600=v49570 + v49571/2;
* if either Parameter 49571 and 49570 is censored but not both and the difference between the two is less than a
* factor of 2, sum the Parameters and set remark to "<";
if c00600=. then mc00600=7;
if c00600=. and v49570 ne . and r49570="<" and v49571 ne . and r49571 ne "<" and v49571<2*v49570 then rc00600="<";
if c00600=. and v49570 ne . and r49570="<" and v49571 ne . and r49571 ne "<" and v49571<2*v49570 then c00600=v49570 + v49571;
if c00600=. and v49570 ne . and r49570 ne "<" and v49571 ne . and r49571="<" and v49570<2*v49571 then rc00600="<";
if c00600=. and v49570 ne . and r49570 ne "<" and v49571 ne . and r49571="<" and v49570<2*v49571 then c00600=v49570 + v49571;
* if both Parameters 49570 and 62854 are censored, sum the Parameters and set remark to "<";
if c00600=. then mc00600=8;
if c00600=. and v49570 ne . and r49570="<" and v62854 ne . and r62854="<" then rc00600="<";
if c00600=. and v49570 ne . and r49570="<" and v62854 ne . and r62854="<" then c00600=v49570 + v62854;
* if neither Parameters 49570 and 62854 are censored, sum the Parameters and set remark to "_";
if c00600=. then mc00600=9;
if c00600=. and v49570 ne . and r49570 ne "<" and v62854 ne . and r62854 ne "<" then rc00600="_";
if c00600=. and v49570 ne . and r49570 ne "<" and v62854 ne . and r62854 ne "<" then c00600=v49570 + v62854;
* if either Parameter 62854 and 49570 is censored but not both and the difference between the two is greater than a
* factor of 2, use a 1/2 substitution for the censored Parameter and sum the Parameters and set remark to "_";
if c00600=. then mc00600=10;
if c00600=. and v49570 ne . and r49570="<" and v62854 ne . and r62854 ne "<" and v62854>=2*v49570 then rc00600="_";
```

```
if c00600=. and v49570 ne . and r49570="<" and v62854 ne . and r62854 ne "<" and v62854>=2*v49570 then c00600=v49570/2 + v62854;
if c00600=. and v49570 ne . and r49570 ne "<" and v62854 ne . and r62854="<" and v49570>=2*v62854 then rc00600="_";
if c00600=. and v49570 ne . and r49570 ne "<" and v62854 ne . and r62854="<" and v49570>=2*v62854 then c00600=v49570 + v62854/2;
* if either Parameter 62854 and 49570 is censored but not both and the difference between the two is less than a
* factor of 2, sum the Parameters and set remark to "<";
if c00600=. then mc00600=11;
if c00600=. and v49570 ne . and r49570="<" and v62854 ne . and r62854 ne "<" and v62854<2*v49570 then rc00600="<";
if c00600=. and v49570 ne . and r49570="<" and v62854 ne . and r62854 ne "<" and v62854<2*v49570 then c00600=v49570 + v62854;
if c00600=. and v49570 ne . and r49570 ne "<" and v62854 ne . and r62854="<" and v49570<2*v62854 then rc00600="<";
if c00600=. and v49570 ne . and r49570 ne "<" and v62854 ne . and r62854="<" and v49570<2*v62854 then c00600=v49570 + v62854;
* if both Parameters 00625 and 00630 are censored, sum the Parameters and set remark to "<";
if c00600=. then mc00600=12;
if c00600=. and c00625 ne . and rc00625="<" and c00630 ne . and rc00630="<" then rc00600="<";
if c00600=. and c00625 ne . and rc00625="<" and c00630 ne . and rc00630="<" then c00600=c00625 + c00630;
* if neither Parameters 00625 and 00630 are censored, sum the Parameters and set remark to "_";
if c00600=. then mc00600=13;
if c00600=. and c00625 ne . and rc00625 ne "<" and c00630 ne . and rc00630 ne "<" then rc00600="_";
if c00600=. and c00625 ne . and rc00625 ne "<" and c00630 ne . and rc00630 ne "<" then c00600=c00625 + c00630;
* if either Parameter 00630 and 00625 is censored but not both and the difference between the two is greater than a
* factor of 2, use a 1/2 substitution for the censored Parameter and sum the Parameters and set remark to "_";
if c00600=. then mc00600=14;
if c00600=. and c00625 ne . and rc00625="<" and c00630 ne . and rc00630 ne "<" and c00630>=2*c00625 then rc00600="_";
if c00600=. and c00625 ne . and rc00625="<" and c00630 ne . and rc00630 ne "<" and c00630>=2*c00625 then c00600=c00625/2 + c00630;
if c00600=. and c00625 ne . and rc00625 ne "<" and c00630 ne . and rc00630="<" and c00625>=2*c00630 then rc00600="_";
if c00600=. and c00625 ne . and rc00625 ne "<" and c00630 ne . and rc00630="<" and c00625>=2*c00630 then c00600=c00625 + c00630/2;
* if either Parameter 00630 and 00625 is censored but not both and the difference between the two is less than a
* factor of 2, sum the Parameters and set remark to "<";
if c00600=. then mc00600=15;
if c00600=. and c00625 ne . and rc00625="<" and c00630 ne . and rc00630 ne "<" and c00630<2*c00625 then rc00600="<";
if c00600=. and c00625 ne . and rc00625="<" and c00630 ne . and rc00630 ne "<" and c00630<2*c00625 then c00600=c00625 + c00630;
if c00600=. and c00625 ne . and rc00625 ne "<" and c00630 ne . and rc00630="<" and c00625<2*c00630 then rc00600="<";
if c00600=. and c00625 ne . and rc00625 ne "<" and c00630 ne . and rc00630="<" and c00625<2*c00630 then c00600=c00625 + c00630;
if c00600=. then mc00600=.;

*----------------------------------------------------;
*--------Processing of phosphorus variables----------;
*----------------------------------------------------;

*-----Total Phosphorus-----;
* if Parameter 00665 is available use it, otherwise use Parameter 71866;
if c00665=. then mc00665=1;
rc00665=r00665;
c00665=v00665;
* need to transform Parameter 71886 from as PO4 to as P;
if c00665=. then mc00665=2;
if c00665=. and v71886 ne . then rc00665=r71886;
if c00665=. and v71886 ne . then c00665=v71886*0.3261;
if c00665=. then mc00665=.;
```

Table S2. Sampling agencies associated with nutrient water-quality data used in regional SPARROW models, by Major River Basin (MRB).

MRB	Agency Name
1	Delaware Dept of Natural Resources and Environmental Control
	Delaware River Basin Commission
	District of Columbia, Department of Health
	Maryland Department of Natural Resources
	Maryland Department of the Environment
	Montgomery County Dept of Environmental Protection, Maryland
	National Park Service
	New Hampshire Department of Environmental Services
	New Jersey Department of Environmental Protection
	New York State Department of Environmental Conservation
	Pennsylvania Department of Environmental Protection
	Susquehanna River Basin Commission
	Tennessee Valley Authority
	US Army Corps of Engineers
	US Forest Service
	USEPA
	USGS
	Vermont Department of Environmental Conservation
	Virginia Department of Environmental Quality
	West Virginia Deptartment of Natural Resources
2	Alabama Department of Environmental Management
	Environmental Protection Commission of Hillsborough County, FL
	Florida Department of Environmental Protection
	Georgia Department of Natural Resources
	Mississippi Department of Natural Resources
	North Carolina Dept of Environment and Natural Resources
	South Carolina Department of Health and Environmental Control
	St. Johns River Water Management District, Florida
	Suwannee River Water Management District, Florida
	Tennessee Department of Environment and Conservation
	Tennessee Valley Authority
	USGS
	Virginia Department of Environmental Quality
3	Heidelberg College
	Illinois Environmental Protection Agency
	Indiana Department of Environmental Management
	Iowa Department of Natural Resources
	Kentucky Dept of Natural Resources and Environmental Protection
	Maryland Department of Natural Resources
	Maryland Department of the Environment
	Michigan Department of Environmental Quality
	Minnesota Pollution Control Agency
	New York State Department of Environmental Conservation
	North Carolina Dept of Environment and Natural Resources
	North Dakota Department of Health, Division of Water Quality
	Ohio Environmental Protection Agency
	Ohio River Valley Water Sanitation Commission
	Pennsylvania Department of Environmental Protection
	South Dakota Dept of Environment and Natural Resources
	Tennessee Department of Environment and Conservation
	US Army Corps of Engineers
	US Forest Service
	USGS
	Virginia Department of Environmental Quality
	West Virginia Department of Environmental Protection
	Western Lake Superior Sanitary District
	Wisconsin Department of Natural Resources
4	Centennial Water and Sanitation District, Colorado
	Colorado Department of Public Health and Environment
	Denver Regional Council of Governments
	Iowa Department of Natural Resources
	Kansas Department of Health and Environment
	Metro Waste Water Reclamation District, Colorado
	Minnesota Pollution Control Agency
	Missouri Department of Natural Resources
	Montana Department of Environmental Quality
	National Park Service
	Nebraska Department of Environmental Quality
	North Dakota Department of Health
	South Dakota Dept of Environment and Natural Resources
	US Army Corps of Engineers
	USEPA
	USGS
5	Arkansas Department of Environmental Quality
	Arkansas Department of Pollution Control and Ecology
	Colorado Department of Public Health and Environment
	Kansas Department of Health and Environment
	Kentucky Dept of Natural Resources and Environmental Protection
	Louisiana Department of Environmental Quality
	Mississippi Department of Environmental Quality
	Mississippi Department of Natural Resources
	National Park Service
	Oklahoma Conservation Commission
	Oklahoma Department of Agriculture
	Oklahoma Department of Pollution Control
	Oklahoma Water Resources Board
	Tennessee Department of Environment and Conservation
	Texas Commission on Environmental Quality
	Texas Water Commission
	US Army Corps of Engineers
	US Forest Service
	USGS
7	Clean Water Services, Washington County, Oregon
	King County, Washington
	Nevada Department of Conservation and Natural Resources
	Oregon Department of Environmental Quality
	Skagit County, Washington
	Snohomis County, Washington
	US Bureau of Reclamation
	USEPA
	USGS
	Washington State Department of Ecology

Table S3. Summary statistics, by Major River Basin, for selected environmental characteristics of monitored watersheds versus all watersheds in the regional SPARROW model areas.

Statistic	MRB Sites							NHDPlus Reaches						
	All regions	MRB1	MRB2	MRB3	MRB4	MRB5	MRB7	All regions	MRB1	MRB2	MRB3	MRB4	MRB5	MRB7
Number of sites or reaches	2,739	491	380	848	321	468	229	2,144,926	187,171	391,998	483,320	450,460	400,046	231,931
Mean														
Drainage area (km^2)	13103.5	2,599.8	4,529.6	9,624.9	35,037.1	18,255.3	21,459.9	2,030.9	414.7	491.6	1,704.3	3,453.1	3,239.8	1,770.4
Mean annual streamflow (m^3/s)	89.4	43.9	71.2	86.0	62.3	87.4	271.5	12.4	6.9	8.3	13.6	7.7	18.2	20.4
Mean annual precipitation (cm)	100.8	111.0	131.8	93.2	61.3	97.1	118.8	97.9	110.3	134.1	94.7	58.1	106.2	96.2
Mean air temperature (^{o}C)	11.1	9.9	16.0	9.0	8.0	16.1	6.9	11.2	9.2	16.5	9.0	8.0	15.9	6.5
Percent water	1.3	1.4	1.1	1.7	0.8	1.1	1.1	1.4	2.6	1.9	1.6	0.7	1.5	0.6
Percent urban	5.7	11.8	6.3	4.7	2.5	4.7	2.7	2.2	6.2	3.4	2.4	0.6	1.5	0.5
Percent barren	1.2	0.9	2.4	0.4	0.7	0.8	4.4	1.5	1.0	2.8	0.4	0.9	0.7	4.2
Percent forest	40.6	60.1	59.9	28.5	17.1	34.8	55.7	39.5	66.8	58.3	39.0	11.6	33.9	50.6
Percent range	11.2	0.0	2.0	1.1	38.0	25.1	22.3	19.0	0.1	0.8	1.1	50.2	23.0	34.5
Percent pasture	15.1	16.9	10.4	17.4	13.9	18.3	5.9	13.6	12.1	10.9	19.1	13.2	15.8	4.6
Percent cropland	21.1	5.5	11.2	41.8	25.3	13.3	4.2	18.0	5.6	12.5	31.1	21.5	17.6	4.1
Percent wetland	3.5	3.4	6.8	4.4	1.4	2.0	0.4	4.8	5.3	9.4	5.2	1.1	6.0	0.6
Standard deviation														
Drainage area (km^2)	78219.7	7,094.3	9,161.5	30,087.5	135,238.6	134,588.0	76,360.2	40,220.4	2,933.3	4,118.1	31,917.7	44,719.0	70,055.9	20,971.7
Mean annual streamflow (m^3/s)	514.3	118.1	171.7	304.8	253.0	930.5	920.2	250.8	47.9	79.0	184.9	87.5	495.0	245.7
Mean annual precipitation (cm)	34.1	10.3	16.1	17.8	20.7	31.1	70.2	40.1	10.3	16.0	19.4	24.3	33.9	70.1
Mean air temperature (^{o}C)	4.4	2.4	2.7	2.5	3.1	3.4	2.4	4.8	3.2	2.3	2.9	3.0	2.8	2.8
Percent water	1.9	1.7	1.4	2.7	1.0	1.3	1.6	5.7	6.7	7.5	5.4	3.9	5.8	3.8
Percent urban	12.9	19.8	10.4	9.7	11.0	11.5	8.8	8.4	14.8	10.1	8.3	4.3	6.7	3.6
Percent barren	2.5	1.5	3.0	0.9	2.0	1.8	4.7	5.4	3.3	6.1	2.4	5.1	3.8	9.7
Percent forest	30.4	22.6	21.9	28.1	24.7	27.9	27.8	35.4	26.5	28.6	33.2	22.1	35.1	37.4
Percent range	21.4	0.1	7.2	2.6	27.8	26.6	22.4	32.0	1.1	4.9	3.6	36.6	32.7	35.9
Percent pasture	13.9	16.0	6.8	11.7	14.5	17.6	8.4	17.3	17.0	13.4	17.6	17.7	19.5	12.6
Percent cropland	25.6	7.5	10.8	30.0	24.0	17.3	7.6	25.7	9.5	16.0	31.8	26.7	26.3	13.3
Percent wetland	6.2	4.1	9.1	7.6	1.6	3.7	0.6	13.9	12.0	19.6	12.0	4.6	18.4	4.2
Minimum														
Drainage area (km^2)	0.8	0.8	15.3	1.8	1.8	33.7	2.4	0.0	0.0	0.0	0.0	0.0	0.0	0.0
Mean annual streamflow (m^3/s)	0.0	0.0	0.2	0.0	0.0	0.1	0.0	0.0	0.0	0.0	0.0	0.0	0.0	0.0
Mean annual precipitation (cm)	25.5	83.2	101.5	42.0	29.4	39.1	25.5	8.6	8.6	93.5	14.7	14.5	25.0	16.8
Mean air temperature (^{o}C)	-1.3	3.5	9.5	2.4	0.0	-1.3	0.1	-4.0	0.3	8.0	1.4	-4.0	-2.9	-3.1
Percent water	0.0	0.0	0.0	0.0	0.0	0.0	0.0	0.0	0.0	0.0	0.0	0.0	0.0	0.0
Percent urban	0.0	0.0	0.0	0.0	0.0	0.0	0.0	0.0	0.0	0.0	0.0	0.0	0.0	0.0
Percent barren	0.0	0.0	0.0	0.0	0.0	0.0	0.0	0.0	0.0	0.0	0.0	0.0	0.0	0.0
Percent forest	0.0	0.9	3.9	0.1	0.0	0.0	0.0	0.0	0.0	0.0	0.0	0.0	0.0	0.0
Percent range	0.0	0.0	0.0	0.0	0.0	0.0	0.1	0.0	0.0	0.0	0.0	0.0	0.0	0.0
Percent pasture	0.0	0.0	0.0	0.1	0.0	0.0	0.0	0.0	0.0	0.0	0.0	0.0	0.0	0.0
Percent cropland	0.0	0.0	0.0	0.0	0.0	0.0	0.0	0.0	0.0	0.0	0.0	0.0	0.0	0.0
Percent wetland	0.0	0.0	0.0	0.0	0.0	0.0	0.0	0.0	0.0	0.0	0.0	0.0	0.0	0.0
25th percentile														
Drainage area (km^2)	466.9	132.1	364.3	708.6	995.1	651.6	744.4	2.1	2.1	1.6	2.3	2.3	2.2	2.5
Mean annual streamflow (m^3/s)	3.3	2.1	5.3	5.8	0.6	3.5	12.9	0.0	0.0	0.0	0.0	0.0	0.0	0.0
Mean annual precipitation (cm)	80.8	104.7	119.6	81.7	44.2	72.9	68.0	68.9	104.1	120.7	81.8	39.9	77.6	45.3
Mean air temperature (^{o}C)	7.8	8.1	14.5	7.5	5.7	14.1	5.4	7.2	6.9	14.9	6.9	6.2	14.2	4.5
Percent water	0.3	0.4	0.3	0.3	0.2	0.3	0.2	0.0	0.1	0.0	0.0	0.0	0.0	0.0
Percent urban	0.6	1.3	1.3	1.0	0.2	0.5	0.1	0.0	0.0	0.0	0.0	0.0	0.0	0.0
Percent barren	0.1	0.1	0.6	0.0	0.0	0.1	1.4	0.0	0.0	0.0	0.0	0.0	0.0	0.0
Percent forest	10.7	41.4	47.6	6.9	1.0	10.7	32.2	2.5	49.8	37.1	7.5	0.0	1.0	4.7
Percent range	0.0	0.0	0.0	0.0	10.7	0.1	4.9	0.0	0.0	0.0	0.0	10.8	0.0	4.2
Percent pasture	4.4	2.8	5.6	9.4	3.4	3.8	0.5	0.3	0.2	1.2	4.2	0.0	1.2	0.0
Percent cropland	1.9	1.2	3.2	11.3	1.5	1.4	0.1	0.1	0.2	1.2	2.3	0.0	0.2	0.0
Percent wetland	0.2	0.4	0.4	0.6	0.3	0.0	0.1	0.0	0.1	0.0	0.0	0.0	0.0	0.0
Median														
Drainage area (km^2)	1516.0	449.5	1,182.9	1,837.7	3,204.3	1,764.1	2,317.2	6.3	6.1	4.4	6.8	6.7	7.3	7.1
Mean annual streamflow (m^3/s)	11.7	7.1	16.7	15.7	2.3	9.3	48.5	0.0	0.1	0.1	0.1	0.0	0.0	0.1
Mean annual precipitation (cm)	101.9	111.2	128.4	93.6	55.4	96.7	99.4	100.8	109.8	133.2	93.6	48.6	113.0	74.8
Mean air temperature (^{o}C)	10.6	10.3	15.6	9.5	7.2	16.9	6.8	11.0	9.1	16.6	9.2	7.8	16.0	6.5
Percent water	0.7	0.7	0.7	0.8	0.5	0.7	0.6	0.1	0.5	0.2	0.1	0.1	0.3	0.0
Percent urban	1.6	3.4	2.8	1.9	0.4	1.1	0.4	0.0	0.6	0.1	0.3	0.0	0.0	0.0
Percent barren	0.3	0.4	1.3	0.1	0.1	0.4	3.1	0.0	0.1	0.4	0.0	0.0	0.0	0.2
Percent forest	37.5	65.3	66.2	15.9	5.7	28.5	63.2	33.2	74.0	62.6	30.7	0.9	19.2	59.9
Percent range	0.1	0.0	0.0	0.1	39.1	16.8	11.5	0.1	0.0	0.0	0.0	51.9	0.3	18.2
Percent pasture	11.2	12.4	9.3	15.6	8.2	12.4	3.6	6.2	3.6	5.8	14.6	5.2	7.6	0.0
Percent cropland	7.9	3.3	7.4	43.6	21.8	5.8	0.6	4.5	2.0	5.8	18.7	8.0	3.2	0.0
Percent wetland	1.0	1.6	2.0	1.6	0.9	0.4	0.2	0.2	1.8	1.0	0.5	0.1	0.1	0.0
75th percentile														
Drainage area (km^2)	5815.0	1,555.1	4,687.6	6,461.7	13,605.7	6,318.6	9,227.4	34.2	29.1	23.7	37.4	37.6	38.8	38.7
Mean annual streamflow (m^3/s)	43.4	21.9	64.5	53.4	12.5	24.3	137.2	0.2	0.5	0.3	0.3	0.0	0.2	0.4
Mean annual precipitation (cm)	118.0	117.1	143.2	103.6	74.2	118.4	157.4	124.0	116.3	144.1	106.7	73.4	133.7	123.8
Mean air temperature (^{o}C)	13.8	11.6	17.3	10.6	11.0	18.4	8.7	15.2	11.8	18.0	11.3	10.2	17.6	8.6
Percent water	1.4	1.8	1.3	1.9	0.8	1.3	1.4	0.7	2.2	0.9	0.7	0.4	0.9	0.1
Percent urban	4.1	11.4	6.8	3.7	1.3	2.9	1.2	0.8	3.9	1.7	1.4	0.2	0.5	0.1
Percent barren	1.2	0.9	3.1	0.3	0.6	0.9	5.8	0.5	0.6	3.1	0.0	0.1	0.2	3.9
Percent forest	69.2	78.0	74.6	45.6	20.5	57.1	80.4	73.5	88.0	81.9	68.3	10.2	67.2	86.0
Percent range	8.2	0.0	0.0	0.8	60.5	47.0	36.8	25.3	0.0	0.0	0.5	87.0	44.3	63.1
Percent pasture	21.1	24.6	13.9	22.1	20.6	27.8	6.6	20.8	18.4	15.7	30.0	20.1	23.3	1.0
Percent cropland	35.8	6.4	15.8	69.7	42.6	18.3	4.8	27.3	6.7	17.9	57.2	37.7	25.3	0.0
Percent wetland	3.7	5.1	11.0	4.4	1.7	2.0	0.5	2.4	5.3	8.8	3.9	0.6	1.8	0.1
Maximum														
Drainage area (km^2)	2801775.5	70,085.8	103,558.9	433,743.1	1,227,819.2	2,801,775.5	621,395.3	2,804,304.2	71,153.0	112,147.7	1,710,119.5	1,231,824.9	2,804,304.2	624,155.2
Mean annual streamflow (m^3/s)	19915.7	1,117.8	2,142.3	3,704.1	2,114.8	19,915.7	8,224.9	19,960.4	1,132.9	2,163.6	8,150.6	2,127.0	19,960.4	8,372.4
Mean annual precipitation (cm)	404.0	156.3	226.8	145.2	117.3	169.8	404.0	660.2	235.8	280.1	181.7	269.7	181.9	660.2
Mean air temperature (^{o}C)	22.6	14.5	22.6	14.4	13.4	21.3	11.7	24.9	15.4	24.9	15.0	13.8	23.2	13.0
Percent water	27.0	14.1	12.1	27.0	7.0	7.5	14.9	100.0	100.0	100.0	100.0	100.0	100.0	100.0
Percent urban	98.8	98.8	73.0	78.4	95.1	86.0	61.1	100.0	100.0	100.0	100.0	100.0	100.0	100.0
Percent barren	34.8	11.3	27.6	8.4	24.2	29.8	34.8	100.0	100.0	100.0	100.0	100.0	100.0	100.0
Percent forest	100.0	100.0	99.6	98.3	91.5	99.5	98.4	100.0	100.0	100.0	100.0	100.0	100.0	100.0
Percent range	99.4	1.4	41.6	32.9	94.2	96.6	99.4	100.0	82.6	100.0	100.0	100.0	100.0	100.0
Percent pasture	77.5	68.7	36.4	64.1	77.5	75.1	43.7	100.0	100.0	100.0	100.0	100.0	100.0	100.0
Percent cropland	94.3	52.7	66.9	94.3	84.6	80.2	49.4	100.0	100.0	100.0	100.0	100.0	100.0	100.0
Percent wetland	57.1	21.2	45.7	57.1	10.4	27.2	3.6	100.0	100.0	100.0	100.0	100.0	100.0	100.0

Data S2: Detailed Description of the Load Estimates from Fluxmaster and Factors Affecting the Accuracy in their Estimations

Load Estimation

Computation of detrended long-term mean annual loads for each final load site used in the MRB SPARROW models is based on the regression methods developed by Cohn (2005) and implemented in the program Fluxmaster (Schwarz *et al.* 2006). Detrended mean annual loads provide an estimate of conditions normalized to a base year. The use of detrended mean annual loads in SPARROW models helps compensate for differences in the length and amount of monitoring data among sites, and minimizes the inherent noise introduced by year-to-year variations in rainfall facilitating the identification of environmental factors that affect loading over long periods (Preston *et al.*, 2009). The detrended load estimates are based on two models: a water-quality model and a flow model used to remove trends in streamflow. The water-quality model (Equation 1) relates the logarithm of concentration at time *t*, c_t, to the logarithm of flow, q_t, a decimal time term to represent trend, T_t, sine and cosine functions of decimal time to account for seasonal variation, and a model residual, e_t,

$$c_t = b_0 + b_q q_t + b_T T_t + b_s \sin(2\pi T_t) + b_c \cos(2\pi T_t) + e_t, \qquad (1)$$

where b_0, b_q, b_T, b_s, and b_c are fixed coefficients estimated for each site by the ordinary least squares method or, if some of the c_t measurements are censored, by the adjusted maximum likelihood method (Cohn, 2005), and e_t is assumed to be independent and normally distributed with mean 0 and variance σ_e^2.

Detrended flow is estimated using a flow model with the form

$$q_t = a_0 + a_T T_t + a_s \sin(2\pi T_t) + a_c \cos(2\pi T_t) + u_t\ , \qquad (2)$$

where a_0, a_T, a_s, and a_c are model parameters estimated using the maximum likelihood SAS Autoreg procedure and (SAS Institute, Inc., 2004; Note: Any use of trade, product, or firm names is for description purposes only and does not imply endorsement by the U.S. Government), and u_t is a model residual that is assumed to be correlated across time according to a 30-day lag autoregressive model. In some models, a second-order harmonic of the sine and cosine functions was included and a 10-day lag autoregressive model is used. Final detrended flow, $\tilde{q}_t$, is then estimated using the relation

$$\tilde{q}_t = q_t + a_T(T_b - T_t), \qquad (3)$$

where T_b is decimal time corresponding to June 30th of the designated base year (2002.5).

The logarithm of detrended daily concentrations are computed using Equation (1), with $\tilde{q}_t$ (detrended flow) and T_b (constant value for time) substituted for q_t and T_t, and by adding $\tilde{q}_t$ and an appropriate constant to obtain the logarithm of daily load. Because Equation (1) is in logarithmic units, adding $\tilde{q}_t$ to Equation (1) is similar to multiplying the concentration by flow. These estimates are converted from logarithm space to real space using methods described by Cohn (2005) and Schwarz *et al.* (2006). The detrended long-term mean annual load is computed by identifying those years included in the analysis period for which there are no days with

missing streamflow, summing the detrended daily load estimates for those days, and dividing by the number of included years to obtain mean load in units of kilograms per year.

Accuracy in Load Estimation

An important consideration in using estimates of detrended mean annual load in the development and calibration of SPARROW models is the accuracy of the load estimates estimated with Fluxmaster. The variance of the load estimates have a direct bearing on both the potential bias in the coefficients of SPARROW models and the standard errors of the SPARROW model predictions. Error in the load estimates can cause biases in the SPARROW model coefficient estimates if the errors in the load estimates are correlated with predictors in the SPARROW model (see the discussion in section 1.5.3.5 of Schwarz *et al.*, 2006). To understand this, note that the methodology used to estimate mean load causes the load estimate to be independent of its errors. Consequently, the error is not independent of the true mean load, as would be the case in a standard “errors in the variables” analysis, making the error a component of true load. As a component of true load, the error must be correlated with factors in the watershed that determine this load. If some of these factors are correlated with the explanatory variables in the SPARROW model, then the error in the mean load estimate is correlated with these explanatory variables, causing potential bias in the SPARROW model coefficient estimates.

An assumption of the SPARROW methodology is that the load estimate is measured without error (Schwarz *et al*., 2006). Accordingly, the variance of the error of a load prediction is computed from the squared differences between measured and model predicted load. However, if there are errors in the loads used in calibrating the SPARROW model, the true prediction error

variance should also account for the error between the measured and true loads. As explained above, the error in measured loads could be correlated with explanatory variables in the SPARROW model. If this is the case, then it can be shown (see section 1.6.6.1 of Schwarz *et al.*, 2006) that the error in measured loads are necessarily correlated with the residual in the SPARROW model. Because this covariance can be either positive or negative, the reported variance in the SPARROW model prediction error could be either an over- or under-estimate of the true variance.

Unfortunately, without knowledge of the true mean loads, it is not possible to obtain a direct estimate of either the bias in the coefficients or the bias in the reported variance of the SPARROW model prediction errors. It can be shown (Schwarz *et al.*, 2006) that both biases are bounded by a factor that is proportional to the variance in the errors of the measured loads, implying that these bounds uniformly go to zero as the variance of the error in the loads goes to zero. Therefore, quantifying the error in the measured mean loads and understanding the factors causing the errors are important for selecting only those stations with the greatest mean load accuracy, thereby limiting the bias in the SPARROW analysis.

Factors Affecting the Accuracy in Estimated Loads

One important question in designing monitoring programs is how important are various decisions used in deciding when to collect samples and how the length of water-quality and streamflow records affect the accuracy in the estimated loads. This information may be useful to future data compilation efforts and may help guide the design of water-quality monitoring strategies having the estimation of mean load as one of their objectives.

Because actual detrended mean annual loads are never observed, it is not possible to directly assess the accuracy of the loads estimated with Fluxmaster. Cohn (2005) shows that if the assumptions of the regression method used in the computations are valid (that is, Equations (1) and (2) are correctly specified for the full range of conditions experienced over the prediction period, with the residuals of equation (1) being independent, normally distributed and having a common variance) then the estimated detrended mean annual load is unbiased in the case of no censoring and only second-order biased (that is, as the number of measurements used to estimate the load model goes to infinity, bias goes to zero faster than the number of measurements goes to infinity) if there are censored water-quality measurements.

Using sequestered data techniques, Cohn *et al*. (1992) and Robertson and Roerish (1999) have show that the regression method used in Fluxmaster applied to infrequent measurements of water quality provides reasonably unbiased estimates of daily load. Robertson and Roerish found the median bias to be less than 10% in annual TP loads if the streams are sampled on a routine basis such as used in most monitoring programs. Stenback et al. (2011) also found little if any bias in the estimation in TP loads, but did not examine biases in TN loads.

As part of this study, we examined the accuracy of our Fluxmaster predictions. To evaluate the accuracy of the Fluxmaster estimates, daily loads for all load sites from all MRBs were predicted based on the water-quality model in Equation 1 and measured daily flows streamflows; loads were not detrended. In this analysis, only the water quality and streamflow data for a standard period from 1976 to 2004 were used. The minimum number of uncensored measurements required to estimate a mean annual load was set at 15 for all MRB's except MRB7 and MRB3, which used 20 and 25, respectively. Similar to the criterion used for including sites

in the SPARROW model, only sites that had a standard error less than 50% were included in the analysis.

Following Stenback et al. (2011), the accuracy of load estimates, particularly the accuracy of the retransformation factor used to convert estimates of load from logarithm to real space, was evaluated by comparing model-predicted nutrient loads to measured loads only on days in which water-quality data were collected. The performance of the model for sample days is assumed to be indicative of performance for days when no samples were obtained. The metric for the analysis is the ratio of the average of the predicted daily loads for a station to the average of the observed daily loads for the same observation days. Stenback et al. (2011) observed that if the variance of the residuals of the water-quality model are heteroscedastic, and if the heteroscedasticity is such that variance is greater at periods of low load, then the load estimation method developed by Cohn (2005) will overstate the retransformation factor for high load periods, causing significant upward bias in the estimates. Using the metric described above, Stenback et al. (2011) found evidence of this effect for nitrate but not for TP; TN was not included in their analysis.

The results of this analysis show little evidence of bias for TN (Figure S5-1). The graph of the logarithms of average observed and predicted loads for sample days displays a prominent one-to-one relation. The analogous graph for TP (Figure S5-2) shows evidence of greater variability, but the median bias was still < 8%. In general, there is a tendency for predicted loads to be underestimated. The downward bias in the TP load estimates appears to be limited to a subset of the stations. It is possible the bias is due to the failure of the linear model to account for changes in the flow-concentration relationship at high flows. Additional analysis of the estimates is required to better understand the cause of the bias.

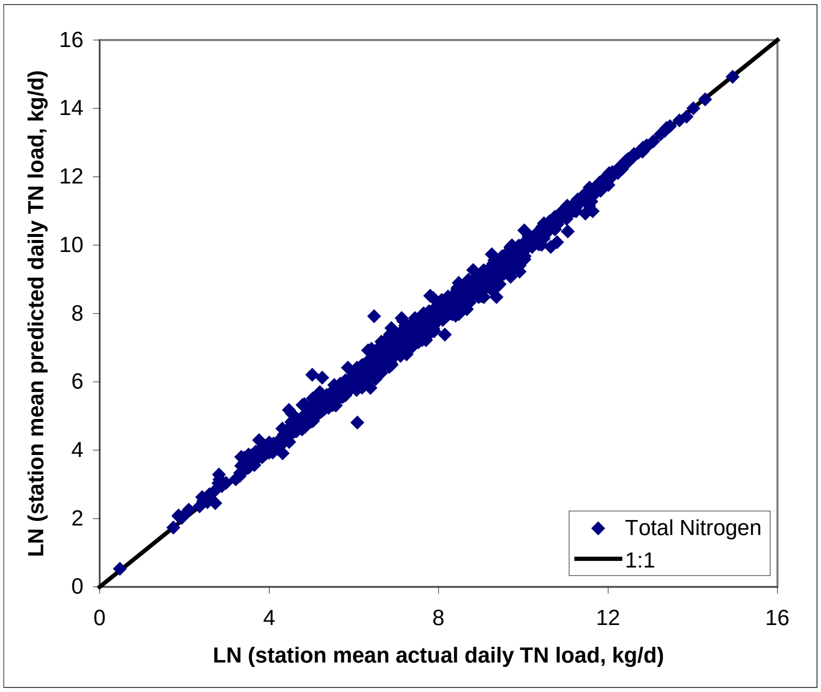

Figure S5-1. Natural log of the station mean actual daily TN load versus natural log of the station mean predicted daily TN load for all sample days for the period WY 1976-2004.

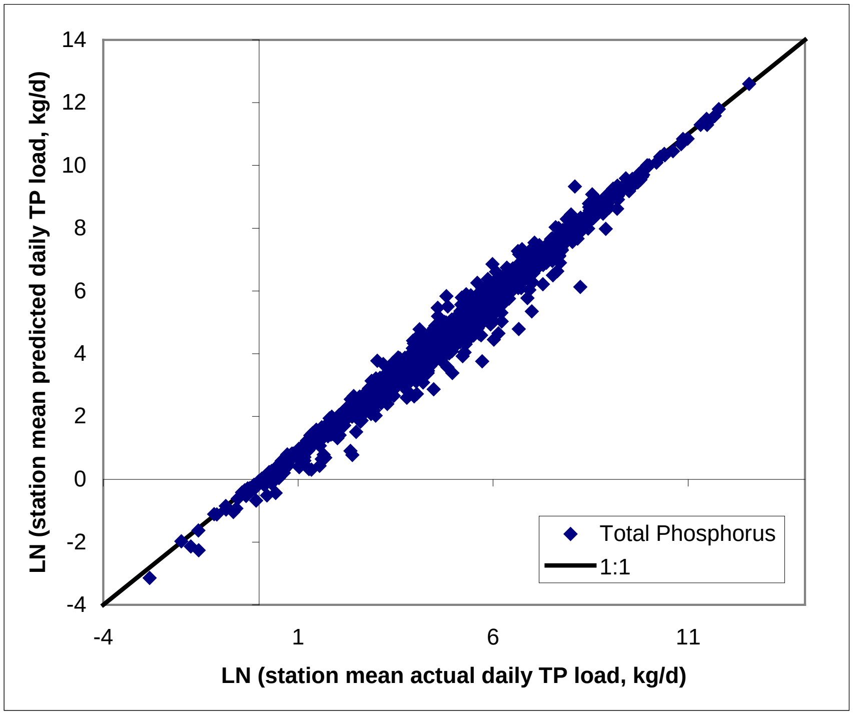

Figure S5-2. Natural log of the station mean actual daily TP load versus natural log of the station mean predicted daily TP load for all sample days for the period WY 1976-2004.

Overall, results of this analysis show that biases in Fluxmaster load estimations were relatively small: median bias for TN loads from 2,132 sites was < 1% and the median bias for TP loads from 2,460 sites was < 8%. The distribution of the biases in the TN and TP loads are shown in Table S5-1.

Table S5-1. Distribution of biases in predicted loads at each site. Bias was computed at each site as the average predicted daily load divided by the average measured daily load (only days with measured concentrations were included in the analysis). Numbers less than 1.0 indicate predicted loads are less than measured loads

	Percentiles for Biases							Average
Constituent	5th	10th	25th	50th	75th	90th	95th	Bias
Total Nitrogen	0.80	0.88	0.96	1.00	1.06	1.18	1.28	1.02
Total Phosphorus	0.56	0.66	0.80	0.92	1.00	1.07	1.16	0.90

Bias remains a concern, however, because in some cases the estimated loads are based on streamflow information that is extrapolated from a site that is not coincident with the water-quality measurement site. Lack of coincident sites creates potential adverse effects for the estimation of concentration and streamflow, the component determinants of load. For the estimation of concentration there is the possibility of a degraded correlation between water-quality and streamflow, a greater error in the estimates of the coefficients of Equation (1) and a larger mean square error for the estimated model. The degraded correlation in the flow-concentration relation and increased error in the water-quality model are fully accounted for in the estimation of load. These factors cause greater error in the load estimates, but they do not lead to any inherent biases in the loads or in the estimation in their errors.

The effect of non-coincident sites on streamflow is of greater concern. Streamflow at a non-coincident site is typically multiplied by an adjustment factor to approximate streamflow at the water-quality site, that factor being the ratio of the drainage areas at the two sites. Error in this factor is not accounted for by the load estimation methodology, and may introduce bias in both the load estimate and in the estimate of its error. Unfortunately, without a model of the

spatial error in streamflow, it is not possible to assess the loss in load accuracy in these cases, and the following analysis cannot address this concern. We note, however, that the SPARROW model residuals corresponding to stations with extrapolated streamflow may exhibit greater variation due to the extrapolated value, providing a means for evaluating the magnitude of this component of error.

Another component of load accuracy, not addressed in the following methodology, concerns the error caused by incomplete streamflow records. The SPARROW model relates long-term mean load to a set of basin attributes. Ideally, in order to facilitate the comparison of load across multiple stations, the mean load is computed over a period that is common to all stations. In practice, however, this is rarely the case, and the available prediction period is not the same for all stations. The assessment of accuracy described below takes the available flow record as given; it does not account for streamflow measurements that were not included in the record. This deficiency does not imply the load estimate is biased, but it does mean that the estimate of load accuracy is overstated if an incomplete prediction period is used. The simple streamflow model described in equation (2) could be used to generate streamflows for days missing a measurement, and those computed flows could be used to extend the prediction of daily load to include every day in the standard prediction period. Unfortunately, given the primitive nature of equation (2) it is unlikely that this approach would greatly improve the accuracy of the mean load estimate. A better approach would be to determine streamflow for missing days using streamflow measurements from nearby stations. However, as explained above in the case of non-coincident streamflow and water-quality stations, without a model that relates streamflows across multiple stations, such an approach is not possible. Given existing capabilities, the only way to control error due to an incomplete prediction period is to limit the stations included in the

SPARROW model to those having the longest records. Additional research is necessary to determine if this approach is sufficient.

In the absence of bias, estimates of the precision of the detrended mean load estimate are also estimates of the accuracy of the estimate. In the following, we shall refer to the accuracy of the detrended mean load estimate, with the understanding that this assessment is conditioned on the assumptions of the model given by equations (1) and (2) are valid and that, in the case of non-coincident water-quality and streamflow stations, the adjustment of streamflow is without error. Absent the validity of these assumptions, the estimate of the precision of mean load may also be biased making it impossible to assess either the accuracy or precision of the mean load estimate.

The standard error of the mean annual load estimate was computed using the method of Gilroy *et al.* (1990), extended to the case of censored water-quality data by Cohn (2005) and modified to accommodate the detrending of load (Schwarz *et al.*, 2006). The standard error is equal to the square root of the summation of the variances of the loads across all days in the prediction period plus the summation of two times the covariance of the estimated loads between each individual day in this period, normalized by the number of years in the prediction. Because detrended daily load is never directly observed, these variance and covariance terms must be derived from the model used to estimate daily load. Cohn (2005) shows that the estimated standard error of the mean annual load estimate is consistent if the residuals of the water-quality model given by Equation (1) adhere to the same assumptions used to derive the estimates.

The accuracy of the detrended mean annual load estimate primarily depends on the accuracy of the load predictions for the individual days included in the prediction period. Because streamflow is observed for all days, this component of load might be assumed to be

known without error, subject to the previously discussed qualifications regarding non-coincident water-quality and streamflow monitoring stations. However, for the estimation of detrended mean annual load, it is detrended streamflow that is used in place of actual streamflow and, as evident by equation (3), detrended streamflow depends on the estimated coefficient a_T. As explained in Schwarz *et al*. (2006), in the regression analysis of linear trend it is known that the trend coefficient is estimated with super-efficiency, meaning that the variance of the coefficient goes to zero at a rate of N^3, with N being the number of observations, rather than the usual rate of N for coefficients associated with non-trending variables (see, for example, Hamilton, 1994). Given the super-efficiency of the a_T estimate, and given that the number of observations used in the estimation of the streamflow trend (Equation (2)) is generally much greater than the number of observations available for the estimation of the water-quality model, it is statistically valid to treat detrended streamflow as known.

Conversely, the component of load due to contaminant concentration is not known, and must be estimated using Equation (1). Predictions based on this equation contain error, and it is this error that is the source of uncertainty in the mean load estimate. Because the concentration model is estimated in natural logarithm space, there is no simple relation between the uncertainty of predictions based on Equation (1) and uncertainty in predicted load in real space. Nevertheless, useful insight into the determinants of error in mean load can be obtained by identifying the primary determinants of error for predictions derived from Equation (1).

A linear regression model having an intercept term, such as Equation (1), can always be re-specified, without affecting prediction or model fit, such that the non-intercept explanatory variables are defined as differences from their respective sample means, and normalized by their respective sample standard deviations. A regression model specified in this way has a period-*i*

prediction error variance given by $\sigma^2\left(1+N^{-1}\left(1+\tilde{x}_i C^{-1}\tilde{x}_i'\right)\right)$, where σ^2 is the mean squared error of the model, N is the number of observations in the estimation sample, $\tilde{x}_i$ is the K-element row vector corresponding to the values of the K non-intercept explanatory variables used to make the prediction for day i, expressed as deviations from their respective estimation-sample means and divided by their estimation-sample standard deviations, and C is a K□K symmetric matrix corresponding to the estimation-sample correlations of the non-intercept explanatory variables. A first-order approximation of average prediction error variance, $\overline{V}^P$, can be derived by setting the off-diagonal correlation terms in C to zero and averaging over all days in the prediction period, to obtain

$$\overline{V}^P \approx \sigma^2\left(1+N^{-1}\left(1+\sum_{k=1}^{K}\left(V_k+\left|\overline{x}_k-\hat{\overline{x}}_k\right|^2\right)\Big/\hat{V}_k\right)\right), \qquad (4)$$

where V_k is the variance of explanatory variable k over the entire prediction period, $\hat{V}_k$ is the variance of explanatory variable k over the model estimation sample, $\overline{x}_k$ is the mean of explanatory variable k for the prediction period, and $\hat{\overline{x}}_k$ is the mean of explanatory variable k for the estimation sample. Note that the prediction period values of V_k and $\overline{x}_k$ differ from their estimation-sample equivalents not just because of differences in the days included in their estimation, but also because the prediction of detrended load uses different forms for some of the explanatory variables. For example, whereas actual streamflow and decimal time are used in model estimation, the estimation of detrended load uses detrended streamflow and the trend variable is a constant equal to the normalization date.

Although a precise equation for the standard error of detrended mean annual load cannot be expressed as simply as shown in Equation (4), the relationships implied by this equation provide meaningful guidance for specifying an empirical model of the determinants of the accuracy of mean load estimates. Equation (4) shows that the average variance of prediction error increases with: increases in the mean squared error of the model, decreases in the number of observations, increases in the variance of a an explanatory variable used in prediction, increases in the absolute difference between the explanatory variable means for prediction and model estimation, and decreases in the variance of an explanatory variable in the estimation sample. The following describes a simple model of the error of mean detrended load using explanatory variables obtained from Fluxmaster output that proxy those appearing in Equation (4).

The accuracy of the estimated mean annual load was evaluated using the coefficient of variation (COV), expressed as the ratio of the standard error of the mean load estimate divided by the estimate itself. It has been observed that the COV estimator is overly sensitive to unusual flow events and may produce extremely large, erroneous values, even if the load estimates are quite reasonable. Two extremely large TP COV observations in the MRB dataset were suspected to be the result of errors in the daily flows and were dropped from the analysis. The resulting analysis is based on mean load estimates from 2,107 stations for TN and 2,613 stations for TP. The mean COV for all included MRB sites was 0.108 for TN and 0.198 for TP, the median COV was 0.080 for TN and 0.133 for TP, the interquartile range was 0.051-0.130 for TN and 0.083-0.230 for TP, and the range was 0-1.78 for TN and 0.012-8.74 for TP. In agreement with the variability of the contaminant concentration in measurement samples, TP loads area estimated with significantly less accuracy than TN loads.

Nine factors were examined to determine their importance to the accuracy in load estimation: root mean square of the concentration-discharge relation (Equation 1), number of observations, percent of uncensored observations, length of the period with observations, maximum days between observations (largest gaps), variability in flow on observation days (coverage of flow regimes), variability in flow on prediction days (stream flashiness), flow-bias ratio (representativeness of flows sampled), and a binary variable to indicate whether or not the load estimated was detrended. Results of the regression analyses for TN and TP are presented in Table S5-2. The dependent variable in these analyses, the COV of the mean detrended load estimate, is highly skewed. In order to obtain homoscedastic residuals in the regression analysis, the dependent variable was transformed using a natural logarithm transformation, a common approach used in empirical work. Similarly, all continuous explanatory variables were also transformed using the natural logarithm function, implying that the model coefficients of these variables represent the percent change in COV corresponding to a 1% change in the associated, untransformed explanatory variable, holding constant the other factors in the analysis.

Table S5-2. Summary of regression results for the coefficient of variation of the log load estimate versus selected station attributes.
[significant explanatory variables (p-value less than 0.05) are highlighted in bold, red text; WQ, water-quality; SD, standard deviation]

	Log total nitrogen load coefficient of variation				Log total phosphorus load coefficient of variation			
Explantory Variable (station attribute)	Parameter estimate	Standard error	t-statistic	p-value	Parameter estimate	Standard error	t-statistic	p-value
Intercept	-0.210	0.125	-1.682	0.0926	1.302	0.120	10.811	**<.0001**
Log RMSE of WQ model	0.643	0.010	63.049	**<.0001**	1.266	0.023	54.588	**<.0001**
Log number of WQ observations	-0.420	0.016	-25.685	**<.0001**	-0.461	0.015	-30.067	**<.0001**
Log percent of uncensored WQ observations	-0.623	0.064	-9.783	**<.0001**	-0.327	0.039	-8.368	**<.0001**
Log SD of flow for WQ observation days	-0.189	0.016	-12.033	**<.0001**	-0.172	0.015	-11.834	**<.0001**
Log period length of WQ observations	0.024	0.023	1.053	0.2922	-0.076	0.021	-3.660	**0.0003**
Absolute value of log of flow bias ratio	0.170	0.029	5.809	**<.0001**	0.126	0.028	4.475	**<.0001**
Log maximum days between WQ observations	0.014	0.008	1.644	0.1004	0.022	0.008	2.944	**0.0033**
Log SD daily flow for prediction period	0.160	0.017	9.404	**<.0001**	0.151	0.016	9.603	**<.0001**
If load estimate is detrended	0.229	0.019	12.209	**<.0001**	0.305	0.017	17.630	**<.0001**
Number of stations	2,107				2,613			
Root mean squared error	0.369				0.380			
R^2	0.785				0.748			

A preliminary ordinary least squares (OLS) regression of the variables in Table S5-2 showed residuals having a pronounced pattern of spatial correlation, with residuals tending to be

correlated regionally, rather than within an individual basin. The best explanation of this correlation is that there were specific precipitation events, not adequately captured by the flow-related variables in Table S5-2, which had a pronounced effect on COV. Because such precipitation events tend to be regional rather than confined to an individual basin, the residuals would show a correlation pattern that was also regional, matching the observed pattern.

To correct for regional spatial correlation, we estimated the COV equation using a two-stage generalized least squares method, where a correlation matrix was developed from the preliminary OLS regression results. Each residual from the preliminary OLS regression was cross-multiplied by the residual of every other station to form $(N^2-N)/2$ unique products, each product being normalized by the mean squared error of the OLS residuals. Spatial correlation was assumed to be a function of the distance between stations, this function being continuous and piecewise linear for all distances greater than zero, with slope changes at threshold distances of 5, 10, 20, 40, 60, 80, 100, 120, 140, 160, 180, 200, 250, 300, 350, 400, 500, 600, and 700 kilometers. The correlation function for distances up to the first threshold was constrained to have a zero slope and was discontinuous with the correlation at distance zero, which was set to one. Correlations for distances greater than 700 kilometers were set to zero. The correlation for the first distance threshold and the slopes of the linearly segmented correlation function for the subsequent thresholds were determined by OLS regression of the normalized residual products on a suite of distance measures that were specified to give a continuous, piecewise linear relationship. The predictions from this regression were used to form the off-diagonal elements of the correlation matrix that was subsequently used in a second stage generalized least squares regression. A map of the transformed residuals (the residual vector pre-multiplied by the inverse of the root of the correlation matrix) showed no evidence of spatial correlation. The resulting

coefficient estimates, reported in Table S5-2, are consistent, efficient and normally distributed in large samples; the reported generalized least squares estimates of the coefficient standard errors are consistent.

Two variables reported by Fluxmaster and included as explanatory variables in Table S5-2 are direct measures of factors appearing in Equation (4): RMSE of the water-quality model residuals (the σ term appearing in Equation (4)), and the number of observations used to estimate the water-quality model (the N term in Equation (4)). From Equation (4), model RMSE is expected to have a positive effect of mean detrended load COV, and an increase in the number of observations should negative effect. The results of the analysis strongly support these expectations, as the coefficients of both variables, for both TN and TP, have the expected signs and are highly significant. The reported coefficients for RMSE indicate that a 1% increase in the RMSE results in increases of 0.6% and 1.3% in the COV of the mean load estimates for TN and TP, respectively, the largest responses of any variables included in the analysis. The coefficients for number of observations indicate that a 1% increase in N causes a 0.4% decrease in COV for TN and 0.5% decrease for TP.

Equation (4) pertains to prediction error from an OLS regression; however, some load estimates are derived using water-quality data that include censored observations. In these cases, the water-quality model is estimated with a Tobit maximum likelihood method (Cohn, 2005). Because a censored observation is not as informative as an actual value, coefficient estimates derived using censored data have less accuracy, an effect that is not evident from Equation (4). In this analysis, we use a measure of the degree to which censored data are used to compute the water-quality model: the logarithm of the percent of observations that are not censored. A larger percent of uncensored observations is expected to improve the accuracy of the mean load

estimate and the results support this conjecture (Table S5-2). The coefficients of the log of the percent of uncensored observations are negative and highly statistically significant. The results imply that a 1% increase in the percent of uncensored observations causes a 0.6% decrease in COV for TN and a 0.3% decrease for TP, among the largest responses found in the analysis.

Two variables in the regression models relate to the sample variance of the explanatory variables, the $\hat{V}_k$ terms in Equation (4). The logarithm of the standard deviation of streamflow for water-quality observation days is a close proxy for the variance of the logarithm of daily flow for these days, the exact variance required for Equation (4). The logarithm of the length of water-quality model sample period is a less precise analog of the variance of decimal time across sample days, the exact variance required in Equation (4) for the linear time trend variable in Equation (1) being unavailable. According to Equation (4), larger values of both variables are expected to reduce COV. These expectations are confirmed for the TP regression, which shows both variables to have statistically significant negative signs. The results for TN are less supportive. The log of the standard deviation of streamflow on water-quality observation days has the expected negative sign for TN, and is highly significant; however, the log of the length of the sample period has the wrong sign and is not significant. One problem with the log of the length of the sample period variable is that it may partly serve as a proxy for the absolute value of the difference in the prediction and sample means of the trend variable. In prediction, the trend variable is a constant equal to the normalization date. If the sample period is not centered on the normalization date, then an increase in its length implies a greater difference in the means. Because the absolute difference of means is expected to have a positive effect on COV, the combined effect of the length of the sample period could be either positive or negative.

Two variables in the analysis represent absolute differences in the prediction and sample period means (the $\left|\bar{x}_k - \hat{\bar{x}}_k\right|$ terms in Equation (4)). The first variable, the absolute value of the log of the flow-bias ratio, corresponds to the absolute value of the difference between the logarithms of mean streamflow for the prediction and sample periods. The magnitude of the coefficient is interpreted as the percent change in COV from a 1% increase in mean streamflow for either the prediction period or the sample period, whichever value is larger. From Equation (4), this variable is predicted to have a positive sign, and the statistically significant, positive coefficient estimates for both TN and TP strongly confirm this expectation (Table S5-2). Based on these results, a 1% increase in the larger of the prediction or sample period streamflow means causes a 0.2% increase in COV for TN and a 0.1% increase for TP.

The second variable in the analysis reflects a difference in the prediction and sample period means: the logarithm of the maximum number of days between water-quality observations (maximum gap in the water-quality observations). The larger the gap the greater is the likelihood that one or more of the seasons is not representatively sampled. The prediction period always includes complete years of record so the means of the sine and cosine variables used to measure the seasonal effects are necessarily zero. A large value for the maximum days between successive water-quality observations makes it likely that the means of the sine and cosine variables in the water-quality sample are not zero. Therefore, the expectation is that a larger gap in the sample record causes an increase in COV. The results of the analysis tend to confirm this expectation (Table S5-2). The coefficient estimates for the log of the maximum days between water-quality observations are positive for both TN and TP, although the estimate for TN is not statistically significant.

The log of the standard deviation of daily streamflow for the prediction period is a reasonable proxy for the variance of the log of detrended streamflow, one of the V_k terms in Equation (4). This variable is expected to have a positive effect on COV and the results of the analysis confirm this hypothesis (Table S5-2). The coefficients for both TN and TP are positive and highly statistically significant. The magnitudes of these coefficients imply that a 1% increase in the standard deviation of streamflow over the prediction period causes a 0.2% increase in both the TN and TP COVs. Because the time-trend variable is set to a constant in prediction, the prediction period variance of the time-trend term is zero and does not affect COV.

The remaining variable examined is a dichotomous indicator of whether or not the load estimate is detrended. This variable accounts for the fact that some mean load estimates are based on water-quality records that do not permit detrending of the estimate. These are cases where the water-quality record is too short to give a valid estimate of trend or the water-quality record does not sufficiently cover the chosen normalization date for the detrended estimates. In these cases, a trend coefficient is not included in the water-quality model described in Equation (1). Because the load estimate is based on fewer coefficients, there is one less term in the summation term given in Equation 4, and because each term must be positive, the marginal effect on COV is a decrease. This does not imply that the full effect of removing the trend term from the water-quality model is an increase in model accuracy. Equation (4) and the regression results in Table S5-2 take the RMSE of the water-quality model as given. The full effect of removing the trend variable would have to account for changes in the RMSE, and statistical theory states that the increase in the model RMSE must dominate the loss of a summation term in Equation (4), causing an overall increrase in prediction variance. Nevertheless, holding the RMSE fixed, the marginal effect of removing a variable from the water-quality model is an

expected increase in accuracy. The results of the analysis support this hypothesis (Table S5-2), as the coefficient for the dichotomous variable indicating that the load estimate is detrended is positive and highly significant for both TN and TP. Given a logarithm transformation applied to the dependent variable, 100 times the coefficient of a dichotomous variable is an approximate estimate of the percent change in the dependent variable from a change in the dichotomous variable from 0 to 1. The results of the analysis (Table S5-2) show that, holding RMSE fixed, the inclusion of a trend variable causes COV to increase by approximately 23% for TN and 31% for TP.

The results of this analysis provide some insight into the relative importance of some of the criteria used to select stations for inclusion in a SPARROW model. Of obvious importance is the number of observations, especially uncensored observations, available for estimation of the water-quality model. The number of observations has a quantitatively significant effect on the accuracy of the mean annual load estimate and selecting stations by this criterion is reasonable. Conversely, based on the quantitative estimates of the coefficients, a criterion based on the length of the sample period is of little relevance and should not be used. Of greater relevance are the sample characteristics of streamflow, both its mean and variance, as compared to streamflow characteristics for the prediction period. The results imply that water-quality samples that include large variability in streamflow, obtained by targeting specific flow events, but also selected to be representative of mean streamflow, produce more accurate estimates of mean load.

Literature Cited

Cohn, T.A., 2005, Estimating contaminant loads in rivers – an application of adjusted maximum likelihood to type 1 censored data. *Water Resources Research* 40(7): W07003.

Cohn, T., D. L. Caulder, E. J. Gilroy, L. D. Zynjuk, and R. M. Summers, 1992. The Validity of a simple statistical model for estimating fluvial constituent loads: an empirical study involving nutrient loads entering Chesapeake Bay, *Water Resources Research*, 28(9): 2353-2364.

Gilroy, E.J., R.M. Hirsch, and T.A. Cohn, 1990. Mean square error of regression-based constituent transport estimates. *Water Resources Research* 26(9): 2069-2077.

Hamilton, J.D., 1994. Time Series Analysis, Princeton, NJ, Princeton University Press: 799 p.

Robertson, D.M. and Roerish, E.D., 1999. Influence of various water quality sampling strategies on load estimates for small streams. *Water Resources Research*, 35(12): 3747-3759.

Schwarz, G.E., A.B. Hoos, R.B. Alexander, and R.A. Smith, 2006. The SPARROW Surface Water-Quality Model: Theory, Application and User Documentation. U.S. Geological Survey, Techniques and Methods Report, Book 6, Chapter B3, Reston, Virginia.

Stenback, G.A., W.G. Crumpton, K.E. Schilling, and M.J. Helmers, 2011. Rating Curve Estimation of Nutrient Loads in Iowa Rivers. *Journal of Hydrology* 396 (2011): 158-169.
